# Supplementary material for: Do private providers give patients what they demand, even if it is inappropriate? A randomised study using unannounced standardised patients in Kenya
Source: BMJ Open. 2022 Mar 18;12(3):e058746. doi: 10.1136/bmjopen-2021-058746 (PMC8935168; doi:10.1136/bmjopen-2021-058746)
Supplement: Supplementary data [file bmjopen-2021-058746supp001.pdf]

SUPPLEMENTAL APPENDICES

Supplement to:

Do private providers give patients what they demand, even if it is inappropriate? A randomized study utilizing unannounced standardized patients in Kenya

Ada Kwan[1,2], Claire E. Boone[2], Giorgia Sulis[3], and Paul J. Gertler[4]

[1]Division of Pulmonary and Critical Care Medicine, University of California San Francisco School of Medicine, San Francisco, CA, United States
[2]Division of Health Policy and Management, University of California, Berkeley School of Public Health, Berkeley, CA, United States
[3]Department of Epidemiology, Biostatistics, and Occupational Health, McGill University, Montreal, Canada
[4]Haas School of Business, University of California, Berkeley, Berkeley, CA, United States

Kwan A, *et al. BMJ Open* 2022; 12:e058746. doi: 10.1136/bmjopen-2021-058746

BMJ Publishing Group Limited (BMJ) disclaims all liability and responsibility arising from any reliance placed on this supplemental material which has been supplied by the author(s)

APPENDIX A: SUPPLEMENTAL METHODS ON STUDY CONTEXT, SP METHOD, AND ETHICS

Kwan A, *et al. BMJ Open* 2022; 12:e058746. doi: 10.1136/bmjopen-2021-058746

A1 Childhood diarrhea case management

Appendix Figure A1. Kenya national guidelines for health facility case management of childhood diarrhea

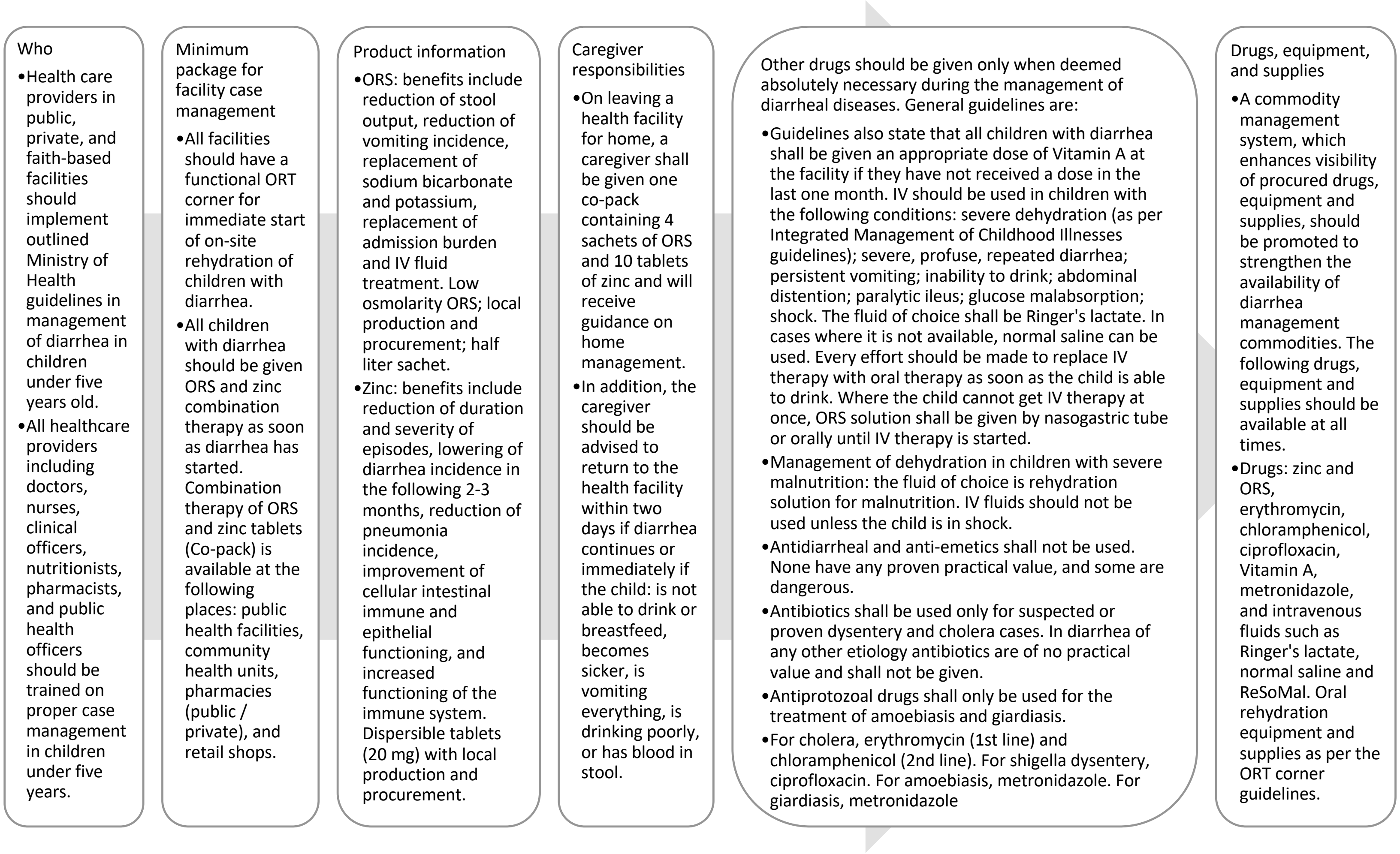

Kwan A, *et al. BMJ Open* 2022; 12:e058746. doi: 10.1136/bmjopen-2021-058746

A2 African Health Markets for Equity (AHME) Program and Evaluation

Details of the AHME intervention are provided where necessary for this study; however, the impact of the AHME intervention is not the purpose of this study. Across Kenya between 2013-2018, the AHME intervention was intended to "use National Health Insurance to link supply (private providers) with demand (clients) in order to shift health markets toward providing quality health care to low-income patients" . In Kenya, 56% of the population earns US$1 or less a day, and a third of the poor who are sick do not seek care, according to National Health Accounts data. To improve this situation, the AHME intervention is a mix of demand- and supply-side interventions implemented by non-governmental organizations in the context of a new scheme within the National Health Insurance Fund (NHIF). The demand- and supply-side interventions include: National Social Health Insurance scheme by Ministry of Health; SafeCare quality improvement by PharmAccess (similar to that described in Dunsch et al. [12]); Social franchising by Marie Stopes Kenya (MSK) and Population Services Kenya (PSK). The AHME impact evaluation's main aim is to assess the impacts and cost-effectiveness of the AHME intervention. Additionally, the evaluation aims to assess AHME's impact on several dimensions of quality including: (1) SafeCare's 680 measures of quality used in health facility assessments and quality improvement plans, (2) perceived quality measured by previous patients through a household survey, (3) perceived quality by existing patients through exit interviews, (4) provider knowledge through provider vignettes, (5) provider practice through SPs, and (6) patient safety outcomes through direct observation techniques and SPs.

A2.1. Experimental Design of the AHME Impact Evaluation

This section describes the AHME impact evaluation experimental design and clinic selection. Figure 1 shows a map of the clinics across Kenya alongside the process of clinic selection, which is described in detail as six steps below.

<u>Step 1. Clinics in Kenya listed.</u> Before the AHME program began in 2012, we mapped all clinics in 35 of Kenya's 47 counties with the goal of randomizing clinics eligible for the AHME program into treatment and control groups. Because there was no pre-existing list of private clinics at the time, clinics were first identified for mapping using four sources of information: (1) official government list of private clinics in the country, (2) clinics belonging to a major professional health associate (e.g., the Kenya Nurse and Midwives Association), (3) clinics that the AHME implementation partners suggested should be visited, and (4) additional clinics identified by evaluation teams in the field during the mapping process, but not included on any of the above lists. Government clinics (public clinics and hospitals), faith-based clinics (identified by clinic name), and clinics that were identified as franchised (by franchise branding on clinic exterior) were removed from the sampling frame generated from the aforementioned information sources.

<u>Step 2. Baseline clinic survey administered.</u> A pre-screening and baseline survey among remaining clinics was administered to remaining clinics. The purpose of this was to exclude clinics that the implementing partners indicated were not eligible for franchise services or the AHME set of interventions. Clinics that met the basic eligibility criteria still varied in their "level of eligibility" based on their existing capacity and suitability for franchising services and AHME interventions. Using additional criteria, created in a collaborative manner with MSK and PSK, clinics were further categorized into groups based on how likely they were to be eligible ("eligibility tiers") using data collected through the baseline survey instrument.

<u>Step 3. Randomization.</u> After clinics were categorized by eligibility tier, the research team conducted a stratified randomization of eligible clinics. For the randomization process, clinics were grouped into their

eligibility tiers within a county based on partner-provided criteria and data from the baseline survey, randomly ordered within strata (groups within which randomization would occur), and then randomly assigned to treatment (eligible to be offered AHME franchising immediately) or control (not eligible to be offered AHME until the completion of the study).

Steps 4 & 5. Clinics screened based on survey & site visit screening. After randomization procedures were completed, we provided MSK and PSK with partner-specific recruitment lists indicating the order in which clinics on their lists were to be approached for screening ("sensitization") and recruitment. Once randomization procedures had been completed, MSK and PSK began engaging clinics on their lists and proceeded with their respective recruitment procedures in October 2013. The second round of screening and recruitment by the partners served to identify clinics that were eligible for franchising and AHME interventions in the treatment arm. Eligible clinics that were invited to join either franchise ("ever franchised") were considered part of the evaluation sample in the treatment arm. Consistent with the intent-to-treat (ITT) assumptions applied in this study, these ever-franchised clinics were considered part of the final evaluation sample regardless of whether they completed the franchise enrollment process or maintained their franchise enrollment status for the entirety of the study period (for any reason).

Step 6. Final clinic sample for AHME impact evaluation. The final AHME evaluation samples were identified over the course of recruitment and honing activities. In total, 232 clinics were identified for the final evaluation sample (treatment clinics: N = 123; control clinics: N = 109). In September 2016, baseline data collection, including baseline household and client exit interviews, was completed for all AHME clinics.

The SP experiments were randomly assigned independent of the AHME treatment assignment. The table below shows the balance of AHME assignment across the SP demanding experiment assignments for our analytic sample. We include a AHME treatment indicator for analyses based on the clinic assignment to the AHME treatment or control group.

Appendix Table A1. Difference in means of AHME assignment by SP demanding experiment.

	**Clinics Assigned to Receive an SP Demanding Albendazole, n = 102**			**Clinics Assigned to Receive an SP Demanding Amoxicillin, n = 98**			
	N	*Mean*	*95% CI*	*N*	*Mean*	*95% CI*	*p-value*
Randomly assigned to receive AHME program	102	0.60	(0.50 - 0.69)	98	0.46	(0.36 - 0.56)	0.050

A3 Standardized Patient Recruitment, Training, and Pilot

We implemented two main survey methodologies: SPs and provider vignettes, which minimize bias in assessing provider practice and provider knowledge, respectively. For our SP data, we define visits as visits where the SP visited the clinic during operating hours and interacted with clinic staff, similar to an actual client presenting with similar conditions.

Other methods to assess quality of care have certain limitations that do not make them ideal for answering our research questions. Health and medical record data often do not exist in LMIC settings and when they do, they suffer from poor data quality. Direct observation is biased by the Hawthorne Effect. Patient exit interviews represent different patient sorting and patient mixes across clinics, and not only do clients not always understand medical jargon, but it is difficult to know precisely what medical condition the client has. Vignettes excel at assessing provider knowledge, but as for practice, vignettes are subject to social desirability bias and differ largely from practice measures (Kwan et al. 2019). For example, the "know-do gap" is a well-documented phenomenon in the literature referring to the difference between provider knowledge and provider practice (Das et al. 2015; Mohanan et al. 2015).

A3.1 SP Cases

We implemented our SP study based on protocol from a previous SP study conducted in Nairobi, Kenya (Daniels et al. 2017; Kwan et al. 2019). A technical advisory group consisting of four Kenyan clinicians advised our team on case development, and all hired SPs participated in developing standardized narratives (e.g., name, age, family situation, living situation, etc.) for the SP case during training. The technical advisory group participated in SP training and advised on outcome measures for each case.

The childhood diarrhea case scenario for SPs were adapted from a pilot conducted in 2014 in Nairobi, Kenya. The case scenario developed for this study has two parts: (1) the SP narrative designed with the technical advisory group and the SP recruits during training and (2) the corresponding SP exit survey. The SP narrative describes the social milieu of the presenting caregiver as well as the situation that motivates the caregiver to access health care services for their child who is sick at home with diarrhea on the day of the visit. Figure A3 shows the case scenario narrative. Figure A4 shows the case scenario's attire for presenting at each clinic, alongside the opening statement, and some history questions to which the SPs are trained to provide pre-scripted responses.

Appendix Figure A3. Childhood diarrhea case scenario narrative

SP NARRATIVE AHME (adapted from Nairobi)

Standardized Case 1: Watery Diarrhoea

JOSEPHINE

Josephine is 28 years old and a merchandiser in Nairobi. Today she decided to go home earlier than usual because she left her daughter Diana feeling unwell in the house. Diana has been unwell for the past two days and Josephine was really getting worried about her. Since then she had diarrhoea six or seven times and was crying more than usual. As Josephine was making her way home, she received a call from her niece, informing her that Diana's diarrhoea had worsened again. Her husband is away travelling.

Diana is one and a half years old. Lately Josephine has tried to observe cleanliness in the house. But recently there has been some water shortage due to a burst city council pipe. She buys 2-3 20-liter Jelicans of water[1] per day and divides it for all her chores. She stores the drinking water in a five-liter plastic can. She boils it when she has some paraffin to spare after making the family meals. There is no provision for drainage system, and they have one toilet that is shared among many households within the plot. There is also no system for garbage collection, and it is always heaped just behind her house. (Josephine lives in a small village. She gets water from a well/river nearby. She stores her drinking water in a small pot or a jelican. She boils it when she has some paraffin/wood to spare after making the family meals. There is no provision for a drainage system, and they have one toilet that is shared among many households.)

Diana is still breastfeeding, has had all of her immunizations and has been a healthy baby. Lately, she has not been her usual self, and since last night she is having several bouts of watery stools – sometimes they have mucus and stickiness. They did not smell particularly foul though. She seemed a little weak and tired but was still playful. Diana's body was a little hot. She has also been crying more than usual, and it seemed she had some tummy ache. She was vomiting a little. She lost her appetite but was drinking lots of water. Josephine had prepared ORS before she left for work for her to be given during the day. On hearing of the many episodes of diarrhoea that Diana has been having, Josephine was worried. She decided to visit a nearby clinic on her way home.

Appendix Figure A4. Case scenario attire, opening statement, and sample history question responses

SP NARRATIVE **AHME (adapted from Nairobi)**

Josephine's dress:

1. Generally, Josephine is a very simple woman.
2. Wears smart and casual clothes, which are not expensive.
3. She doesn't wear excessive make-up, and most times she does not wear any at all.
4. She puts on simple doll shoes or rubber shoes and small earrings or studs.
5. She carries a very simple handbag sometimes a shopping bag and a kikoi/kanga.
6. In the coast, Josephine wears a long dress/skirt, Dera or a Buibui. In some parts of Homabay and towards Kisii, she does not wear trousers at all. She only wears long skirts.

Opening statement:

My child has been having diarrhea.
Kiswahli: Mtoto wangu ana hara/endesha.
Taveta: Dakitari mwana wangu efwaka
Rabai: Dakitari mwanangu yunahara
Luhya: Omwana wanje anyalala
Kamba: Ndakitali mwana wakwa nukwitua
Meru: Kana gakwa igakwatwa/ mwana okwa nakwarwa
Kalenjin: Taktari mondoe moet lakwenyun
Maasai: Nkitari, keloito (e)nkeraiai (e)nkoshoke
Luo: Daktari, nyathina diewo
Kikuyu: Mwana wakwa niaraharwo
Taita: Dakitari mwana wapowawefwaya
Kisii: Omwana one agosaa
Embu: Ndakitare mwana wakwa nearavarwa

History questions asked by the provider and their answers:

1. Q: How old is the child?
   Mwanao ana umri gani?
   A: 1 1/2 years old.
   Mwaka mmoja unusi.

2. Q: How many times has she passed stools?
   Amehara mara ngapi?
   A: Many times.
   Mara nyingi.

3. Q: How many times in the last 24 hours?
   A: Maybe 6 or 7 times in the last two days.

4. Q: For how many days has she had this?
   Kwa muda wa siku ngapi amekuwa hivi?
   A: Two days ago but it wosened today.
   Siku mbili iliyopita lakini ilimzidia mchana.

A3.2 SP Recruitment and Training

Figure A5 shows the SP training agenda. SPs were extensively trained in risk mitigation sessions to avoid injections, taking medicine on the spot, unsafe and unsterile needles.

Appendix Figure A5. SP Training Agenda

SP TRAINING AGENDA

Week 1

	15-Jan-19 TUESDAY	16-Jan-19 WEDNESDAY	17-Jan-19 THURSDAY	18-Jan-19 FRIDAY
08.30 - 9:15	Registration and Introduction	Introduction to Group Work	Recap and presentation from each group	Recap and presentation from each group
09:15-10:30	Introduction to IPAK/ Admin	Review of SP cases in groups	Group work: Script and Narrative Development	Group reenactment of scripts using risk mitigation strategies
10:30 - 10:45	TEA BREAK	TEA BREAK	TEA BREAK	TEA BREAK
10:45 - 13:00	Introduction to IPAK/Admin / Introduction to SP cases	Review of SP cases in groups	Group reenactment of scripts and SP cases	Introduction to Exit Questionnaire
13:00 -14.00	LUNCH	LUNCH	LUNCH	LUNCH
14:00-15:00	Introduction to SP study	Group work: Script and Narrative Development	Risk Mitigation Strategies	Exit Questionnaire
15:00 - 16:00	Group SP into cases	Group work: Script and Narrative Development	Risk Mitigation Strategies	Exit Questionnaire
16:30 - 17:00	TEA BREAK	TEA BREAK	TEA BREAK	TEA BREAK

Week 2

	21-Jan-19 MONDAY	22-Jan-19 TUESDAY	23-Jan-19 WEDNESDAY	24-Jan-19 THURSDAY	25-Jan-19 FRIDAY
08.30 - 9:15	Exit Questionnaire	Mock interviews	Mock interviews for SP cases with practice recall questions	Clinicians Assessment of the SP/ mock interviews	Recap and presentation from each group
09:15-10:30					Mock interviews
10:30 - 10:45	TEA BREAK	TEA BREAK	TEA BREAK	TEA BREAK	TEA BREAK
10:45 - 13:00	Exit Questionnaire	Mock interviews	Mock interviews for SP cases with practice recall questions	Clinicians Assessment of the SP/ mock interviews	Mock interviews
13:00 -14.00	LUNCH	LUNCH	LUNCH	LUNCH	LUNCH
14:00-15:00	Introduction to Mock Interviews	Risk Mitigation Strategies	Mock interviews for SP cases with improvisation questions practice	Clinicians Assessment of the SP/ mock interviews	Mock interviews to practice recall questions
15:00 - 16:00	Mock interviews	Mock interviews	Mock interviews for SP cases with improvisation questions practice	Clinicians Assessment of the SP/ mock interviews	Mock interviews to practice recall questions
16:30 - 17:00	TEA BREAK	TEA BREAK	TEA BREAK	TEA BREAK	TEA BREAK

Week 3

	28-Jan-19 MONDAY	29-Jan-19 TUESDAY	30-Jan-19 WEDNESDAY	31-Jan-19 THURSDAY	01-Feb-19 FRIDAY
08.30 - 9:15	Risk Mitigation Strategies	Dry runs for the team	Dry runs for the team	Dry runs for the team	Final debriefing of the team
09:15-10:30					
10:30 - 10:45	TEA BREAK	TEA BREAK	TEA BREAK	TEA BREAK	TEA BREAK
10:45 - 13:00	Mock interviews to practice recall questions	Dry runs for the team	Dry runs for the team	Dry runs for the team	Screening of SPs by clinicians
13:00 -14.00	LUNCH	LUNCH	LUNCH	LUNCH	LUNCH
14:00-15:00	Mock interviews to practice recall and improvisation questions	Debriefing of the teams after dry runs	Debriefing of the teams after dry runs	Debriefing of the teams after dry runs	AX24 Last round of mock interviews practice recall and improvisation questions
15:00 - 16:00	Mock interviews to practice recall and improvisation questions	Debriefing of the teams after dry runs	Debriefing of the teams after dry runs	Debriefing of the teams after dry runs	Last round of mock interviews practice recall and improvisation questions
16:30 - 17:00	TEA BREAK	TEA BREAK	TEA BREAK	TEA BREAK	TEA BREAK

A3.3 SP Pilot and Demanding Experiment

Between February 5-15, 2019, the SPs piloted in Nairobi, and some teams also traveled out to three different areas in Kenya to ensure that we understood whether the experiments for the case needed to be adapted for different regions (since the clinic sample was spread across the country).

Given the experience during the pilot, we designed experiments for demanding unnecessary medicines. Figure A6 shows the case scenario narratives with scripts for the two experiments: demanding amoxicillin and demanding albendazole.

After the pilot and between fieldwork, the supervisors conducted refresher trainings in the classroom on the cases and did quality checks on the programmed SP exit questionnaire. Throughout fieldwork, the supervisors also conducted sessions where the case and experiments were reviewed again as a team to ensure there was no evolution of presentation in any given SP.

Kwan A, *et al. BMJ Open* 2022; 12:e058746. doi: 10.1136/bmjopen-2021-058746

Figure A6. Case Scenario Scripts for Demanding Unnecessary Medicine Experiments

Experiments for Standardized Case 1: Watery Diarrhoea

Only conduct the following experiments if assigned by your supervisor.

Experiment 1. Expressing Serious Concern and Demanding Unnecessary Drugs

Josephine goes to the facility, presents her case and on occasions when the provider refuses to give her anything/treat the baby, she tells him/her that she is really worried about her baby's condition and asks to be given (a) *dawa ya minyoo* (ABZ deworming medicines) or (b) Amoxyl (amoxicillin) for babies.

(a) **Demanding dawa ya minyoo (concern, abz):**
When assigned to this experiment, the SP presents worried and remembers another time when her baby was not feeling well and *dawa ya minyoo* calmed the baby. She wants the provider's advice and help in this concerning situation (otherwise she would just go to the chemist), but she is also convinced by her comfort that *dawa ya minyoo* will help.

At three possible moments when appropriate, the SP assigned this experiment can express the desire for *dawa ya minyoo*: (i) when the provider is writing a prescription or about to dispense drugs, (ii) when the doctor asks what the patient wants, or (iii) at the end of the interaction and if the provider hasn't given *dawa ya minyoo* (ABZ) yet, the SP stands up and seem as if she wants to leave then turn back and says to the doctor in a pleading tone:

"But doctor I'm really worried… Can you give me *dawa ya minyoo* for my baby? The last time she was sick, it helped."

(b) **Demanding Amoxyl (concern, amoxyl):**
When assigned to this experiment, the SP presents worried and remembers another time when her baby was not feeling well and *Amoxyl* calmed the baby. She wants the provider's advice and help in this concerning situation (otherwise she would just go to the chemist), but she is also convinced by her comfort that *Amoxyl* will help.

At three possible moments when appropriate, the SP assigned this experiment can express the desire for *Amoxyl*: (i) when the provider is writing a prescription or about to dispense drugs, (ii) when the doctor asks what the patient wants, or (iii) at the end of the interaction and if the provider hasn't given *dawa ya minyoo* (ABZ) yet, the SP stands up and seem as if she wants to leave then turn back and says to the doctor in a pleading tone:

"But doctor I'm really worried… Can you give me amoxyl for my baby? The last time she was sick, it helped."

We developed and finalized the SP script and demanding experiment together with a group of five field supervisors from Kenya, 40 individuals from Kenya who were recruited and hired to be standardized patients for this study (and approximately 60 more who were recruited and underwent partial training but not hired), and a technical advisory group of 4 health care providers who at the time of the study advised on national guidelines and actively trained cadres of health care providers. All of these individuals played a role in days of discussions and exercises during training on what medicines were trusted in the community and whether people in the community are open to using them. SPs and supervisors were involved in piloting the demanding of inappropriate medicines in the field. The team together acknowledged that amoxicillin and albendazole were common medicines, and their selection for study was not done arbitrarily. Further, we conducted the SP pilot with SPs demanding these two medicines

before the actual study. The selection of these two medicines in the script above were the result of the training and piloting process.

From the experience before fieldwork for this study, the SP recruits, supervisors, and our technical advisory group did not find that it was uncommon for patients in Kenya to ask for specific medicines they are familiar with. In particular, amoxicillin and albendazole are commonly prescribed drugs in the study setting, and thus presumed patients demanding either of those would not be seen as suspicious. It should be noted that the SP scripts were developed while taking into account local habits and behaviors in order to minimize the risk of SPs being identified as simulated, standardized patients.

When we began piloting the demanding experiment before fieldwork, we did not have the first two time points ((i) when the provider writes a prescription or is about to dispense drugs, (ii) when the provider asks what the patient wants). We only had the third (at the end of the interaction). However, the pilot anecdotally demonstrated to us that some providers did (i) and (ii) in the same moment, and for the SPs, it was unusual and out of their character to not respond if they came in "wanting the medicine they demanded".

It is quite possible that demanding a medicine when the provider is writing a prescription or about to dispense drugs could have an underlining incentive-induced difference. In this study, we assume that the different time points for demanding are balanced across each demanding arm.

A3.4 SP Fieldwork – Childhood Diarrhea Experiment Sample Size Calculations

There is some anecdotal evidence that suggests patients can be empowered with correct information to demand better services. At the same time, patients can demand unnecessary or potentially harmful care, such as broad-spectrum antibiotics. Our research question in this study examines how quality of care outcomes change if the patient demands inappropriate services (medicines) for the childhood diarrhea case scenario. This study was added to the endline data collection activities of the AHME program impact evaluation, which aimed to capture differences in quality of care due to AHME with SPs. To calculate MDE under different sample size and AHME program treatment effect scenarios, we utilized quality of care measures from a published SP study and included them as a benchmark for baseline measures and then chose sample sizes that made a best estimate of how much we would expect those outcomes of interest to move.

To calculate sample sizes, we conducted power calculations with minimum detectable effect (MDE) reported differences for a 1:1 randomly allocated SP demanding experiment to clinics independently randomized to receive the AHME program, see Appendix Table A2 below. MDE calculations assume 80% power, 5% alpha, varied differences between non-stratified and stratified control group taking on values {-0.10, -0.05, 0, 0.05, 0.10}, and are based on Daniels et al. (2017) who estimated the following correct management outcomes for private health facilities in Nairobi: 82% (SE: 7%) manage an asthma SP case with an inhaler or bronchodilator and 78% (SE: 8%) manage a childhood diarrhea SP case with oral rehydration salts. The quality of care differences we would be able to detect for the childhood diarrhea experiment would be 9-16%, respectively. For understanding differences in demanding unnecessary care vs. not across AHME treatment and control arms, we expected to answer this question with the original clinic sample with one visit per clinic.

Appendix Table A2. Power and Minimum Detectable Effect (MDE) Calculations

SP case	Power	Observations per Clinic	Total Clinics	Non-stratified	Varied Difference from Control	Control Proportion	MDE	Treatment Proportion

Diarrhea	0.8	0.2	47	0.78	-0.1	0.68	0.30	0.98
Diarrhea	0.8	0.5	117	0.78	-0.1	0.68	0.21	0.89
Diarrhea	0.8	1	234	0.78	-0.1	0.68	0.16	0.84
Diarrhea	0.8	1.5	351	0.78	-0.1	0.68	0.13	0.81
Diarrhea	0.8	2	468	0.78	-0.1	0.68	0.11	0.79
Diarrhea	0.8	0.2	47	0.78	-0.05	0.73	0.27	1.00
Diarrhea	0.8	0.5	117	0.78	-0.05	0.73	0.19	0.92
Diarrhea	0.8	1	234	0.78	-0.05	0.73	0.14	0.87
Diarrhea	0.8	1.5	351	0.78	-0.05	0.73	0.12	0.85
Diarrhea	0.8	2	468	0.78	-0.05	0.73	0.11	0.84
Diarrhea	0.8	0.2	561	0.78	0	0.78	0.10	0.88
Diarrhea	0.8	0.5	117	0.78	0	0.78	0.17	0.95
Diarrhea	0.8	1	234	0.78	0	0.78	0.13	0.91
Diarrhea	0.8	1.5	351	0.78	0	0.78	0.11	0.89
Diarrhea	0.8	2	468	0.78	0	0.78	0.10	0.88
Diarrhea	0.8	0.2	561	0.78	0.05	0.83	0.09	0.92
Diarrhea	0.8	0.5	117	0.78	0.05	0.83	0.15	0.98
Diarrhea	0.8	1	234	0.78	0.05	0.83	0.11	0.94
Diarrhea	0.8	1.5	351	0.78	0.05	0.83	0.10	0.93
Diarrhea	0.8	2	468	0.78	0.05	0.83	0.09	0.92
Diarrhea	0.8	0.2	561	0.78	0.1	0.88	0.08	0.96
Diarrhea	0.8	0.5	561	0.78	0.1	0.88	0.08	0.96
Diarrhea	0.8	1	234	0.78	0.1	0.88	0.09	0.97
Diarrhea	0.8	1.5	351	0.78	0.1	0.88	0.08	0.96
Diarrhea	0.8	2	468	0.78	0.1	0.88	0.07	0.95

A4 Ethical Clearance

Ethical considerations for utilizing the SP method for this study

The AHME quantitative evaluation was granted clearance by the ethics committees at Kenya Medical Research Institute (No. KEMRI/RES/7/3/1; NON-SSC PROTOCOL NO. 372) and the Human Subjects Committee for Innovations for Poverty Action IRB-USA (IPA IRB Protocol #1085). The ethical clearance included all primary data collection activities for process quality analyses. This appendix describes the protocol for SP data collection.

All the SPs in this study were hired as field staff and participated in a three-week training, and a two-week pilot, and are required to participate in refresher trainings throughout fieldwork in order to mitigate any potentially harmful events, such as unsafe injections, invasive tests, and consumption of any medicines during encounters in the health sector.

Similar to other SP studies with similar designs and embedded in an intervention,[6] we sought a waiver of provider informed consent to conduct the SP study. The request for a waiver was based on a recent study commissioned by the United States Department of Health and Human Services to assess the ethics of simulated patient studies.[7] Supported by a pilot study conducted in Nairobi that validated the SP method in the Kenyan context,[8] both ethics committees approved the waiver request within the AHME evaluation study because (1) combining informed consent with the congregation of providers during trainings and the implementation of interventions during the study period posed threats to the scientific validity of the study objectives, as well as to the risk of SP detection, and (2) there is no more than minimal risk of participation to the SPs or providers, as reported in the Nairobi SP pilot and validation study (Daniels et al. 2017).

Ethics committee approvals with the waiver of informed consent were provided conditional on our agreement to return to all clinics visited by SPs to disclose the SP study to them and to provide them with an opportunity to ask questions and discuss any concerns. During January 1–23, 2020, we informed all clinics that received SPs and that were not closed permanently at that time.

All full questionnaires, case scripts, and the granted request for a waiver of informed provider consent are available upon request.

A5 Fieldwork Protocol and Details

Clinics were assessed for SP visit eligibility and mapping was also conducted to determine which clinics had been closed. The following protocol was implemented ahead of fieldwork initiation:

- Ineligible – SPs should not visit any clinics that are labelled as such in the schedule. For example,
    - 2 clinics – do not accept walk-ins from individuals who are not employees of a firm only
    - 1 clinic – fistula clinic
    - 1 clinic – excluded due to security issues
    - There could be more.
- Check closed status – Supervisors should double check whether these are closed at the time of fieldwork before sending any SPs
    - 8 clinics based on mapping activity
    - There could be more.
- Non-consenting clinics – Supervisors should check mapping and other surveys implemented for the AHME impact evaluation (exit interviews; clinic surveys) to see whether consent has been provided for at least one of these AHME surveys at the time of SP fieldwork. If consent has not been provided at any of these, do not conduct any SP interactions.

To reduce SP detection at the clinic while maintaining fieldwork protocol, the following were implemented:

1. Familiars at sampled clinics. Before conducting any interactions in each region, QAs should introduce the full clinic list to the SPs. The clinics should be described one-by-one. SPs should review all the clinics in the sample and identify to the QA any clinics where they know a friend or family member who is affiliated to that clinic. The QA.
2. SP case narratives. When a team arrives at a new fieldwork area, the QA should debrief the full team on: (i) the area, (ii) the clinics in the area, (iii) contextual adaptations, including dress and language, to each of the cases based on the new fieldwork.
3. Isolated/rural clinics. Isolated/rural clinics should be identified by each QA from their mapping experience. For each SP that goes to that clinic, a story should be constructed for three things: (i) from where the SP character is traveling, (ii) to where the SP character is traveling, (iii) local names of people and places the SP character visited or will visit.
4. SP visit timing. The childhood diarrhea case should be sent in the afternoon.
5. SP sequencing. SP cases or SPs who have a lower risk of detection should be sent before SP cases or SPs who have a higher risk of detection.
6. SP spacing. The spacing between SP visits should be controlled by the QA. First, more than one SP should not be sent at the same time unless the QA knows that there are 5 or more patients waiting to be seen at a given time on a given day. Second, QAs should wait 2-3 days between SP interactions at clinics that see <5 patients per day or do not have strangers coming for services.

Kwan A, *et al. BMJ Open* 2022; 12:e058746. doi: 10.1136/bmjopen-2021-058746

APPENDIX B: SUPPLEMENTAL RESULTS

Appendix Figure B1. Childhood diarrhea knowledge vs. practice

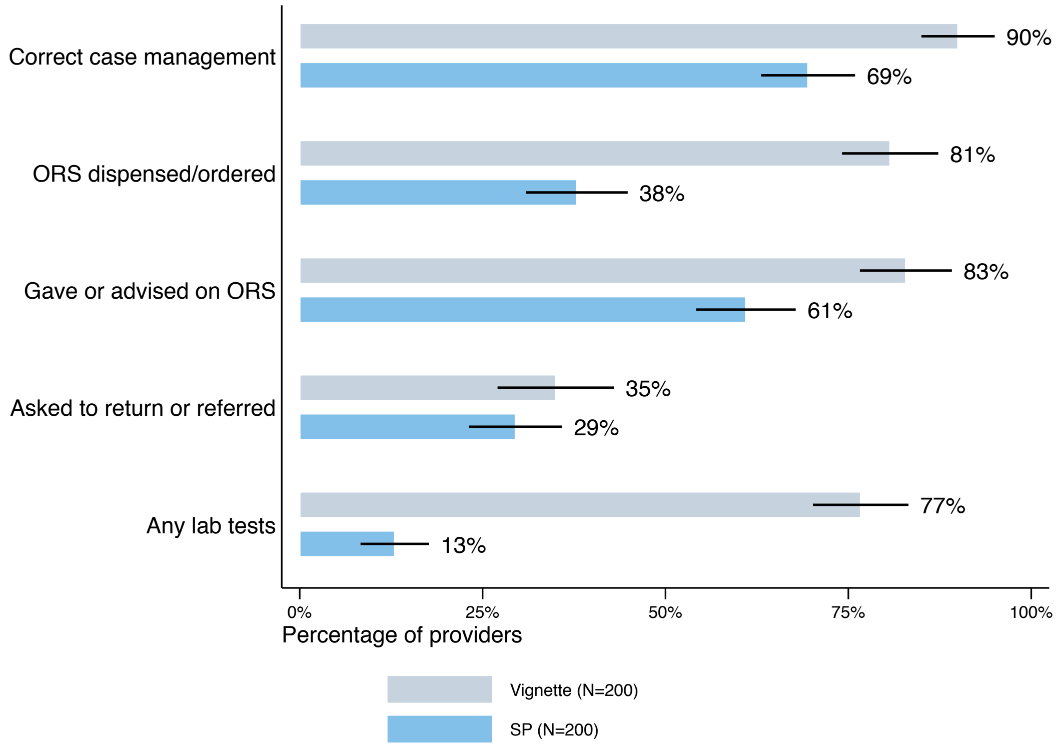

Appendix Figure B2. Effects Pre-demanding Amoxicillin vs. Pre-Demanding Albendazole (n = 200)

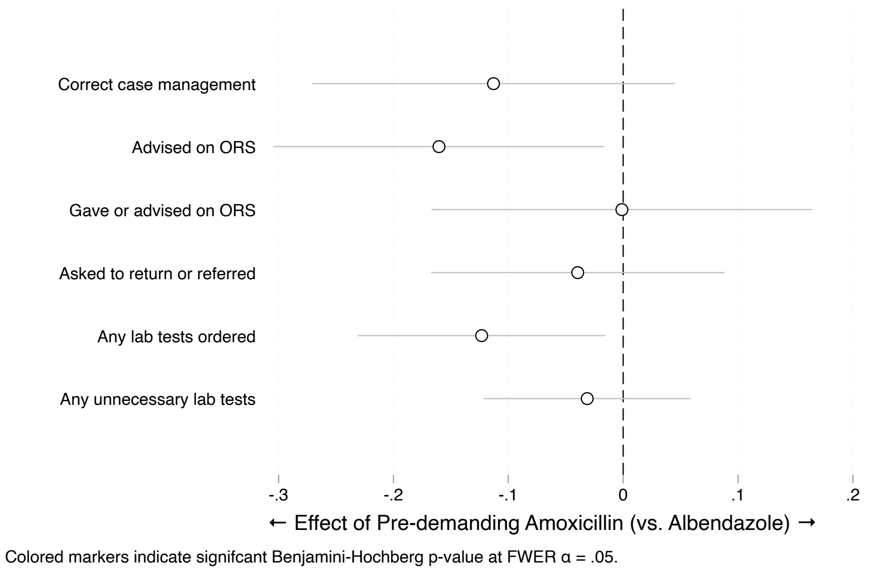

Appendix Figure B3. Effects of Demanding (Pooled Albendazole and Amoxicillin, n = 400)

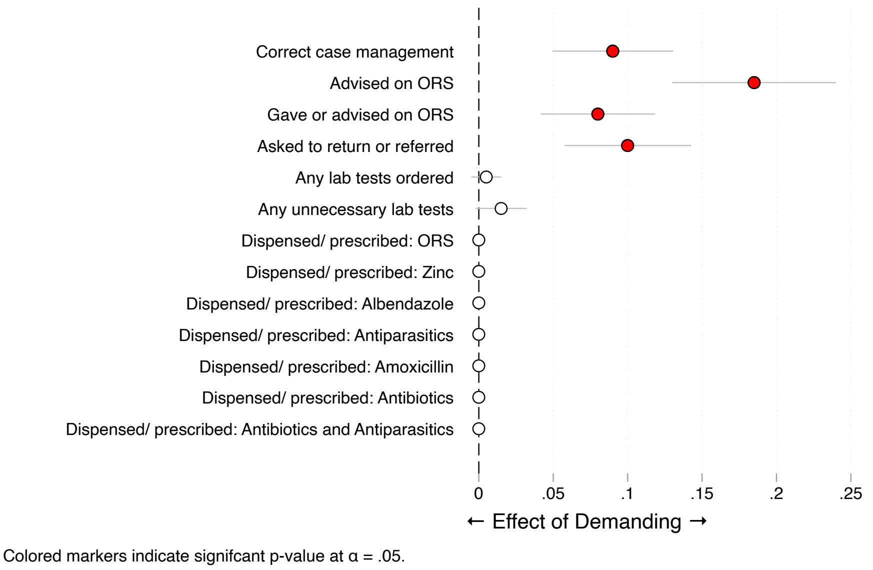

Appendix Table B1. Effects of demanding albendazole or amoxicillin vs. pre-demanding on quality of care outcomes

(A) Post-demanding (n=200) and pre-demanding (n=200) without AHME and demanding interactions

	(1) Correct case management	(2) Asked to return or referred	(3) Asked to return	(4) Referred elsewhere	(5) Gave or advised on ORS	(6) Advised on ORS	(7) Dispensed/ prescribed: ORS	(8) Dispensed/ prescribed: Zinc	(9) Number of medicines
Albendazole post-demanding									
Coefficient	0.117	0.110	0.123	0.014	0.053	0.198	-0.035	-0.060	0.010
Standard Error	(0.036)	(0.038)	(0.038)	(0.015)	(0.038)	(0.042)	(0.032)	(0.032)	(0.134)
p-value	[0.001]	[0.004]	[0.001]	[0.352]	[0.166]	[0.000]	[0.279]	[0.063]	[0.939]
Amoxicillin post-demanding									
Coefficient	0.062	0.090	0.076	-0.015	0.108	0.171	0.037	0.063	-0.011
Standard Error	(0.038)	(0.040)	(0.040)	(0.016)	(0.041)	(0.047)	(0.034)	(0.034)	(0.140)
p-value	[0.101]	[0.026]	[0.060]	[0.351]	[0.009]	[0.000]	[0.281]	[0.065]	[0.939]
AHME treatment									
Coefficient	-0.009	0.024	0.093	-0.001	-0.069	-0.167	0.085	0.007	0.027
Standard Error	(0.064)	(0.056)	(0.057)	(0.033)	(0.069)	(0.061)	(0.069)	(0.071)	(0.278)
p-value	[0.886]	[0.669]	[0.105]	[0.985]	[0.313]	[0.006]	[0.221]	[0.923]	[0.923]
Observations	400	400	400	400	400	400	400	400	400
Pre-demanding Group Mean	0.705	0.245	0.255	0.060	0.570	0.448	0.360	0.390	2.375

	(10) Number of efficacious medicines	(11) Number of non-efficacious medicines	(12) Any non-efficacious medicines	(13) Dispensed/ prescribed: Albendazole	(14) Dispensed/ prescribed: Amoxicillin	(15) Dispensed/ prescribed: Antibiotics	(16) Dispensed/ prescribed: Antiparasitics	(17) Dispensed/ prescribed: Antibiotics & Antiparasitics
Albendazole post-demanding								
Coefficient	-0.096	0.106	-0.015	0.142	-0.017	0.008	0.122	0.094
Standard Error	(0.060)	(0.105)	(0.033)	(0.027)	(0.019)	(0.034)	(0.029)	(0.027)
p-value	[0.112]	[0.315]	[0.638]	[0.000]	[0.383]	[0.810]	[0.000]	[0.001]
Amoxicillin post-demanding								
Coefficient	0.100	-0.110	0.016	-0.148	0.018	-0.009	-0.127	-0.098
Standard Error	(0.063)	(0.109)	(0.034)	(0.027)	(0.020)	(0.036)	(0.029)	(0.028)
p-value	[0.114]	[0.313]	[0.640]	[0.000]	[0.383]	[0.810]	[0.000]	[0.000]
AHME treatment								
Coefficient	0.092	-0.065	-0.104	-0.003	0.004	-0.030	-0.056	-0.058
Standard Error	(0.127)	(0.217)	(0.068)	(0.054)	(0.040)	(0.072)	(0.061)	(0.057)
p-value	[0.471]	[0.765]	[0.127]	[0.959]	[0.913]	[0.680]	[0.353]	[0.314]
Observations	400	400	400	400	400	400	400	400
Pre-demanding Group Mean	0.750	1.625	0.670	0.190	0.095	0.555	0.245	0.205

Note: The table shows ordinary least squares regressions using standardized patient (SP) data. Robust standard errors are in parentheses, clustered at the clinic level (2 observations corresponding to 1 SP visit per clinic). Two-sided p-values in brackets. All models contain SP fixed effects and control for the 0-1 AHME treatment indicator, a binary indicator for whether a clinic was assigned to receive an SP demanding albendazole at the end of the visit (Albendazole post-demanding) or whether a clinic was assigned to receive an SP demanding amoxicillin at the end of the visit (Amoxicillin post-demanding). All outcomes in models (1)-(17) are binary variables where if the action occurred during the visit 1=yes; 0=otherwise for both pre-demanding and post-demanding time points for the visit. Correct case management is a binary outcome for whether any one of the following actions were performed according to guidelines: asked to return, referred elsewhere, gave ORS, or advised on ORS. ORS is oral rehydration salts. Antiparasitics include antimalarials. "Dispensed/prescribed: Antibiotics & Antiparasitics" refers to whether the provider gave any antibiotic and any antiparasitic.

(B) Post-demanding (n=200) and pre-demanding (n=200) with AHME and demanding interactions

	(1) Correct case management	(2) Asked to return or referred	(3) Asked to return	(4) Referred elsewhere	(5) Gave or advised on ORS	(6) Advised on ORS	(7) Dispensed/ prescribed: ORS	(8) Dispensed/ prescribed: Zinc	(9) Number of medicines
Albendazole post-demanding									
Coefficient	0.132	0.098	0.112	0.014	0.092	0.218	-0.059	-0.110	-0.254
Standard Error	(0.059)	(0.059)	(0.057)	(0.027)	(0.063)	(0.063)	(0.056)	(0.059)	(0.214)
p-value	[0.027]	[0.100]	[0.053]	[0.592]	[0.144]	[0.001]	[0.299]	[0.063]	[0.237]
Albendazole * AHME treatment									
Coefficient	-0.027	0.023	0.020	-0.001	-0.067	-0.033	0.040	0.084	0.444
Standard Error	(0.073)	(0.077)	(0.077)	(0.032)	(0.077)	(0.086)	(0.070)	(0.071)	(0.275)
p-value	[0.718]	[0.768]	[0.794]	[0.981]	[0.384]	[0.700]	[0.569]	[0.242]	[0.109]
Amoxicillin post-demanding									
Coefficient	0.067	0.057	0.046	-0.011	0.099	0.171	0.045	0.085	0.196
Standard Error	(0.052)	(0.049)	(0.048)	(0.021)	(0.055)	(0.065)	(0.043)	(0.045)	(0.165)
p-value	[0.195]	[0.249]	[0.346]	[0.591]	[0.073]	[0.009]	[0.298]	[0.062]	[0.236]
Amoxicillin * AHME treatment									
Coefficient	-0.011	0.069	0.065	-0.007	0.023	0.001	-0.020	-0.050	-0.454
Standard Error	(0.075)	(0.079)	(0.081)	(0.032)	(0.081)	(0.091)	(0.070)	(0.070)	(0.286)
p-value	[0.884]	[0.380]	[0.424]	[0.817]	[0.776]	[0.993]	[0.777]	[0.481]	[0.115]
AHME treatment									
Coefficient	0.000	0.001	0.071	0.001	-0.059	-0.159	0.080	-0.001	0.035
Standard Error	(0.070)	(0.056)	(0.056)	(0.033)	(0.073)	(0.065)	(0.069)	(0.071)	(0.277)
p-value	[0.999]	[0.986]	[0.206]	[0.965]	[0.421]	[0.014]	[0.246]	[0.991]	(0.277)
Observations	400	400	400	400	400	400	400	400	400
Pre-demanding Group Mean	0.705	0.245	0.255	0.060	0.570	0.448	0.360	0.390	2.375

	(10) Number of efficacious medicines	(11) Number of non-efficacious medicines	(12) Any non-efficacious medicines	(13) Dispensed/ prescribed: Albendazole	(14) Dispensed/ prescribed: Amoxicillin	(15) Dispensed/ prescribed: Antibiotics	(16) Dispensed/ prescribed: Antiparasitics	(17) Dispensed/ prescribed: Antibiotics & Antiparasitics
Albendazole post-demanding								
Coefficient	-0.169	-0.085	-0.094	0.176	-0.030	-0.057	0.143	0.118
Standard Error	(0.105)	(0.165)	(0.053)	(0.049)	(0.030)	(0.058)	(0.053)	(0.050)
p-value	[0.111]	[0.607]	[0.080]	[0.000]	[0.321]	[0.329]	[0.008]	[0.019]
Albendazole * AHME treatment								
Coefficient	0.124	0.320	0.132	-0.059	0.022	0.110	-0.037	-0.042
Standard Error	(0.129)	(0.216)	(0.067)	(0.058)	(0.039)	(0.072)	(0.063)	(0.060)
p-value	[0.339]	[0.140]	[0.050]	[0.311]	[0.579]	[0.129]	[0.559]	[0.479]
Amoxicillin post-demanding								
Coefficient	0.130	0.066	0.072	-0.136	0.023	0.044	-0.111	-0.091
Standard Error	(0.081)	(0.128)	(0.041)	(0.040)	(0.023)	(0.045)	(0.042)	(0.039)
p-value	[0.109]	[0.607]	[0.081]	[0.001]	[0.323]	[0.329]	[0.009]	[0.021]
Amoxicillin * AHME treatment								
Coefficient	-0.070	-0.384	-0.124	-0.023	-0.012	-0.115	-0.033	-0.012
Standard Error	(0.129)	(0.225)	(0.069)	(0.056)	(0.041)	(0.072)	(0.061)	(0.057)
p-value	[0.589]	[0.090]	[0.072]	[0.684]	[0.770]	[0.112]	[0.585]	[0.841]
AHME treatment								
Coefficient	0.080	-0.044	-0.104	0.017	0.002	-0.027	-0.039	-0.044
Standard Error	(0.127)	(0.217)	(0.067)	(0.057)	(0.040)	(0.072)	(0.063)	(0.059)
p-value	[0.531]	[0.839]	[0.124]	[0.764]	[0.955]	[0.709]	[0.538]	[0.451]
Observations	400	400	400	400	400	400	400	400
Pre-demanding Group Mean	0.750	1.625	0.670	0.190	0.095	0.555	0.245	0.205

Note: The table shows ordinary least squares regressions using standardized patient (SP) data. Robust standard errors are in parentheses, clustered at the clinic level (2 observations corresponding to 1 SP visit per clinic). Two-sided p-values in brackets. All models contain SP fixed effects and control for the 0-1 AHME treatment indicator, a binary indicator for whether a clinic was assigned to receive an SP demanding albendazole at the end of the visit (Albendazole post-demanding) or whether a clinic was assigned to receive an SP demanding amoxicillin at the end of the visit (Amoxicillin post-demanding). Models also include interactions between the AHME treatment and each of the demanding experiments. All outcomes in models (1)-(17) are binary variables where if the action occurred during the visit 1=yes; 0=otherwise for both pre-demanding and post-demanding time points for the visit. Correct case management is a binary outcome for whether any one of the following actions were performed according to guidelines: asked to return, referred elsewhere, gave ORS, or advised on ORS. ORS is oral rehydration salts. Antiparasitics include antimalarials. "Dispensed/prescribed: Antibiotics & Antiparasitics" refers to whether the provider gave any antibiotic and any antiparasitic.

Appendix Table B2. Effects of post-demanding albendazole (vs. post-demanding amoxicillin) on childhood diarrhea care management outcomes

(A) Post-demanding (n=200) without AHME and demanding interactions

	(1) Correct case management	(2) Asked to return or referred	(3) Asked to return	(4) Referred elsewhere	(5) Gave or advised on ORS	(6) Advised on ORS	(7) Dispensed/ prescribed: ORS	(8) Dispensed/ prescribed: Zinc	(9) Number of medicines
Albendazole post-demanding									
Coefficient	0.052	0.014	0.046	0.034	-0.074	-0.015	-0.086	-0.148	0.025
Standard Error	(0.073)	(0.076)	(0.077)	(0.040)	(0.081)	(0.080)	(0.081)	(0.081)	(0.331)
p-value	[0.473]	[0.853]	[0.553]	[0.391]	[0.360]	[0.854]	[0.287]	[0.071]	[0.940]
AHME treatment									
Coefficient	-0.022	0.049	0.115	-0.003	-0.083	-0.168	0.092	0.018	0.025
Standard Error	(0.064)	(0.067)	(0.068)	(0.035)	(0.071)	(0.071)	(0.071)	(0.072)	(0.292)
p-value	[0.727]	[0.466]	[0.091]	[0.926]	[0.248]	[0.019]	[0.200]	[0.800]	[0.932]
Observations	200	200	200	200	200	200	200	200	200
Demanding Amoxicillin Group Mean	0.755	0.296	0.286	0.051	0.673	0.582	0.398	0.449	2.347

	(10) Number of efficacious medicines	(11) Number of non-efficacious medicines	(12) Any non-efficacious medicines	(13) Dispensed/ prescribed: Albendazole	(14) Dispensed/ prescribed: Amoxicillin	(15) Dispensed/ prescribed: Antibiotics	(16) Dispensed/ prescribed: Antiparasitics	(17) Dispensed/ prescribed: Antibiotics & Antiparasitics
Albendazole post-demanding								
Coefficient	-0.234	0.259	-0.038	0.348	-0.041	0.020	0.299	0.230
Standard Error	(0.147)	(0.257)	(0.078)	(0.059)	(0.049)	(0.084)	(0.068)	(0.066)
p-value	[0.113]	[0.314]	[0.631]	[0.000]	[0.397]	[0.810]	[0.000]	[0.001]
AHME treatment								
Coefficient	0.110	-0.085	-0.101	-0.030	0.008	-0.031	-0.080	-0.075
Standard Error	(0.130)	(0.226)	(0.069)	(0.052)	(0.043)	(0.074)	(0.060)	(0.058)
p-value	[0.398]	[0.708]	[0.147]	[0.570]	[0.859]	[0.671]	[0.188]	[0.197]
Observations	200	200	200	200	200	200	200	200
Demanding Amoxicillin Group Mean	0.847	1.500	0.704	0.031	0.112	0.551	0.133	0.122

Note: The table shows ordinary least squares regressions using standardized patient (SP) data for the post-demanding phase of the N=200 SP visits (1 observation corresponds to 1 SP visit per clinic). Standard errors are in parentheses. Two-sided p-values in brackets. All models contain SP fixed effects and control for the 0-1 AHME treatment indicator, a binary indicator for whether the visit was Albendazole post-demanding (if 0, the visit was Amoxicillin post-demanding). All outcomes in models (1)-(17) are binary variables where if the action occurred during by the end of the visit 1=yes; 0=otherwise. Correct case management is a binary outcome for whether any one of the following actions were performed according to guidelines: asked to return, referred elsewhere, gave ORS, or advised on ORS. ORS is oral rehydration salts. Antiparasitics include antimalarials. "Dispensed/prescribed: Antibiotics & Antiparasitics" refers to whether the provider gave any antibiotic and any antiparasitic.

(B) Post-demanding (n=200) with AHME and demanding interactions

	(1) Correct case management	(2) Asked to return or referred	(3) Asked to return	(4) Referred elsewhere	(5) Gave or advised on ORS	(6) Advised on ORS	(7) Dispensed/ prescribed: ORS	(8) Dispensed/ prescribed: Zinc	(9) Number of medicines
Albendazole post-demanding									
Coefficient	0.064	0.033	0.064	0.031	-0.022	0.006	-0.117	-0.217	-0.450
Standard Error	(0.099)	(0.102)	(0.104)	(0.054)	(0.109)	(0.109)	(0.109)	(0.110)	(0.445)
p-value	[0.519]	[0.745]	[0.541]	[0.572]	[0.840]	[0.954]	[0.284]	[0.049]	[0.313]
Albendazole * AHME treatment									
Coefficient	-0.022	-0.037	-0.034	0.007	-0.100	-0.040	0.060	0.134	0.913
Standard Error	(0.127)	(0.132)	(0.134)	(0.070)	(0.141)	(0.140)	(0.141)	(0.142)	(0.574)
p-value	[0.865]	[0.778]	[0.797]	[0.918]	[0.478]	[0.774]	[0.672]	[0.347]	[0.113]
AHME treatment									
Coefficient	-0.012	0.067	0.132	-0.007	-0.034	-0.148	0.062	-0.047	-0.420
Standard Error	(0.089)	(0.093)	(0.094)	(0.049)	(0.099)	(0.099)	(0.099)	(0.100)	(0.404)
p-value	[0.894]	[0.472]	[0.164]	[0.890]	[0.733]	[0.135]	[0.528]	[0.639]	[0.300]
Observations	200	200	200	200	200	200	200	200	200
Demanding Amoxicillin Group Mean	0.755	0.296	0.286	0.051	0.673	0.582	0.398	0.449	2.347

	(10) Number of efficacious medicines	(11) Number of non-efficacious medicines	(12) Any non-efficacious medicines	(13) Dispensed/ prescribed: Albendazole	(14) Dispensed/ prescribed: Amoxicillin	(15) Dispensed/ prescribed: Antibiotics	(16) Dispensed/ prescribed: Antiparasitics	(17) Dispensed/ prescribed: Antibiotics & Antiparasitics
Albendazole post-demanding								
Coefficient	-0.335	-0.115	-0.173	0.365	-0.059	-0.099	0.299	0.244
Standard Error	(0.198)	(0.345)	(0.105)	(0.080)	(0.066)	(0.113)	(0.092)	(0.089)
p-value	[0.094]	[0.738]	[0.101]	[0.000]	[0.373]	[0.380]	[0.001]	[0.007]
Albendazole * AHME treatment								
Coefficient	0.193	0.720	0.260	-0.031	0.034	0.229	0.001	-0.028
Standard Error	(0.256)	(0.444)	(0.135)	(0.104)	(0.085)	(0.145)	(0.119)	(0.115)
p-value	[0.451]	[0.107]	[0.056]	[0.763]	[0.693]	[0.116]	[0.994]	[0.808]
AHME treatment								
Coefficient	0.016	-0.436	-0.227	-0.015	-0.009	-0.143	-0.080	-0.062
Standard Error	(0.180)	(0.313)	(0.095)	(0.073)	(0.060)	(0.102)	(0.084)	(0.081)
p-value	[0.931]	[0.165]	[0.018]	[0.842]	[0.884]	[0.163]	[0.341]	[0.446]
Observations	200	200	200	200	200	200	200	200
Demanding Amoxicillin Group Mean	0.847	1.500	0.704	0.031	0.112	0.551	0.133	0.122

Note: The table shows ordinary least squares regressions using standardized patient (SP) data for the post-demanding phase of the N=200 SP visits (1 observation corresponds to 1 SP visit per clinic). Standard errors are in parentheses. Two-sided p-values in brackets. All models contain SP fixed effects and control for the 0-1 AHME treatment indicator, a binary indicator for whether the visit was Albendazole post-demanding (if 0, the visit was Amoxicillin post-demanding). Models also include interactions between the AHME treatment and the Albendazole post-demanding experiment. All outcomes in models (1)-(17) are binary variables where if the action occurred during by the end of the visit 1=yes; 0=otherwise. Correct case management is a binary outcome for whether any one of the following actions were performed according to guidelines: asked to return, referred elsewhere, gave ORS, or advised on ORS. ORS is oral rehydration salts. Antiparasitics include antimalarials. "Dispensed/prescribed: Antibiotics & Antiparasitics" refers to whether the provider gave any antibiotic and any antiparasitic.

Appendix Table B3. Composition of medicines prescribed across all SPs who demanded albendazole versus amoxicillin.

Type	Medicine	Demanding Albendazole (N=102)		Demanding Amoxicillin (N=98)	
		Frequency	*Percentage*	*Frequency*	*Percentage*
Correct	ORAL REHYDRATION SALTS	32	31%	38	39%
	ZINC	33	32%	42	43%
	ORAL REHYDRATION SALTS AND ZINC SULPHATE	2	2%	2	2%
Antiparasitic	ALBENDAZOLE	35	34%	3	3%
	ARTEMETHER LUMEFANTRINE	5	5%	4	4%
	QUININE	1	1%	0	0%
	DILOXANIDE	0	0%	4	4%
	DIHYDROARTEMISININ AND PIPERAQUINE PHOSPHATE	0	0%	1	1%
	NITAZOXANIDE	0	0%	1	1%
Antibiotic	METRONIDAZOLE	27	26%	27	28%
	SULFAMETHOXAZOLE AND TRIMETHOPRIM	17	17%	21	21%
	METRONIDAZOLE BENZOATE	12	12%	12	12%
	AMOXICILLIN	8	8%	11	11%
	DILOXANIDE FUROATE METRONIDAZOLE DICYCLOMINE HCL	6	6%	2	2%
	AMPICILLIN AND CLOXACILLIN	3	3%	3	3%
	CEFALEXIN	2	2%	2	2%
	ERYTHROMYCIN	2	2%	2	2%
	CEFIXIME	2	2%	0	0%
	CHLORAMPHENICOL PALMITATE	1	1%	2	2%
	AMOXICILLIN AND POTASSIUM CLAVULANATE	1	1%	0	0%
	CIPROFLOXACIN	1	1%	0	0%
	CHLORAMPHENICOL	1	1%	0	0%
	DILOXANIDE FUROATE METRONIDAZOLE	1	1%	0	0%
	ERYTHROMYCIN ETHYL SUCCINATE	1	1%	0	0%
	CHLORAPHENICOL AND RETINOL	0	0%	1	1%
	ROXITHROMYCIN	0	0%	1	1%
	AMPICILLIN	0	0%	1	1%
	CEFADROXIL	0	0%	1	1%

Other	PARACETAMOL	22	22%	23	23%
	IBUPROFEN	5	5%	3	3%
	LEVAMISOLE	4	4%	0	0%
	IBUPROFEN AND PARACETAMOL	3	3%	1	1%
	HYOSCINE BUTYLBROMIDE	2	2%	0	0%
	PROMETHAZINE HYDROCHLORIDE	1	1%	5	5%
	SACCHAROMYCES BOULARDII	1	1%	1	1%
	MULTIVITAMIN	1	1%	1	1%
	KAOLIN PECTIN	1	1%	0	0%
	AMINOSIDINE	1	1%	0	0%
	CHLORPHENIRAMINE	1	1%	0	0%
	CETIRIZINE	1	1%	0	0%
	DICYCLOVERINE HYDROCHLORIDE SIMETHICONE	1	1%	0	0%
	GUAIFENESIN	1	1%	0	0%
	VITAMIN A	1	1%	0	0%
	LOPERAMID HYDROCHLORIDE	0	0%	2	2%
	DOMPERIDONE	0	0%	1	1%
	PIROXICAM	0	0%	1	1%
	SALBUTAMOL	0	0%	1	1%
Unknown	UNKNOWN	0	0%	1	1%

Kwan A, *et al. BMJ Open* 2022; 12:e058746. doi: 10.1136/bmjopen-2021-058746
