# Supplementary material for: Do private providers give patients what they demand, even if it is inappropriate? A randomised study using unannounced standardised patients in Kenya
Source: BMJ Open. 2022 Mar 18;12(3):e058746. doi: 10.1136/bmjopen-2021-058746 (PMC8935168; doi:10.1136/bmjopen-2021-058746)
Supplement: Author's manuscript [file bmjopen-2021-058746.draft_revisions.pdf]

BMJ Open is committed to open peer review. As part of this commitment we make the peer review history of every article we publish publicly available.

When an article is published we post the peer reviewers' comments and the authors' responses online. We also post the versions of the paper that were used during peer review. These are the versions that the peer review comments apply to.

The versions of the paper that follow are the versions that were submitted during the peer review process. They are not the versions of record or the final published versions. They should not be cited or distributed as the published version of this manuscript.

BMJ Open is an open access journal and the full, final, typeset and author-corrected version of record of the manuscript is available on our site with no access controls, subscription charges or pay-per-view fees (http://bmjopen.bmj.com).

If you have any questions on BMJ Open's open peer review process please email info.bmjopen@bmj.com

BMJ Open

Do private providers give patients what they demand, even if it is inappropriate? A randomized study utilizing standardized patients in Kenya

Journal:	*BMJ Open*
Manuscript ID	bmjopen-2021-058746
Article Type:	Original research
Date Submitted by the Author:	18-Nov-2021

Complete List of Authors:	Kwan, Ada; University of California San Francisco, Division of Pulmonary and Critical Care Medicine; University of California Berkeley School of Public Health, Division of Health Policy and Management<br>Boone, Claire; University of California Berkeley School of Public Health, Division of Health Policy and Management<br>Sulis, Giorgia ; McGill University, Department of Epidemiology, Biostatistics and Occupational Health<br>Gertler, Paul; University of California Berkeley Haas School of Business

Keywords:	Health policy < HEALTH SERVICES ADMINISTRATION & MANAGEMENT, Health economics < HEALTH SERVICES ADMINISTRATION & MANAGEMENT, Quality in health care < HEALTH SERVICES ADMINISTRATION & MANAGEMENT, International health services < HEALTH SERVICES ADMINISTRATION & MANAGEMENT, Public health < INFECTIOUS DISEASES

SCHOLARONE™
Manuscripts

I, the Submitting Author has the right to grant and does grant on behalf of all authors of the Work (as defined in the below author licence), an exclusive licence and/or a non-exclusive licence for contributions from authors who are: i) UK Crown employees; ii) where BMJ has agreed a CC-BY licence shall apply, and/or iii) in accordance with the terms applicable for US Federal Government officers or employees acting as part of their official duties; on a worldwide, perpetual, irrevocable, royalty-free basis to BMJ Publishing Group Ltd ("BMJ") its licensees and where the relevant Journal is co-owned by BMJ to the co-owners of the Journal, to publish the Work in this journal and any other BMJ products and to exploit all rights, as set out in our licence.

The Submitting Author accepts and understands that any supply made under these terms is made by BMJ to the Submitting Author unless you are acting as an employee on behalf of your employer or a postgraduate student of an affiliated institution which is paying any applicable article publishing charge ("APC") for Open Access articles. Where the Submitting Author wishes to make the Work available on an Open Access basis (and intends to pay the relevant APC), the terms of reuse of such Open Access shall be governed by a Creative Commons licence – details of these licences and which Creative Commons licence will apply to this Work are set out in our licence referred to above.

Other than as permitted in any relevant BMJ Author's Self Archiving Policies, I confirm this Work has not been accepted for publication elsewhere, is not being considered for publication elsewhere and does not duplicate material already published. I confirm all authors consent to publication of this Work and authorise the granting of this licence.

For peer review only

Do private providers give patients what they demand, even if it is inappropriate? A randomized study utilizing standardized patients in Kenya

Ada Kwan[1,2], Claire E. Boone[2], Giorgia Sulis[3], and Paul J. Gertler[4]

[1]Division of Pulmonary and Critical Care Medicine, University of California San Francisco School of Medicine, San Francisco, CA, United States
[2]Division of Health Policy and Management, University of California, Berkeley School of Public Health, Berkeley, CA, United States
[3]Department of Epidemiology, Biostatistics, and Occupational Health, McGill University, Montreal, Canada
[4]Haas School of Business, University of California, Berkeley, Berkeley, CA, United States

For peer review only

Abstract

Introduction: Low and varied quality of care has been demonstrated for childhood illnesses in low-and middle-income countries. Some quality improvement strategies focus on increasing patient engagement; however, evidence suggests that patients demanding medicines can favor the selection of resistant microbial strains in the individual and the community if drugs are inappropriately used. This study examines the effects on quality of care when patients demand different types of inappropriate medicines.

Methods: We conducted an experiment where standardized patients (SPs), locally recruited individuals trained to simulate a standardized case, present at private clinics. Between March 8 and May 28, 2019, 10 SPs portraying caretakers of a watery diarrhea childhood case scenario (in absentia) conducted N=200 successful SP-provider visits at 200 private, primary care clinics in Kenya. Half of the clinics were randomly assigned to receive an SP demanding amoxicillin (an antibiotic); the other half, an SP demanding albendazole (an antiparasitic drug often used for deworming), with other presenting characteristics the same. We used logistic and linear regression models to assess the effects of demanding these inappropriate medicines on correct and unnecessary case management outcomes.

Results: Demanding albendazole significantly increased its rate of dispensing to 35% (95% CI: 25-44) compared to 3% (95% CI: 0-7) among those who didn't demand it (adjusted odds ratio 0.06, 95% CI:0.02-0.23, p<0.001). Providers did not give different levels of amoxicillin to those who demanded it and those who did not demand it (adjusted odds ratio 1.73, 95% CI: 0.50-5.98). Neither significantly changed any correct management outcomes, such as treatment or referral elsewhere.

Conclusion: Private providers appear to account for both business-driven benefits and individual health impacts when making prescribing decisions. Additional research is needed on provider knowledge and perceptions of profit and individual and community health trade-offs when making prescription decisions after patients demand inappropriate care.

Keywords: prescribing practices, diagnostics, standardized patients, quality, health care providers, Kenya, private health sector

Abstract word count: 299

Word count: 4933 (with article summary)

Corresponding Author:

Ada Kwan, PhD MHS

Postdoctoral Scholar

University of California, San Francisco

Department of Medicine

1001 Potrero Avenue

San Francisco, CA 94110

ada.kwan@ucsf.edu

For peer review only

Strengths and limitations of this study

- Using the standardized patient method, where locally recruited and trained individuals present with a standardized case scenario at private primary care clinics in Kenya, this study reports the extent to which provider treatment behaviors are influenced by patient demand for treatment that are unnecessary, harmful to the community, yet provided as empiric treatment for acute childhood watery diarrhea.
- We compared the impact of a patient demanding an antibiotic medicine (amoxicillin, which has known public health risks for the individual and community), to an antiparasitic medication (albendazole) which is perceived to be harmless to the individual but also poses a risk to the community.
- Our study cannot compare levels of services without demanding, because by design, the SPs demanded medicines at the end of the visit, though on some occasions the SPs had to demand earlier to ensure that there was a consistent though standardized narrative for the visit.
- Because of the standardized presentation of the childhood diarrhea case scenario used in this study, we are able to know what happens across multiple providers when they are presented with the same case.
- A better understanding of the extent to which individual and community consequences can be realized in settings with growing or dominant private health sectors will help in preventing drug resistance globally.

For peer review only - http://bmjopen.bmj.com/site/about/guidelines.xhtml

Introduction

Individuals seeking health care services sometimes demand inappropriate medicines, such as antibiotics, based on the widespread misperception that this would lead to faster and better recovery.[1,2] Regardless of adequate training and knowledge of clinical practice guidelines, providers may grant these requests to facilitate their patients feeling satisfied and to avoid negative judgments.[3] For-profit providers may be concerned that these negative judgments, and their overall reputation, can reduce the likelihood of patients returning for subsequent visits, which can affect their bottom line. These behaviors may vary based on the extent to which medicines demanded are harmful or perceived as such and, in the private sector, profitable.[4–8] In this paper, we study the effects of a patient demanding two different inappropriate medicines, as examples of trade-offs providers might make between risks, profits, and patient satisfaction.[6,7,9,10]

Understanding the relationship between inappropriate dispensing behaviors and what patients demand from providers is important for designing quality improvement interventions. Public health authorities and many studies cite the overuse and misuse of antimicrobials as the main drivers of drug-resistance.[11] However, there is at best limited literature on the effects of when patients demand medicines on provider prescribing behavior in low- and middle-income countries (LMICs).[6,7] On the patient side, studies in high-income countries suggest that both patient and provider knowledge, attitudes, and expectations are important drivers of antibiotic prescriptions.[12–14] One example is the notion of patient activation, or when "patients who have the *motivation, knowledge, skills, and confidence* to make *effective* decisions to manage their health" (emphasis ours).[15] Patient activation has been extensively studied in the United States, and this research emphasizes the potential for interventions that increase informed, and "active" patients, particularly because of its association with better health and health care outcomes.[16,17] However, patient activation is different from when patients demand antibiotics that are inappropriate for their conditions. Further, many of these studies report associations and cannot differentiate whether increased engagement results in increased quality of care or the reverse. Thus, constructing effective interventions on patient engagement becomes challenging if actors or mechanisms for intervening to improve care are unclear. This suggests that the patient's role could have a larger influence on better care relative to the provider. That patients can have a larger influence on services begs the question, What happens when patients demand different inappropriate care?

The objective of our study is to examine the role of patient demand for inappropriate care, on prescribing and dispensing practices for childhood diarrhea in Kenya. We utilize the standardized patient (SP) method, which provides an unbiased way to compare multiple providers because of a standardized case scenario presentation. We draw on childhood diarrhea for several reasons. First, several studies have validated the use of the SP method for examining childhood diarrhea, including in Kenya.[18–22] The SP method has many advantages over other existing quality of care methods and also controls for patient mix and sorting. For example, provider surveys measure provider knowledge rather than actual practice; exit interviews suffer from recall bias and clients may also not be able to discern specific clinical actions; providers may perform differently under direct observation, known as the Hawthorne effect; and in these settings, the quality of administrative data or records is often varied, if it exists at all.[8,22] Accruing evidence from SP studies on childhood diarrhea across LMICs demonstrate that quality of care is low and varied for correct management of childhood illnesses.[18,19,23–28]

For peer review only - http://bmjopen.bmj.com/site/about/guidelines.xhtml

Second, although the global burden of diarrheal disease is declining over time, it remains a major concern particularly in LMICs where poor sanitation and hygiene along with indiscernibly varied quality health care make this health condition among children common and often life-threatening. With 1.73 billion episodes a year, diarrhea remains one of the leading causes of morbidity and mortality in children worldwide.[29,30]

Third, diarrhea is an interesting condition to examine the role of patient demand on appropriate and inappropriate care. Diarrhea is defined as an increase in frequency of bowel movements (usually three or more per day), accompanied by a decrease in stool consistency.[31] Although a wide range of pathogens can cause diarrheal disease, consumption of contaminated food or water and interpersonal contacts in poor hygiene conditions constitute a common denominator. Rotavirus, *Escherichia coli*, *Cryptosporidium spp.* and *Shigella spp.* are the most common causal agents in lowest income settings.[32] The World Health Organization (WHO) Integrated Management for Childhood Illness (IMCI) handbook was published in 2005 to provide a structured and simplified approach to the assessment and therapeutic management of children presenting with various clinical pictures in first-level primary care facilities, particularly in resource-limited areas.[33] With respect to diarrhea, antimicrobial treatment is only recommended under selected circumstances (e.g., evidence of blood in the stool).

This study contributes to the literature in several ways. First, this study adds to the understanding of how pervasive is overprescription. Recent studies on health conditions, including common childhood illnesses, in LMICs show that the overuse of medicines pose dangers of resistance that have individual and public health level consequences.[23,34] Second, we provide experimental evidence on patient-related determinants that are shown to influence appropriate and inappropriate treatment.[1,2,35] Two other studies to our knowledge examine the effects of patient demand on the rates of antibiotic dispensing with the SP method and find that (i) SPs who share knowledge that antibiotics are inappropriate during a visit in China were less likely to receive antibiotics, and (ii) the rates of antibiotic prescriptions reduced when SPs demanded them alongside a statement that they would make the purchase elsewhere.[6,7] In an LMIC setting, which is underrepresented in the literature on this topic, this study additionally extends the current literature on the role of caregivers demanding two types of antimicrobial medicines for a condition that, for most children with this condition, requires only supportive treatment. For example, the majority of diarrhea cases only require support treatment, such as rehydration, and do not need antimicrobial therapy.[31,33] Third, this study can help inform governments that are committed to universal access to high quality of care worldwide. Understanding how quality of care can be improved is critical, particularly in the private sector in countries where a substantial amount of care is provided through the private sector.(36) This study contributes to the knowledge of improving quality improvement by understanding the provision of support treatment, as well as the provision of unnecessary or potentially harmful treatment at point of care.

Methods

This study examines standardized patient (SP) and provider vignette data collected in 2019 across 200 private clinics spread across 35 of Kenya's 47 counties. **Figure 1** shows a map of Kenya and the clinic sample. For this study, we exploited the 2014-2019 AHME evaluation

For peer review only - http://bmjopen.bmj.com/site/about/guidelines.xhtml

private clinic sample (N=232) and did not visit clinics that were ineligible to receive walk-ins for childhood illnesses (4), were located in an area with security concerns (1), did not consent to the AHME evaluation study (7), or were permanently closed (14). We did not capture data from 6 clinics, which turned away the SP upon arrival. Additional details related to the program and clinic sample are provided in Appendix A as they are relevant for this study. Patients or the public were not involved in the design, or conduct, or reporting, or dissemination plans of our research.

Data Sources

Between March 8 and May 28, 2019, 200 successful SP visits were conducted at 200 private Kenyan clinics. We analyze N=200 pre-demanding and N=200 post-demanding observations for the childhood diarrhea case scenario. Using Stata 16 (StataCorp, College Station, TX, USA), half the clinics were randomly assigned to receive an SP demanding albendazole, and the other half received an SP demanding amoxicillin. SP requests were done at the end of the visit or earlier if it was necessary to avoid an unusual interaction. Data was captured at two moments during the interaction: "pre-demanding" includes actions before the SP demanded the assigned medicine, and "post-demanding" includes all actions by the completion of the visit.

The scenario represents a 28-year-old mother who comes to the clinic with a 1.5-year-old child at home sick with watery diarrhea (see **Table 1**). If probed by the provider, the SP is trained to share that the child is a little hot and has passed approximately 6-7 stools in the last two days. This study follows the design and protocol from the childhood diarrhea SP case scenario described in Daniels et al. (2017).[21] In our study, the SP visits were conducted by 10 females locally recruited, trained in January 2019, and hired as SPs. All SPs were seemingly healthy, so providers would not detect and treat other health ailments that were unrelated to the SP's presenting scenario. All data reflect quality measures for SPs seeking walk-in, outpatient services. See Appendix A for additional details on SP case development, recruitment, training, and piloting.

What the SP method allows us to do is minimize bias in assessing provider practice. To assess care provided to patients in LMIC settings, the literature describes several methodologies, including direct observation, administrative or medical record abstraction, client exit interviews, provider vignettes, and standardized patients (SPs). Each method has its own interpretation and set of advantages and disadvantages which is described at length elsewhere.[8,37,38] To identify the effect of what happens when a patient demands an antibiotic or an antiparasitic medicine that is unnecessary, we randomly assigned whether the SP would demand amoxicillin or albendazole, respectively. Importantly, both medications are considered unnecessary for a child with watery diarrhea. Both medications also have harmful effects for the community if systematically and unnecessarily used. The SP method has the advantage that the researchers know the true condition of the 'patient' which is not possible when using real patients. Because of the ability for unannounced SPs to mimic an actual client-provider interaction, the SP method, which is often used for medical training in high-income countries, allows for providers across different facilities to be compared against the exact same patient scenario and is thus increasingly considered the gold standard for comparably measuring provider practice in LMICs.

To ensure accurate and comprehensive recall, within 1 to 3 hours after each SP visit, SPs completed an exit questionnaire administered by a fieldwork supervisor. The exit questionnaire

For peer review only - http://bmjopen.bmj.com/site/about/guidelines.xhtml

collected information regarding the SP's visit, including time of arrival, time of departure, history questions asked, diagnosis, lab tests ordered, medicines dispensed and prescribed, counseling given, and a subjective assessment of the visit. Further, for each visit, SPs and their supervisors attempted to identify all providers seen by the SPs. The list of providers formed the provider survey sampling frame.

Literature on quality of care in LMICs shows that large differences exist between what health care providers know and do.[39] To measure whether providers know what to do in this setting, we additionally analyze data from the provider survey conducted between November and December 2019 among providers who saw SPs. The provider survey included a vignette module to assess knowledge based on a childhood diarrhea vignette case matching the SP case scenario, in addition to capturing provider characteristics through interview.

Outcomes

Using SP and vignette data, we constructed binary measures for our main outcomes of interest: correct case management and whether any unnecessary medicines were prescribed or dispensed. Benchmarked against national guidelines, correct case management refers to the minimal and essential actions for childhood watery diarrhea case management (see Appendix Figure A1).[40] Visits were coded as being correctly managed (=1) if the provider did any one of the following: gave oral rehydration salts (ORS), advised on ORS, referred the SP, or asked the SP to return; 0, otherwise (**Table 1**). We classified ORS and zinc to be appropriate, and a provider was coded as ordering any unnecessary medicines if others were prescribed or dispensed. For our SP experiments, we examine whether the provider prescribes or dispenses the antibiotic amoxicillin or the deworming drug albendazole, which are both considered inappropriate and can be harmful at individual and community levels. Additionally, whether any antibiotic or any antiparasitic including antimalarials were assessed.

Statistical Analysis

We first conducted difference in means tests on clinic characteristics uncorrelated as a balance check that our random assignment of demanding experiments to clinics were truly random. Next, we computed adjusted odds ratios with 95% confidence intervals (CIs) from a logistic regression model, while controlling for differences that arose from our design, including a binary SP experiment variable (0 if the SP was assigned to demand albendazole; 1 if assigned to demand amoxicillin); the AHME binary treatment indicator representing whether the clinic associated with the SP-provider visit received the AHME intervention (= 1) or was assigned to the control arm (= 0), which was randomized independently from the SP experiment; and SP actor fixed effects, as illustrated in previous SP studies with similar designs. The parameter of interest is the coefficient on the SP experiment variable which is interpreted as the effect of demanding amoxicillin relative to albendazole on the outcome of interest. We complemented these analyses with ordinary least squares regression to assess differences in outcomes across the demanding experiments. Our error terms are normally distributed and clustered at the clinic-SP individual level. It is important to note that our estimates correspond with the expected average quality of care and demanding differences if the clinics were selected randomly by a patient in the country.

Analyses using SP data were conducted at the SP-provider visit level and when SP data were linked to provider survey data, the unit of observation is a successful (i.e., completed) SP-

For peer review only - http://bmjopen.bmj.com/site/about/guidelines.xhtml

provider visit with provider survey responses from the provider seen during the SP visit. All data analyses were performed with Stata 16.

Ethical Review

This study obtained IRB approval under the AHME impact evaluation study from Innovations for Poverty Action's independent IRB board, Protocol Number 1085, and from Kenya Medical Research Institute, Non-SSC Protocol Number 372. The study also received local research permission in Kenya National Commission for Science, Technology and Innovation Permit Number NA-COSTI/P/19/5343/28310. We registered our study with the American Economic Association Registry, trial number AEARCTR-0000217, and the Pan African Clinical Trial Registry, trial number PACTR201502000770329. Appendix A provides more details on (1) ethical considerations for utilizing the SP method for this study.

Results

A total of 200 SP-provider visits were successfully conducted by 10 SPs at a total of 200 different private health clinics across 34 of the 47 counties in Kenya. To ensure that the experiment was successfully randomized, we checked differences in means for the clinics assigned an SP demanding amoxicillin or albendazole. **Table 2** shows the means and 95% CIs for various characteristics. Since the groups randomly assigned to receive SPs demanding different medicines are balanced (i.e., the absolute difference between the mean value in the two groups is not different from zero), we can rely on our statistical model assumption that the randomization of demanding assignments created exchangeable treatment arms to assess the impacts of demanding different unnecessary drugs. Thus we can interpret our coefficient of interest as an unbiased estimate of the effect of demanding each medicine on our outcomes.

Table 3 provides summary statistics for the N=200 SP post-demanding observations. Just over half of the visits were conducted with a provider that appeared between 30 and 50 years of age, and about 40% of providers were female. The majority of SPs saw a medical doctor or clinical officer (0.46, 95% CI: 0.38-0.55) or a nurse or midwife (0.42, 95% CI: 0.34-0.51). On average, there were approximately 1.55 (95% CI: 1.16-1.94) individuals waiting in the waiting room when the SP arrived, and each SP visit lasted 6.99 (95% CI: 6.48-7.49) minutes with the provider, who asked on average 4.46 (95% CI: 4.08-4.83) history questions. Among the visits, 15% (95% CI: 10-20) resulted in a correct diagnosis or suspicion of watery diarrhea, and 70% (95% CI: 62-76) of the visits were correctly managed with 30% (95% CI: 23-36) of SPs asked to return and a very small percentage (3.2%, 95% CI: 1-6) referred elsewhere. Despite 70% of the SP visits being correctly managed in practice, 90% (95% CI: 85-95) of the visits had a provider who knew how to correctly manage the case as measured in the administered provider vignette. Because outcomes that were captured before demanding ("pre-demanding") cannot be entirely interpreted as a complete interaction, here we only report post-demanding measures, with pre-demanding outcomes shown in the Appendix (see Appendix Figure B2).

Effects of Demanding on Levels of Correct and Unnecessary Services

Figure 2 reports adjusted odds ratios comparing demanding albendazole versus demanding amoxicillin across various binary quality of care outcomes, controlling for the AHME evaluation

For peer review only - http://bmjopen.bmj.com/site/about/guidelines.xhtml

treatment assignment, SP individual fixed effects, and with standard errors clustered at the clinic-SP individual level. We did not find that the type of unnecessary medicine demanded had an estimated effect on correct case management or any of its components (advising on ORS, giving or advising on ORS, asking to return, or referring the patient for any reason). However, when we examined zinc, which is advised within the minimum package for facility case management as per the Kenya national guidelines because of its benefits for reducing duration and severity of episodes for watery diarrhea,[40] the adjusted odds ratio of being dispensed or prescribed zinc was 1.92 (95% CI: 0.97-3.82; p-value = 0.0613) for those who demanded amoxicillin, in relation to those who demanded albendazole. Though not statistically significant at the 5% level, this difference has a clinical significance since the lower bound of the 95% CI is very close to 1. Despite how zinc is often recommended in addition to ORS to shorten the duration of symptoms, it is not mentioned in the guidelines to be available at private health facilities. Regardless, those who demanded albendazole were 33.0% less likely to receive zinc supplementation (coefficient = -0.148, SE = 0.080, p-value = 0.066; Appendix Table B2, column 6).

With respect to inappropriate medicines, we first describe the outcomes related to antiparasitic medicines, followed by antibiotics. Demanding albendazole significantly favors the odds that albendazole is dispensed or prescribed, relative to the visits where the SP demanded amoxicillin (adjusted OR in favor of SPs demanding amoxicillin: 0.06, 95% CI: 0.02-0.23, p-value < 0.0001). This translates into a 34.8 percentage point significant increase (SE 0.060, p-value = 0.000; Appendix Table B2, column 8) in whether albendazole was given, compared to 3.1% of SPs who did not demand albendazole receiving it. This effect is similar for whether any antiparasitic is dispensed or prescribed (adjusted OR in favor of SPs demanding amoxicillin: 0.19, 95% CI: 0.08-0.48, p-value = 0.0005).

We find higher rates of any antibiotic being given relative to any antiparasitic being given (58%, 95% CI: 51-65 and 26%, 95% CI: 20-30, respectively; Table 3) for all the visits resulted in any medicine prescribed. For all visits regardless of what the SP demanded, the most frequently given antibiotics were metronidazole (N = 54, 27%), sulfamethoxazole and trimethoprim (N=38, 19%, metronidazole benzoate (N=24, 12%), and amoxicillin (N=19, 10%). We find evidence that demanding amoxicillin has no effect on whether providers dispense or prescribe it (adjusted OR: 1.73, 95% CI: 0.50-5.98) with a similar null finding on whether providers dispense or prescribe any antibiotic (adjusted OR: 1.18, 95% CI: 0.58-2.41) relative to the visits with SPs demanding albendazole. Demanding albendazole versus amoxicillin resulted in different types of medicines being dispensed or prescribed at different frequencies across the SP visits. In Appendix Table B3, we present the medicines given by SP experiment.

Discussion

Using the SP method, this study reports the extent to which provider treatment behaviors are influenced by patient demand for treatment, particularly two medicines that are unnecessary, harmful to the community, yet provided as empiric treatment for acute childhood watery diarrhea. We compared the impact of a patient demanding an antibiotic medicine (amoxicillin, which has known public health risks for the individual and community), to an antiparasitic medication (albendazole) which is perceived to be harmless to the individual but also poses a risk to the community.

For peer review only - http://bmjopen.bmj.com/site/about/guidelines.xhtml

Our findings do suggest that providers who receive a client demanding amoxicillin are less likely to dispense what is demanded than when a patient demands albendazole. Nonetheless, it is worth noting that – irrespective of patient demanding – 58% (95% CI: 51-65) of the total 200 SP-provider visits carried out in our study were given or prescribed antibiotics, which is consistent with estimates from private facilities in other settings such as India and Tanzania.(23,24,34) However, this proportion is higher than observed in the public sector in Kenya, as observed in a smaller cross-sectional SP study carried out in purposively sampled health facilities in Nairobi, where 32.5% (95% CI: 20.0-47.5) of 40 SP-provider visits for child diarrhea led to antibiotic prescribing.[21,23] Similar to other observations from African countries including Kenya, top prescribed antibiotics were from the WHO Access group, such as amoxicillin and metronidazole, partly reflecting the lower cost and easier access compared to other antibiotics.

Providers may be trading off clinical benefits and risks with profits, but doing so based on how concrete clinical consequences are with respect to what may be more appropriate for the presenting condition. We confirmed this through anecdotal narratives during debriefs with the supervisors of the SP fieldwork:

- ***SP demanding amoxicillin:*** *The provider said that amoxicillin is an antibiotic, and he is not allowed to prescribe an antibiotic for diarrhea. He showed the SP a book called "Managing Diarrhea" and showed her somewhere written that a provider is only supposed to prescribe ORS and zinc for diarrhea cases.*

- ***SP demanding albendazole:*** *The SP went to the facility and explained her case, and the provider gave her ORS and zinc and advised her that if she noticed some symptoms of severe dehydration in the child, such as sunken eyes or fontanelle, or if the skin does not pull back immediately when the baby is pinched, that she should take her to the hospital immediately. When the SP demanded albendazole, the provider gave her an albendazole tablet and told her to go divide it into half and give it to the child.*

- ***SP demanding amoxicillin:*** *After demanding amoxicillin, the provider stared for a while at the mother. She said that amoxicillin is never a good option for a child with diarrhea and insisted that paracetamol would work very well. She also dispensed a dewormer wrapped in a syringe and advised to use the same syringe when administering other medicines dispensed to the child.*

This suggests that prescribing behaviors in response to patients demanding unnecessary medicines is driven at least in part by the training of patient-facing private sector providers across Kenya. A future study could examine this more in depth. Other factors likely play a role in determining prescribing practices, including the limited access to diagnostics to rule out conditions that do not require antimicrobial treatment. Further, we caution on extrapolation to other settings where knowledge and training may not be as high, since knowledge on other correct management outcomes appear to be higher in Kenya than in other LMIC settings for both infectious conditions as well as non-communicable diseases.[21,39,41]

In this study, we did not categorize the efficacy or safety of these drugs, since classifying the prescription of the medicines that were demanded in this study as "harmful" may be misleading or lead to misinterpretation. The safety profile of both drugs in terms of side effects is very good, which is reassuring. Both amoxicillin and albendazole are well tolerated even in young children.

For peer review only - http://bmjopen.bmj.com/site/about/guidelines.xhtml

However, this might provide the false perception of harmlessness which favors the inappropriate use. In this specific context, the threats to public health likely are much greater than those to the individual. The inappropriate use of amoxicillin, though narrow-spectrum and less problematic than other antibiotics, could favor resistance selection amongst commensal and pathogenic bacteria. Similar considerations apply to albendazole, though the consequences of its widespread use are less studied especially in human medicine.

This study adds to the literature in several ways. First, we extend the research done on the roles of patients and providers in the patient-provider relationship, in particular, what happens when patients demand inappropriate care and to what extent do demanding patients have an influence on services? Most notably, our findings stand in contrast with Currie et al. (2014)'s SP study in China, which found that providers increased antibiotic use from 55% to 85% when SPs requested antibiotics. Instead we found that demanding an inappropriate antibiotic did not increase its use, but demanding an antiparasitic did.[7] However, our findings complement what was reported by Lopez et al. (2020), who comparing both provider and patient roles, assessed whether patients' demands influence overprescription of antibiotics in Mali.[42] With a large sample of real patients randomly assigned different information and malaria treatment subsidies across 60 health facilities in Bamako, the authors found that patients demanding resulted in higher rates of treatment than if providers were in control of dispensing vouchers. They additionally found that for more severe cases, providers were reluctant to provide inappropriate treatment, but that patient driven demand resulted in an excess of treatment for milder cases. We also add to the literature on overdispensing of antimicrobial therapy and understanding quality of care outcomes that are related to antimicrobial resistance. This study has implications for antimicrobial stewardship efforts and quality improvement interventions.

Our study is not without limitations. First, the SP method requires a one-time visit for services that (1) do not subject the client to invasive procedures; (2) can only assess tracer health conditions that have been validated for ethical research; and (3) do not require established client services or follow-up visits, such as those related to chronic conditions or other ailments. However, we identified the method's limitations as favorable conditions to assess the quality of walk-in outpatient services.

Second, we cannot compare levels of services without demanding, because by design, the SPs demanded medicines at the end of the visit, though on some occasions the SPs had to demand earlier (e.g., when the provider was discussing treatment or sending the SP to the clinic's pharmacy) to ensure that there was a consistent though standardized narrative for the visit. A future study that seeks to compare demanding outcomes to not demanding would have to implement separate SP visits.

Third, given that we only examine the interaction between providers and SPs, it is outside the scope of this study to report on the role of care-seeking behavior and thus interpret findings conditional on patients seeking care. However, because of the standardized presentation of the childhood diarrhea case scenario used in this study, we are able to know what happens across multiple providers when they are presented with the same case.

For this reason, we cannot interpret our pre-demanding observations as care provided to an SP who did not demand any medicines. Given this limitation, we have scrutinized a few possible channels in our data that are related to either provider behavior or limitations of the method we

For peer review only - http://bmjopen.bmj.com/site/about/guidelines.xhtml

implemented. Based on the study design, the increase in correct case management for watery diarrhea can only be related to variables where we have captured the outcome at two time points: pre- and post-demanding. Thus, effects of demanding on correct case management is related to advising on ORS or asked to return. It cannot be from dispensing/prescribing ORS or referring to another facility, which are both captured once at the end of the visit. One can imagine that having two time points alerts us to an issue that the post-demanding environment simply captures more dispensing/prescribing of ORS, and thus higher correct care because it captures all actions after the entire visit has been completed.

Conclusion

In the setting of private primary care in Kenya, advantages of the SP method are that we can assess providers with the same patient presentation and can causally infer the effects of patient characteristics and actions on quality of care, because the patient characteristics are by design and can be randomly assigned to providers. Most notably, we sought to investigate whether explicitly asking for amoxicillin (an unnecessary antibiotic) or albendazole (an unnecessary antiparasitic used for deworming) had an impact on correct case management and drug prescribing. That we found that providers increase the misuse of the antiparisitic but not the amoxicillin suggests the need for future research on provider knowledge and perceptions of profit and individual and community health trade-offs when making prescription decisions after patients demand specific incorrect care.

Competing interests The authors do not have any competing interests to disclose.

Funding This project was funded by a grant from the Bill and Melinda Gates Foundation (Opportunity ID#: OPP1044138). The former UK Department of International Development provided funding for the grant.

Acknowledgments The authors would like to thank Nicole Perales, Rita Cuckovich, Afke Jagar and Innovations for Poverty Action Kenya for their technical and research assistance; to Jishnu Das and Amy Dolinger for their support; Stefano Bertozzi, Jonathan Kolstad, Aprajit Mahajan for their comments and feedback; Catherine Wanjiru, John Wanangwe, Jacob Odipo, Daniel Waithaka for their advising; to Andrew Muriithi, Pheliciah Mwachofi, Purity Kimuru, Rodgers Kegode, and Salome Omondi for their superb supervision; and to the SPs for their communication and hard work.

Author Contributions Conceptualization: AK, CB, GS, PG. Funding acquisition: PG, AK. Methodology: AK, PG. Fieldwork supervision: AK. Coding of medicines and tests: AK, GS. Writing ± original draft: AK, CB, GS. Writing ± review & editing: AK, GS, CB, PG.

Data Sharing Statement Individual de-identified interaction data, including data dictionaries, will be available. All variables needed to re-create the results reported in this article will be included, as will the code required to reproduce these results. Data will be available indefinitely

For peer review only

upon publication to anyone who wishes to access the data for any purpose. The data and code can be accessed at https://github.com/kwantify.

For peer review only

For peer review only - http://bmjopen.bmj.com/site/about/guidelines.xhtml

References

1. Zanichelli V, Tebano G, Gyssens I, Vlahović-Palčevski V, Monnier A, Benic MS, et al. Patient-related determinants of antibiotic use: A systematic review. Clinical microbiology and infection. 2019;25(1):48–53.

2. Coenen S, Francis N, Kelly M, Hood K, Nuttall J, Little P, et al. Are patient views about antibiotics related to clinician perceptions, management and outcome? A multi-country study in outpatients with acute cough. PloS one. 2013;8(10):e76691.

3. Wagner CE, Prentice JA, Saad-Roy CM, Yang L, Grenfell BT, Levin SA, et al. Economic and behavioral influencers of vaccination and antimicrobial use. Frontiers in Public Health. 2020;8:975.

4. Rodrigues AT, Roque F, Falcão A, Figueiras A, Herdeiro MT. Understanding physician antibiotic prescribing behaviour: A systematic review of qualitative studies. International journal of antimicrobial agents. 2013;41(3):203–12.

5. Chen BK, Gertler PJ, Yang C-Y. Physician ownership of complementary medical services. Journal of Public Economics. 2016;144:27–39.

6. Currie J, Lin W, Zhang W. Patient knowledge and antibiotic abuse: Evidence from an audit study in china. Journal of health economics. 2011;30(5):933–49.

7. Currie J, Lin W, Meng J. Addressing antibiotic abuse in china: An experimental audit study. Journal of development economics. 2014;110:39–51.

8. Kwan A, Daniels B, Bergkvist S, Das V, Pai M, Das J. Use of standardised patients for healthcare quality research in low-and middle-income countries. BMJ global health. 2019;4(5):e001669.

9. Bell M. Antibiotic misuse: A global crisis. JAMA internal medicine. 2014;174(12):1920–1.

10. Wang J, Wang P, Wang X, Zheng Y, Xiao Y. Use and prescription of antibiotics in primary health care settings in china. JAMA internal medicine. 2014;174(12):1914–20.

11. World Health Organization. Antimicrobial resistance [Internet]. 2020 [cited 2020 Oct 13]. Available from: https://www.who.int/news-room/fact-sheets/detail/antimicrobial-resistance

12. Agarwal S, Yewale VN, Dharmapalan D. Antibiotics use and misuse in children: A knowledge, attitude and practice survey of parents in india. Journal of clinical and diagnostic research: JCDR. 2015;9(11):SC21.

13. Labi A-K, Obeng-Nkrumah N, Bjerrum S, Aryee NAA, Ofori-Adjei YA, Yawson AE, et al. Physicians' knowledge, attitudes, and perceptions concerning antibiotic resistance: A survey in a ghanaian tertiary care hospital. BMC health services research. 2018;18(1):1–2.

For peer review only

For peer review only - http://bmjopen.bmj.com/site/about/guidelines.xhtml

14. Adhikari B, Pokharel S, Raut S, Adhikari J, Thapa S, Paudel K, et al. Why do people purchase antibiotics over-the-counter? A qualitative study with patients, clinicians and dispensers in central, eastern and western nepal. BMJ global health. 2021;6(5):e005829.

15. Greene J, Hibbard JH. Why does patient activation matter? An examination of the relationships between patient activation and health-related outcomes. Journal of general internal medicine. 2012;27(5):520–6.

16. Hibbard JH, Stockard J, Mahoney ER, Tusler M. Development of the patient activation measure (PAM): Conceptualizing and measuring activation in patients and consumers. Health services research. 2004;39(4p1):1005–26.

17. Hibbard JH, Greene J. What the evidence shows about patient activation: Better health outcomes and care experiences; fewer data on costs. Health affairs. 2013;32(2):207–14.

18. Mohanan M, Vera-Hernández M, Das V, Giardili S, Goldhaber-Fiebert JD, Rabin TL, et al. The know-do gap in quality of health care for childhood diarrhea and pneumonia in rural india. JAMA pediatrics. 2015;169(4):349–57.

19. Das J, Holla A, Das V, Mohanan M, Tabak D, Chan B. In urban and rural india, a standardized patient study showed low levels of provider training and huge quality gaps. Health affairs. 2012;31(12):2774–84.

20. Sylvia S, Shi Y, Xue H, Tian X, Wang H, Liu Q, et al. Survey using incognito standardized patients shows poor quality care in china's rural clinics. Health policy and planning. 2014;30(3):322–33.

21. Daniels B, Dolinger A, Bedoya G, Rogo K, Goicoechea A, Coarasa J, et al. Use of standardised patients to assess quality of healthcare in nairobi, kenya: A pilot, cross-sectional study with international comparisons. BMJ global health. 2017;2(2):e000333.

22. Tielsch JM. The know, do, and quality gaps in international maternal and child health care interventions. JAMA pediatrics. 2015;169(4):313–4.

23. Sulis G, Daniels B, Kwan A, Gandra S, Daftary A, Das J, et al. Antibiotic overuse in the primary health care setting: A secondary data analysis of standardised patient studies from india, china and kenya. BMJ global health. 2020;5(9):e003393.

24. Sulis G, Adam P, Nafade V, Gore G, Daniels B, Daftary A, et al. Antibiotic prescription practices in primary care in low-and middle-income countries: A systematic review and meta-analysis. PLoS medicine. 2020;17(6):e1003139.

25. Carter E, Bryce J, Perin J, Newby H. Harmful practices in the management of childhood diarrhea in low-and middle-income countries: A systematic review. BMC Public Health. 2015;15(1):1–34.

26. Fink G, D'Acremont V, Leslie HH, Cohen J. Antibiotic exposure among children younger than 5 years in low-income and middle-income countries: A cross-sectional study of nationally representative facility-based and household-based surveys. The Lancet infectious diseases. 2020;20(2):179–87.

For peer review only

27. Doan T, Worden L, Hinterwirth A, Arzika AM, Maliki R, Abdou A, et al. Macrolide and nonmacrolide resistance with mass azithromycin distribution. New England Journal of Medicine. 2020;383(20):1941–50.

28. Rhee C, Aol G, Ouma A, Audi A, Muema S, Auko J, et al. Inappropriate use of antibiotics for childhood diarrhea case management—kenya, 2009–2016. BMC Public Health. 2019;19(3):1–2.

29. Troeger C, Forouzanfar M, Rao PC, Khalil I, Brown A, Reiner Jr RC, et al. Estimates of global, regional, and national morbidity, mortality, and aetiologies of diarrhoeal diseases: A systematic analysis for the global burden of disease study 2015. The Lancet Infectious Diseases. 2017;17(9):909–48.

30. Walker CLF, Rudan I, Liu L, Nair H, Theodoratou E, Bhutta ZA, et al. Global burden of childhood pneumonia and diarrhoea. The Lancet. 2013;381(9875):1405–16.

31. World Health Organization. Diarrhoeal disease - key facts [Internet]. 2017 [cited 2017 May 2]. Available from: https://www.who.int/news-room/fact-sheets/detail/diarrhoeal-disease

32. Mokomane M, Kasvosve I, Melo E de, Pernica JM, Goldfarb DM. The global problem of childhood diarrhoeal diseases: Emerging strategies in prevention and management. Therapeutic advances in infectious disease. 2018;5(1):29–43.

33. World Health Organization Department of Child and Adolescent Health and UNICEF. Handbook IMCI: Integrated management of childhood illness. World Health Organization; 2005.

34. King JJ, Powell-Jackson T, Makungu C, Hargreaves J, Goodman C. How much healthcare is wasted? A cross-sectional study of outpatient overprovision in private-for-profit and faith-based health facilities in tanzania. Health policy and planning. 2021;36(5):695–706.

35. Flottorp SA, Oxman AD, Krause J, Musila NR, Wensing M, Godycki-Cwirko M, et al. A checklist for identifying determinants of practice: A systematic review and synthesis of frameworks and taxonomies of factors that prevent or enable improvements in healthcare professional practice. Implementation Science. 2013;8(1):1–1.

36. World Health Organization. Standards for improving the quality of care for children and young adolescents in health facilities. 2018;

37. Peabody JW, Luck J, Glassman P, Dresselhaus TR, Lee M. Comparison of vignettes, standardized patients, and chart abstraction: A prospective validation study of 3 methods for measuring quality. Jama. 2000;283(13):1715–22.

38. Das J, Hammer J, Leonard K. The quality of medical advice in low-income countries. Journal of Economic Perspectives. 2008;22(2):93–114.

39. Das J, Woskie L, Rajbhandari R, Abbasi K, Jha A. Rethinking assumptions about delivery of healthcare: Implications for universal health coverage. BMJ. 2018;361:k1716.

40. Kenya Ministry of Health. Policy guidelines for management of diarrhoea in children below five years in kenya. Kenya Ministry of Health; 2014.

For peer review only

For peer review only - http://bmjopen.bmj.com/site/about/guidelines.xhtml

41. Di Giorgio L, Evans DK, Lindelow M, Nguyen SN, Svensson J, Wane W, et al. Analysis of clinical knowledge, absenteeism and availability of resources for maternal and child health: A cross-sectional quality of care study in 10 african countries. BMJ global health. 2020;5(12):e003377.

42. Lopez C, Sautmann A, Schaner S. Does patient demand contribute to the overuse of prescription drugs? 2020;

For peer review only

Figure 1. Clinic sample and SP randomized study design

For peer review only

For peer review only - http://bmjopen.bmj.com/site/about/guidelines.xhtml

Table 1. Description of childhood diarrhea standardized patient case scenario and main outcomes

**Case**	**Case description**	**SP experiments varying patient characteristics**	**Outcomes**
Childhood diarrhea	A 28-year-old mother comes to the clinic with a 1.5-year-old child at home sick with acute watery diarrhea. The child is a little hot and has passed approximately 6-7 stools in the last two days.	Experiment 1: Demanding albendazole<br><br>Experiment 2: Demanding amoxicillin	Correct case management (=1): Any one of the following were done by the provider:<br>gave ORS, advised on ORS,<br>referred elsewhere,<br>asked to return to clinic for any reason<br><br>Any unnecessary medicines (=1): Any medicines given excluding ORS, zinc

For peer review only - http://bmjopen.bmj.com/site/about/guidelines.xhtml

Table 2. Balance across characteristics of clinics assigned albendazole vs. amoxicillin demanding experiment.

	Clinics Assigned to Receive and SP Demanding Albendazole			Clinics Assigned to Receive an SP Demanding Amoxicillin			
	N	Mean	95% CI	N	Mean	95% CI	*p*-value
Number of Patients (Last Week)	96	97.76	(79.37 - 116.15)	89	87.40	(73.00 - 101.81)	0.391
Years of Ownership	96	9.33	(7.78 - 10.88)	89	10.47	(8.78 - 12.17)	0.332
Has Laboratory	96	0.80	(0.72 - 0.88)	89	0.78	(0.69 - 0.86)	0.657
Offers Inpatient Services	96	0.21	(0.13 - 0.29)	89	0.21	(0.13 - 0.30)	0.932
Has NHIF	96	0.11	(0.05 - 0.18)	89	0.15	(0.07 - 0.22)	0.527
Share of Clients are Family Planning	78	0.16	(0.14 - 0.19)	75	0.18	(0.15 - 0.21)	0.382
Keeps Records for Revenues	96	0.77	(0.69 - 0.86)	89	0.69	(0.59 - 0.78)	0.193
Keeps Records for Expenses	96	0.72	(0.63 - 0.81)	89	0.65	(0.55 - 0.75)	0.329
Licensed	91	0.94	(0.90 - 0.98)	85	0.89	(0.83 - 0.94)	0.122
Average Monthly Revenue (KSh)	94	2424.75	(1,535.60 - 3,313.91)	87	1764.72	(1,396.25 - 2,133.19)	0.193
Average Monthly Expenditures (KSh)	94	1536.41	(783.63 - 2,289.18)	88	1073.71	(837.25 - 1,310.18)	0.265
Total Revenue (Last Month, KSh)	94	2067.63	(1,459.09 - 2,676.17)	87	1675.41	(1,343.51 - 2,007.31)	0.279
Total Expenditures (Last Month, KSh)	93	1167.10	(782.15 - 1,552.05)	87	1082.23	(828.49 - 1,335.97)	0.722

Note. Number of observations refers to the number of clinics in the sample visited by SPs. The data source for this table does not have data available for all 200 private clinics in the sample. NHIF = National Hospital Insurance Fund. KSh = Kenya Shillings (approximately 100 KSh = 1 United States Dollar).

view only

Table 3. Summary statistics of SP visits

	(1) Pooled SP Visits			(2) SP Visits Assigned to Demand Albendazole			(3) SP Visits Assigned to Demand Amoxicillin			(3)-(2)
	N	Mean	95% CI	N	Mean	95% CI	N	Mean	95% CI	*p*-value
**Provider characteristics**										
Provider is female	146	0.37	(0.29 - 0.45)	75	0.33	(0.23 - 0.44)	71	0.41	(0.29 - 0.52)	0.351
Provider age group	189			95			94			
Under 30	33	0.17	(0.13 - 0.24)	18	0.19	(0.12 - 0.28)	15	0.16	(0.10 - 0.25)	
Between 30 and 50	114	0.60	(0.53 - 0.67)	59	0.62	(0.52 - 0.71)	55	0.59	(0.48 - 0.68)	
Above 50	42	0.22	(0.17 - 0.29)	18	0.19	(0.12 - 0.28)	24	0.26	(0.18 - 0.35)	
Provider qualification	142			75			67			
Medical doctor or Clinical Officer	66	0.46	(0.38 - 0.55)	36	0.48	(0.37 - 0.59)	30	0.45	(0.33 - 0.57)	
Nurse or Midwife	60	0.42	(0.34 - 0.51)	31	0.41	(0.31 - 0.53)	29	0.43	(0.32 - 0.55)	
Other Staff	16	0.11	(0.07 - 0.18)	8	0.11	(0.05 - 0.20)	8	0.12	(0.06 - 0.22)	
Knowledge of correct management										
Diarrhea	140	0.90	(0.85 - 0.95)	72	0.92	(0.85 - 0.98)	68	0.88	(0.81 - 0.96)	0.502
**Visit characteristics**										
Number of patients waiting	200	1.55	(1.16 - 1.94)	102	1.25	(0.82 - 1.68)	98	1.87	(1.20 - 2.53)	0.122
Minutes spent with provider	189	6.99	(6.48 - 7.49)	95	6.61	(5.98 - 7.24)	94	7.37	(6.59 - 8.16)	0.140
Number of history questions asked (post)	200	4.46	(4.08 - 4.83)	102	4.41	(3.86 - 4.97)	98	4.50	(3.98 - 5.02)	0.820
Correct diagnosis or suspicion (post)	200	0.15	(0.10 - 0.20)	102	0.11	(0.05 - 0.17)	98	0.19	(0.12 - 0.27)	0.089
Correct case management (post)	200	0.70	(0.63 - 0.76)	102	0.67	(0.58 - 0.76)	98	0.72	(0.64 - 0.81)	0.377
Any lab tests ordered (post)	200	0.13	(0.08 - 0.18)	102	0.16	(0.09 - 0.23)	98	0.10	(0.04 - 0.16)	0.251
Total lab tests ordered (post)	200	0.26	(0.16 - 0.35)	102	0.29	(0.16 - 0.43)	98	0.21	(0.08 - 0.35)	0.408
Any unnecessary lab tests (post)	200	0.10	(0.06 - 0.14)	102	0.08	(0.03 - 0.13)	98	0.12	(0.06 - 0.19)	0.302
Total unnecessary lab tests (post)	189	0.15	(0.09 - 0.20)	95	0.17	(0.09 - 0.24)	94	0.13	(0.05 - 0.21)	0.468
Number of medicines	190	2.50	(2.22 - 2.78)	101	2.43	(2.04 - 2.82)	89	2.58	(2.19 - 2.98)	0.577
Number of non-efficacious medicines	190	1.71	(1.49 - 1.93)	101	1.76	(1.45 - 2.08)	89	1.65	(1.35 - 1.95)	0.619
Dispensed/ prescribed: Albendazole	190	0.20	(0.14 - 0.26)	101	0.35	(0.25 - 0.44)	89	0.03	(0.00 - 0.07)	0.000
Dispensed/ prescribed: Any antiparasitic	190	0.26	(0.20 - 0.32)	101	0.36	(0.26 - 0.45)	89	0.15	(0.07 - 0.22)	0.001
Dispensed/ prescribed: Amoxicillin	190	0.10	(0.06 - 0.14)	101	0.08	(0.03 - 0.13)	89	0.12	(0.06 - 0.19)	0.311
Dispensed/ prescribed: Any antibiotic	190	0.58	(0.51 - 0.65)	101	0.56	(0.47 - 0.66)	89	0.61	(0.51 - 0.71)	0.557

Asked to return (post)	200	0.30	(0.23 - 0.36)	102	0.30	(0.21 - 0.39)	98	0.29	(0.20 - 0.38)	0.779
Referred elsewhere	189	0.03	(0.01 - 0.06)	95	0.03	(0.00 - 0.07)	94	0.03	(0.00 - 0.07)	0.990
Providers did good job explaining	189	0.76	(0.70 - 0.82)	95	0.74	(0.65 - 0.83)	94	0.79	(0.70 - 0.87)	0.419

Note. Table displays summary statistics are for all SP visits pooled (column 1), all SP visits assigned to demand albendazole (column 2), and all SP visits assigned to demand amoxicillin (column 3). Statistics with "(post)" are post-demanding measures; all others are one time at the end of the visit. All summary statistics except knowledge of correct management for diarrhea come from SP surveys. Knowledge of correct management is defined in the same way as correct case management and come from a vignette administered in the provider survey. Vignette data are matched to SP data for each SP visit by provider seen by SP or a replacement for the sampled provider. Single observations for each provider in the provider survey may occur multiple times if SPs saw that provider multiple times. Provider age group is the estimated age group as perceived by the SP.).

For peer review only

Figure 2. Differences in quality of care by standardized patients demanding albendazole vs. amoxicillin

Note. The chart illustrates estimated differences by the SP demanding experiment across quality of care outcomes. Odds ratios are estimated controlling for SP fixed effects and standard errors are clustered at the clinic and individual standardized patient levels. All variables are binary outcomes.

For peer review only

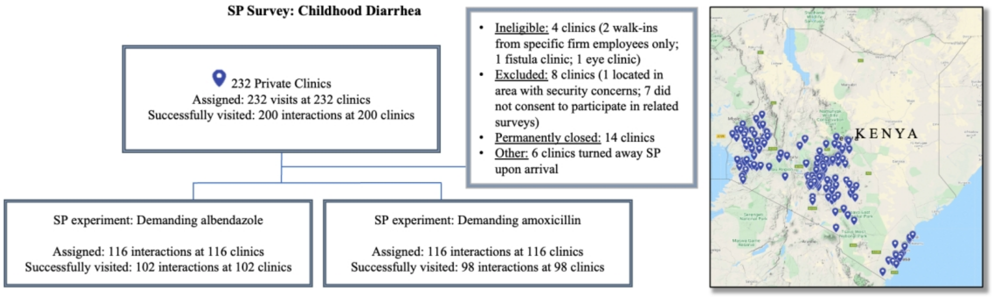

Figure 1. Clinic sample and SP randomized study design

593x191mm (72 x 72 DPI)

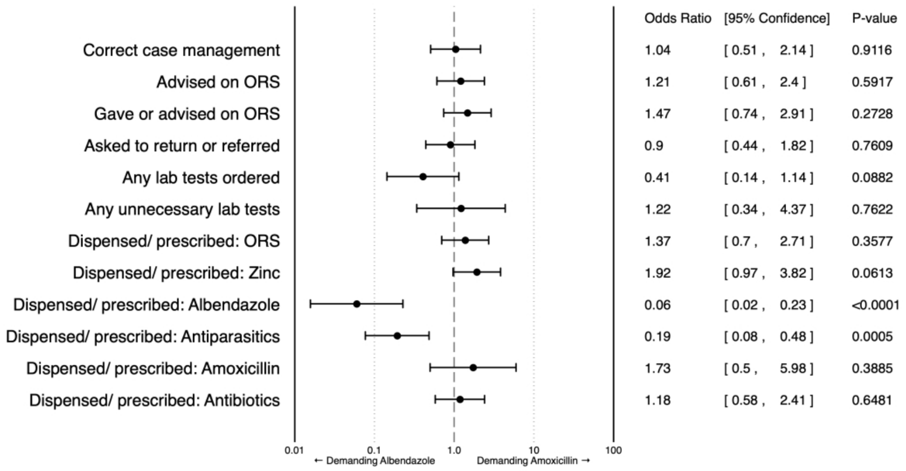

Figure 2. Differences in quality of care by standardized patients demanding albendazole vs. amoxicillin

Note. The chart illustrates estimated differences by the SP demanding experiment across quality of care outcomes. Odds ratios are estimated controlling for SP fixed effects and standard errors are clustered at the clinic and individual standardized patient levels. All variables are binary outcomes.

527x263mm (144 x 144 DPI)

SUPPLEMENTAL APPENDICES

Supplement to:

Do private providers give patients what they demand, even if it is inappropriate? A randomized study utilizing standardized patients in Kenya

Ada Kwan[1,2], Claire E. Boone[2], Giorgia Sulis[3], and Paul J. Gertler[4]

[1]Division of Pulmonary and Critical Care Medicine, University of California San Francisco School of Medicine, San Francisco, CA, United States
[2]Division of Health Policy and Management, University of California, Berkeley School of Public Health, Berkeley, CA, United States
[3]Department of Epidemiology, Biostatistics, and Occupational Health, McGill University, Montreal, Canada
[4]Haas School of Business, University of California, Berkeley, Berkeley, CA, United States

For peer review only

For peer review only - http://bmjopen.bmj.com/site/about/guidelines.xhtml

APPENDIX A: SUPPLEMENTAL METHODS

A.1 Study Context, SP Method, and Ethics

For peer review only

For peer review only - http://bmjopen.bmj.com/site/about/guidelines.xhtml

A.1.1 Childhood diarrhea case management

Appendix Figure A1. Kenya national guidelines for health facility case management of childhood diarrhea

Who

- Health care providers in public, private, and faith-based facilities should implement outlined Ministry of Health guidelines in management of diarrhea in chidlren under five years old.
- All healthcare providers including doctors, nurses, clinical officers, nutritionists, pharmacists, and public health offiers should be trained on proper case management in children under five years.

Minimum package for facility case management

- All facilities should have a functional ORT corner for immediate start of on-site rehydration of children with diarrhea.
- All children with diarrhea should be given ORS and zinc combination therapy as soon as diarrhea has started. Combination therapy of ORS and zinc tablets (Co-pack) is available at the following places: public health facilities, comunity health units, pharmacies (public / private), and retail shops.

Product information

- ORS: benefits include reduction of stool output, reduction of vomiting incidence, replacement of sodium bicarbonate and potassium, replacement of admission burden and IV fluid treatment. Low osmolarity ORS; local production and procurement; half litre sachet.
- Zinc: benefits include reduction of duration and severity of episodes, lowering of diarrhea incidence in the following 2-3 months, reduction of pneumonia incidence, improvement of cellular intestinal immune and epithelial functioning, and increased functioning of the immune system. Dispersible tablets (20 mg) with local production and procurement.

Caregiver responsibilities

- On leaving a health facility for home, a caregiver shall be given one co-pack containing 4 sachets of ORS and 10 tablets of zinc and will receive guidance on home management.
- In addition, the caregiver should be advised to return to the health facility within two days if diarrhea continues or immediately if the child: is not able to drink or breastfeed, becomes sicker, is vomiting everything, is drinking poorly, or has blood in stool.

Other drugs should be given only when deemed absolutely necessary during the management of diarrheal diseases. General guidelines are:

- Guidelines also state that all children with diarrhea shall be given an appropriate dose of Vitamin A at the facility if they have not received a dose in the last one month. IV should be used in children with the following conditions: severe dehydration (as per Integrated Management of Childhood Illnesses guidelines); severe, profuse, repeated diarrhea; persistent vomiting; inability to drink; abdominal distention; paralytic ileus; glucose malabsorption; shock. The fluid of choice shall be Ringer's lactate. In cases where it is not available, normal saline can be used. Every effort should be made to replace IV therapy with oral therapy as soon as the child is able to drink. Where the child cannot get IV therapy at once, ORS solution shall be given by nasogastric tube or orally until IV therapy is started.
- Management of dehydration in children with severe malnutrition: the fluid of choice is rehydration solution for malnutrition. IV fluids should not be used unless the child is in shock.
- Antidiarrheal and anti-emetics shall not be used. None have any proven practical value and some are dangerous.
- Antibiotics shall be used only for supsected or proven dysentery and cholera cases. In diarrhea of any other aetiology anitbiotics are of no practical value and shall not be given.
- Antiprotozoal drugs shall only be used for the treatment of amoebiasis and giardiasis.
- For cholera, erythromycin (1st line) and chloramphenical (2nd line). For shigella dysentery, ciprofloxacin. For amoebiasis, metronidazole. For giardiasis, metronidazole

Drugs, equipment, and supplies

- A commodity management system, which enhances visibility of procured drugs, equipment and supplies, should be promoted to strengthen the availability of diarrhea management commodities. The following drugs, equipment and supplies should be available at all times.
- Drugs: zinc and ORS, erythromycin, chloramphenicol, ciprofloxacin, Vitamin A, metronidazole, and intravous lfuids such as Ringer's lactate, normal saline and Resomal. Oral rehydration equipment and supplies as per the ORT corner guidelines.

A.1.2 African Health Markets for Equity (AHME) Program and Evaluation

Details of the AHME intervention are provided where necessary for this study; however, the impact of the AHME intervention is not the purpose of this study. Across Kenya between 2013-2018, the AHME intervention was intended to "use National Health Insurance to link supply (private providers) with demand (clients) in order to shift health markets toward providing quality health care to low-income patients" . In Kenya, 56% of the population earns US$1 or less a day, and a third of the poor who are sick do not seek care, according to National Health Accounts data. To improve this situation, the AHME intervention is a mix of demand- and supply-side interventions implemented by non-governmental organizations in the context of a new scheme within the National Health Insurance Fund (NHIF). The demand- and supply-side interventions include: National Social Health Insurance scheme by Ministry of Health; SafeCare quality improvement by PharmAccess (similar to that described in Dunsch et al. [12]); Social franchising by Marie Stopes Kenya (MSK) and Population Services Kenya (PSK). The AHME impact evaluation's main aim is to assess the impacts and cost-effectiveness of the AHME intervention. Additionally, the evaluation aims to assess AHME's impact on several dimensions of quality including: (1) SafeCare's 680 measures of quality used in health facility assessments and quality improvement plans, (2) perceived quality measured by previous patients through a household survey, (3) perceived quality by existing patients through exit interviews, (4) provider knowledge through provider vignettes, (5) provider practice through SPs, and (6) patient safety outcomes through direct observation techniques and SPs.

A.1.2.1. Experimental Design of the AHME Impact Evaluation

This section describes the AHME impact evaluation experimental design and clinic selection. Figure 1 shows a map of the clinics across Kenya alongside the process of clinic selection, which is described in detail as six steps below.

<u>Step 1. Clinics in Kenya listed.</u> Before the AHME program began in 2012, we mapped all clinics in 35 of Kenya's 47 counties with the goal of randomizing clinics eligible for the AHME program into treatment and control groups. Because there was no pre-existing list of private clinics at the time, clinics were first identified for mapping using four sources of information: (1) official government list of private clinics in the country, (2) clinics belonging to a major professional health associate (e.g., the Kenya Nurse and Midwives Association), (3) clinics that the AHME implementation partners suggested should be visited, and (4) additional clinics identified by evaluation teams in the field during the mapping process, but not included on any of the above lists. Government clinics (public clinics and hospitals), faith-based clinics (identified by clinic name), and clinics that were identified as franchised (by franchise branding on clinic exterior) were removed from the sampling frame generated from the aforementioned information sources.

<u>Step 2. Baseline clinic survey administered.</u> A pre-screening and baseline survey among remaining clinics was administered to remaining clinics. The purpose of this was to exclude clinics that the implementing partners indicated were not eligible for franchise services or the AHME set of interventions. Clinics that met the basic eligibility criteria still varied in their "level of eligibility" based on their existing capacity and suitability for franchising services and AHME interventions. Using additional criteria, created in a collaborative manner with MSK and PSK, clinics were further categorized into groups based on how likely they were to be eligible ("eligibility tiers") using data collected through the baseline survey instrument.

<u>Step 3. Randomization.</u> After clinics were categorized by eligibility tier, the research team conducted a stratified randomization of eligible clinics. For the randomization process, clinics were grouped into their

For peer review only - http://bmjopen.bmj.com/site/about/guidelines.xhtml

eligibility tiers within a county based on partner-provided criteria and data from the baseline survey, randomly ordered within strata (groups within which randomization would occur), and then randomly assigned to treatment (eligible to be offered AHME franchising immediately) or control (not eligible to be offered AHME until the completion of the study).

Steps 4 & 5. Clinics screened based on survey & site visit screening. After randomization procedures were completed, we provided MSK and PSK with partner-specific recruitment lists indicating the order in which clinics on their lists were to be approached for screening ("sensitization") and recruitment. Once randomization procedures had been completed, MSK and PSK began engaging clinics on their lists and proceeded with their respective recruitment procedures in October 2013. The second round of screening and recruitment by the partners served to identify clinics that were eligible for franchising and AHME interventions in the treatment arm. Eligible clinics that were invited to join either franchise ("ever franchised") were considered part of the evaluation sample in the treatment arm. Consistent with the intent-to-treat (ITT) assumptions applied in this study, these ever-franchised clinics were considered part of the final evaluation sample regardless of whether they completed the franchise enrollment process or maintained their franchise enrollment status for the entirety of the study period (for any reason).

Step 6. Final clinic sample for AHME impact evaluation. The final AHME evaluation samples were identified over the course of recruitment and honing activities. In total, 232 clinics were identified for the final evaluation sample (treatment clinics: N = 123; control clinics: N = 109). In September 2016, baseline data collection, including baseline household and client exit interviews, was completed for all AHME clinics.

The SP experiments were conducted orthogonally to the AHME treatment assignment; however, we include a AHME treatment indicator for all visits conducted based on the clinic assignment to the AHME treatment or control group.

For peer review only

For peer review only - http://bmjopen.bmj.com/site/about/guidelines.xhtml

A.1.3 Standardized Patient Recruitment, Training, and Pilot

We implemented two main survey methodologies: SPs and provider vignettes, which minimize bias in assessing provider practice and provider knowledge, respectively. For our SP data, we define visits as visits where the SP visited the clinic during operating hours and interacted with clinic staff, similar to an actual client presenting with similar conditions.

Other methods to assess quality of care have certain limitations that do not make them ideal for answering our research questions. Health and medical record data often do not exist in LMIC settings and when they do, they suffer from poor data quality. Direct observation is biased by the Hawthorne Effect. Patient exit interviews represent different patient sorting and patient mixes across clinics, and not only do clients not always understand medical jargon, but it is difficult to know precisely what medical condition the client has. Vignettes excel at assessing provider knowledge, but as for practice, vignettes are subject to social desirability bias and differ largely from practice measures (Kwan et al. 2019). For example, the "know-do gap" is a well-documented phenomenon in the literature referring to the difference between provider knowledge and provider practice (Das et al. 2015; Mohanan et al. 2015).

A.1.3.1 SP Cases

We implemented our SP study based on protocol from a previous SP study conducted in Nairobi, Kenya (Daniels et al. 2017; Kwan et al. 2019). A technical advisory group consisting of four Kenyan clinicians advised our team on case development, and all hired SPs participated in developing standardized narratives (e.g., name, age, family situation, living situation, etc.) for the SP case during training. The technical advisory group participated in SP training and advised on outcome measures for each case.

The childhood diarrhea case scenario for SPs were adapted from a pilot conducted in 2014 in Nairobi, Kenya. The case scenario developed for this study has two parts: (1) the SP narrative designed with the technical advisory group and the SP recruits during training and (2) the corresponding SP exit survey. The SP narrative describes the social milieu of the presenting caregiver as well as the situation that motivates the caregiver to access health care services for their child who is sick at home with diarrhea on the day of the visit. Figure A3 shows the case scenario narrative. Figure A4 shows the case scenario's attire for presenting at each clinic, alongside the opening statement, and some history questions to which the SPs are trained to provide pre-scripted responses.

For peer review only - http://bmjopen.bmj.com/site/about/guidelines.xhtml

Figure A3. Childhood diarrhea case scenario narrative

SP NARRATIVE AHME (adapted from Nairobi)

Standardized Case 1: Watery Diarrhoea

JOSEPHINE

Josephine is 28 years old and a merchandiser in Nairobi. Today she decided to go home earlier than usual because she left her daughter Diana feeling unwell in the house. Diana has been unwell for the past two days and Josephine was really getting worried about her. Since then she had diarrhoea six or seven times and was crying more than usual. As Josephine was making her way home, she received a call from her niece, informing her that Diana's diarrhoea had worsened again. Her husband is away travelling.

Diana is one and a half years old. Lately Josephine has tried to observe cleanliness in the house. But recently there has been some water shortage due to a burst city council pipe. She buys 2-3 20-liter Jelicans of water[1] per day and divides it for all her chores. She stores the drinking water in a five-liter plastic can. She boils it when she has some paraffin to spare after making the family meals. There is no provision for drainage system, and they have one toilet that is shared among many households within the plot. There is also no system for garbage collection, and it is always heaped just behind her house. (Josephine lives in a small village. She gets water from a well/river nearby. She stores her drinking water in a small pot or a jelican. She boils it when she has some paraffin/wood to spare after making the family meals. There is no provision for a drainage system, and they have one toilet that is shared among many households.)

Diana is still breastfeeding, has had all of her immunizations and has been a healthy baby. Lately, she has not been her usual self, and since last night she is having several bouts of watery stools – sometimes they have mucus and stickiness. They did not smell particularly foul though. She seemed a little weak and tired but was still playful. Diana's body was a little hot. She has also been crying more than usual, and it seemed she had some tummy ache. She was vomiting a little. She lost her appetite but was drinking lots of water. Josephine had prepared ORS before she left for work for her to be given during the day. On hearing of the many episodes of diarrhoea that Diana has been having, Josephine was worried. She decided to visit a nearby clinic on her way home.

For peer review only - http://bmjopen.bmj.com/site/about/guidelines.xhtml

Figure A4. Case scenario attire, opening statement, and sample history question responses

SP NARRATIVE **AHME (adapted from Nairobi)**

Josephine's dress:

1. Generally, Josephine is a very simple woman.
2. Wears smart and casual clothes, which are not expensive.
3. She doesn't wear excessive make-up, and most times she does not wear any at all.
4. She puts on simple doll shoes or rubber shoes and small earrings or studs.
5. She carries a very simple handbag sometimes a shopping bag and a kikoi/kanga.
6. In the coast, Josephine wears a long dress/skirt, Dera or a Buibui. In some parts of Homabay and towards Kisii, she does not wear trousers at all. She only wears long skirts.

Opening statement:

My child has been having diarrhea.
Kiswahli: Mtoto wangu ana hara/endesha.
Taveta: Dakitari mwana wangu efwaka
Rabai: Dakitari mwanangu yunahara
Luhya: Omwana wanje anyalala
Kamba: Ndakitali mwana wakwa nukwitua
Meru: Kana gakwa igakwatwa/ mwana okwa nakwarwa
Kalenjin: Taktari mondoe moet lakwenyun
Maasai: Nkitari, keloito (e)nkeraiai (e)nkoshoke
Luo: Daktari, nyathina diewo
Kikuyu: Mwana wakwa niaraharwo
Taita: Dakitari mwana wapowawefwaya
Kisii: Omwana one agosaa
Embu: Ndakitare mwana wakwa nearavarwa

History questions asked by the provider and their answers:

1. Q: How old is the child?
Mwanao ana umri gani?
A: 1 1/2 years old.
Mwaka mmoja unusi.

2. Q: How many times has she passed stools?
Amehara mara ngapi?
A: Many times.
Mara nyingi.

3. Q: How many times in the last 24 hours?
A: Maybe 6 or 7 times in the last two days.

4. Q: For how many days has she had this?
Kwa muda wa siku ngapi amekuwa hivi?
A: Two days ago but it wosened today.
Siku mbili iliyopita lakini ilimzidia mchana.

For peer review only - http://bmjopen.bmj.com/site/about/guidelines.xhtml

A.1.3.2 SP Recruitment and Training

Figure A5 shows the SP training agenda. SPs were extensively trained in risk mitigation sessions to avoid injections, taking medicine on the spot, unsafe and unsterile needles.

Figure A5. SP Training Agenda

SP TRAINING AGENDA

Week 1

	15-Jan-19 TUESDAY	16-Jan-19 WEDNESDAY	17-Jan-19 THURSDAY	18-Jan-19 FRIDAY
08.30 - 9:15	Registration and Introduction	Introduction to Group Work	Recap and presentation from each group	Recap and presentation from each group
09:15-10:30	Introduction to IPAK/ Admin	Review of SP cases in groups	Group work: Script and Narrative Development	Group reenactment of scripts using risk mitigation strategies
10:30 - 10:45	TEA BREAK	TEA BREAK	TEA BREAK	TEA BREAK
10:45 - 13:00	Introduction to IPAK/Admin<br>Introduction to SP cases	Review of SP cases in groups	Group reenactment of scripts and SP cases	Introduction to Exit Questionnaire
13:00 -14.00	LUNCH	LUNCH	LUNCH	LUNCH
14:00-15:00	Introduction to SP study	Group work: Script and Narrative Development	Risk Mitigation Strategies	Exit Questionnaire
15:00 - 16:00	Group SP into cases	Group work: Script and Narrative Development	Risk Mitigation Strategies	Exit Questionnaire
16:30 - 17:00	TEA BREAK	TEA BREAK	TEA BREAK	TEA BREAK

Week 2

	21-Jan-19 MONDAY	22-Jan-19 TUESDAY	23-Jan-19 WEDNESDAY	24-Jan-19 THURSDAY	25-Jan-19 FRIDAY
08.30 - 9:15	Exit Questionnaire	Mock interviews	Mock interviews for SP cases with practice recall questions	Clinicians Assessment of the SP/ mock interviews	Recap and presentation from each group
09:15-10:30					Mock interviews
10:30 - 10:45	TEA BREAK	TEA BREAK	TEA BREAK	TEA BREAK	TEA BREAK
10:45 - 13:00	Exit Questionnaire	Mock interviews	Mock interviews for SP cases with practice recall questions	Clinicians Assessment of the SP/ mock interviews	Mock interviews
13:00 -14.00	LUNCH	LUNCH	LUNCH	LUNCH	LUNCH
14:00-15:00	Introduction to Mock Interviews	Risk Mitigation Strategies	Mock interviews for SP cases with improvisation questions practice	Clinicians Assessment of the SP/ mock interviews	Mock interviews to practice recall questions
15:00 - 16:00	Mock interviews	Mock interviews	Mock interviews for SP cases with improvisation questions practice	Clinicians Assessment of the SP/ mock interviews	Mock interviews to practice recall questions
16:30 - 17:00	TEA BREAK	TEA BREAK	TEA BREAK	TEA BREAK	TEA BREAK

Week 3

	28-Jan-19 MONDAY	29-Jan-19 TUESDAY	30-Jan-19 WEDNESDAY	31-Jan-19 THURSDAY	01-Feb-19 FRIDAY
08.30 - 9:15	Risk Mitigation Strategies	Dry runs for the team	Dry runs for the team	Dry runs for the team	Final debriefing of the team
09:15-10:30					
10:30 - 10:45	TEA BREAK	TEA BREAK	TEA BREAK	TEA BREAK	TEA BREAK
10:45 - 13:00	Mock interviews to practice recall questions	Dry runs for the team	Dry runs for the team	Dry runs for the team	Screening of SPs by clinicians
13:00 -14.00	LUNCH	LUNCH	LUNCH	LUNCH	LUNCH
14:00-15:00	Mock interviews to practice recall and improvisation questions	Debriefing of the teams after dry runs	Debriefing of the teams after dry runs	Debriefing of the teams after dry runs	AX24 Last round of mock interviews practice recall and improvisation questions
15:00 - 16:00	Mock interviews to practice recall and improvisation questions	Debriefing of the teams after dry runs	Debriefing of the teams after dry runs	Debriefing of the teams after dry runs	Last round of mock interviews practice recall and improvisation questions
16:30 - 17:00	TEA BREAK	TEA BREAK	TEA BREAK	TEA BREAK	TEA BREAK

A.1.3.3 SP Pilot and Motivation for Experiments

Given the experience during the pilot, we designed experiments for demanding unnecessary medicines. Figure A6 shows the case scenario narratives with scripts for the two experiments: demanding amoxicillin and demanding albendazole.

For peer review only

For peer review only - http://bmjopen.bmj.com/site/about/guidelines.xhtml

Figure A6. Case Scenario Scripts for Demanding Unnecessary Medicine Experiments

Experiments for Standardized Case 1: Watery Diarrhoea

Only conduct the following experiments if assigned by your supervisor.

Experiment 1. Expressing Serious Concern and Demanding Unnecessary Drugs

Josephine goes to the facility, presents her case and on occasions when the provider refuses to give her anything/treat the baby, she tells him/her that she is really worried about her baby's condition and asks to be given (a) *dawa ya minyoo* (ABZ deworming medicines) or (b) Amoxyl (amoxicillin) for babies.

(a) **Demanding dawa ya minyoo (concern, abz):**
When assigned to this experiment, the SP presents worried and remembers another time when her baby was not feeling well and *dawa ya minyoo* calmed the baby. She wants the provider's advice and help in this concerning situation (otherwise she would just go to the chemist), but she is also convinced by her comfort that *dawa ya minyoo* will help.

At three possible moments when appropriate, the SP assigned this experiment can express the desire for *dawa ya minyoo*: (i) when the provider is writing a prescription or about to dispense drugs, (ii) when the doctor asks what the patient wants, or (iii) at the end of the interaction and if the provider hasn't given *dawa ya minyoo* (ABZ) yet, the SP stands up and seem as if she wants to leave then turn back and says to the doctor in a pleading tone:

"But doctor I'm really worried... Can you give me *dawa ya minyoo* for my baby? The last time she was sick, it helped."

(b) **Demanding Amoxyl (concern, amoxyl):**
When assigned to this experiment, the SP presents worried and remembers another time when her baby was not feeling well and *Amoxyl* calmed the baby. She wants the provider's advice and help in this concerning situation (otherwise she would just go to the chemist), but she is also convinced by her comfort that *Amoxyl* will help.

At three possible moments when appropriate, the SP assigned this experiment can express the desire for *Amoxyl*: (i) when the provider is writing a prescription or about to dispense drugs, (ii) when the doctor asks what the patient wants, or (iii) at the end of the interaction and if the provider hasn't given *dawa ya minyoo* (ABZ) yet, the SP stands up and seem as if she wants to leave then turn back and says to the doctor in a pleading tone:

"But doctor I'm really worried... Can you give me amoxyl for my baby? The last time she was sick, it helped."

For peer review only - http://bmjopen.bmj.com/site/about/guidelines.xhtml

A.1.5 Ethical Clearance

Ethical considerations for utilizing the SP method for this study

The AHME quantitative evaluation was granted clearance by the ethics committees at Kenya Medical Research Institute (No. KEMRI/RES/7/3/1; NON-SSC PROTOCOL NO. 372) and the Human Subjects Committee for Innovations for Poverty Action IRB-USA (IPA IRB Protocol #1085). The ethical clearance included all primary data collection activities for process quality analyses. This appendix describes the protocol for SP data collection.

All the SPs in this study were hired as field staff and participated in a three-week training, and a two-week pilot, and are required to participate in refresher trainings throughout fieldwork in order to mitigate any potentially harmful events, such as unsafe injections, invasive tests, and consumption of any medicines during encounters in the health sector.

Similar to other SP studies with similar designs and embedded in an intervention,[6] we sought a waiver of provider informed consent to conduct the SP study. The request for a waiver was based on a recent study commissioned by the United States Department of Health and Human Services to assess the ethics of simulated patient studies.[7] Supported by a pilot study conducted in Nairobi that validated the SP method in the Kenyan context,[8] both ethics committees approved the waiver request within the AHME evaluation study because (1) combining informed consent with the congregation of providers during trainings and the implementation of interventions during the study period posed threats to the scientific validity of the study objectives, as well as to the risk of SP detection, and (2) there is no more than minimal risk of participation to the SPs or providers, as reported in the Nairobi SP pilot and validation study (Daniels et al. 2017).

Ethics committee approvals with the waiver of informed consent were provided conditional on our agreement to return to all clinics visited by SPs to disclose the SP study to them and to provide them with an opportunity to ask questions and discuss any concerns. During January 1–23, 2020, we informed all clinics that received SPs and that were not closed permanently at that time.

All full questionnaires, case scripts, and the granted request for a waiver of informed provider consent are available upon request.

A.1.6 Fieldwork Protocol

Clinics were assessed for SP visit eligibility and mapping was also conducted to determine which clinics had been closed. The following protocol was implemented ahead of fieldwork initiation:

- Ineligible – SPs should not visit any clinics that are labelled as such in the schedule. For example,
    - 2 clinics – do not accept walk-ins from individuals who are not employees of a firm only
    - 1 clinic – fistula clinic
    - 1 clinic – excluded due to security issues
    - There could be more.
- Check closed status – Supervisors should double check whether these are closed at the time of fieldwork before sending any SPs
    - 8 clinics based on mapping activity
    - There could be more.
- Non-consenting clinics – Supervisors should check mapping and other surveys implemented for the AHME impact evaluation (exit interviews; clinic surveys) to see whether consent has been

For peer review only - http://bmjopen.bmj.com/site/about/guidelines.xhtml

provided for at least one of these AHME surveys at the time of SP fieldwork. If consent has not been provided at any of these, do not conduct any SP interactions.

To reduce SP detection at the clinic while maintaining fieldwork protocol, the following were implemented:

1. Familiars at sampled clinics. Before conducting any interactions in each region, QAs should introduce the full clinic list to the SPs. The clinics should be described one-by-one. SPs should review all the clinics in the sample and identify to the QA any clinics where they know a friend or family member who is affiliated to that clinic. The QA.
2. SP case narratives. When a team arrives at a new fieldwork area, the QA should debrief the full team on: (i) the area, (ii) the clinics in the area, (iii) contextual adaptations, including dress and language, to each of the cases based on the new fieldwork.
3. Isolated/rural clinics. Isolated/rural clinics should be identified by each QA from their mapping experience. For each SP that goes to that clinic, a story should be constructed for three things: (i) from where the SP character is traveling, (ii) to where the SP character is traveling, (iii) local names of people and places the SP character visited or will visit.
4. SP visit timing. The childhood diarrhea case should be sent in the afternoon.
5. SP sequencing. SP cases or SPs who have a lower risk of detection should be sent before SP cases or SPs who have a higher risk of detection.
6. SP spacing. The spacing between SP visits should be controlled by the QA. First, more than one SP should not be sent at the same time unless the QA knows that there are 5 or more patients waiting to be seen at a given time on a given day. Second, QAs should wait 2-3 days between SP interactions at clinics that see <5 patients per day or do not have strangers coming for services.

For peer review only - http://bmjopen.bmj.com/site/about/guidelines.xhtml

APPENDIX B: SUPPLEMENTAL RESULTS

Figure B1. Effects Pre-demanding Amoxicillin vs. Pre-Demanding Albendazole

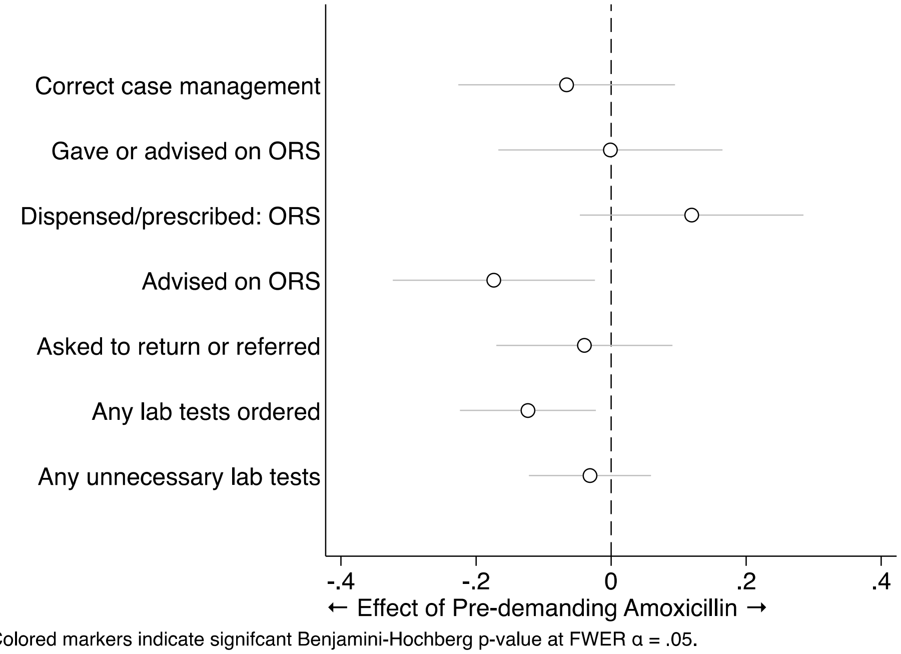

For peer review only - http://bmjopen.bmj.com/site/about/guidelines.xhtml

Figure B2. Effects of Demanding (Pooled Albendazole and Amoxicillin)

(A) Note: Colored markers indicate significant p-values at \alpha = 0.05

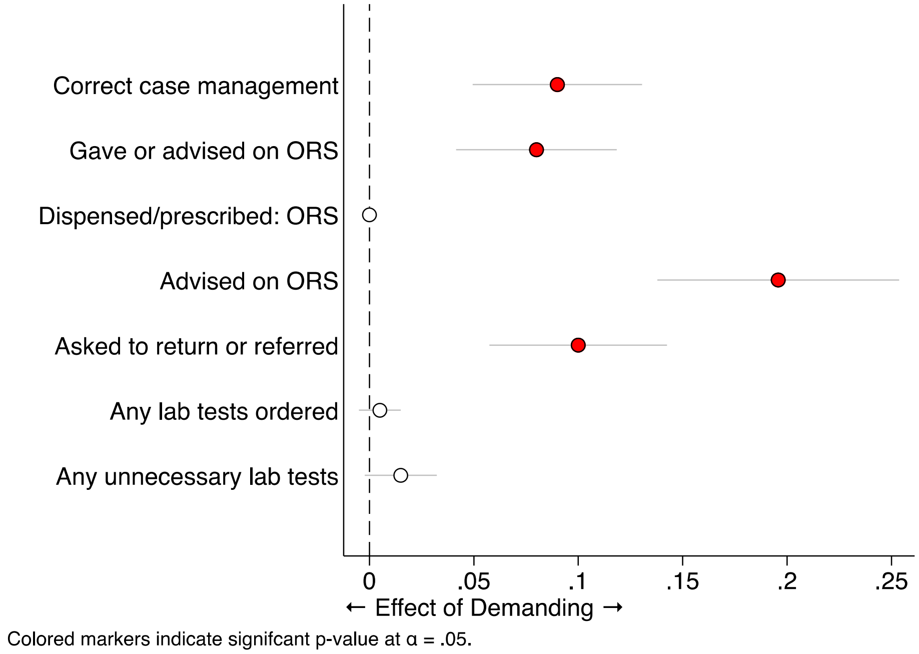

(B) Colored markers indicate significant with Benjamini-Hochberg corrected p-values at \alpha = 0.05

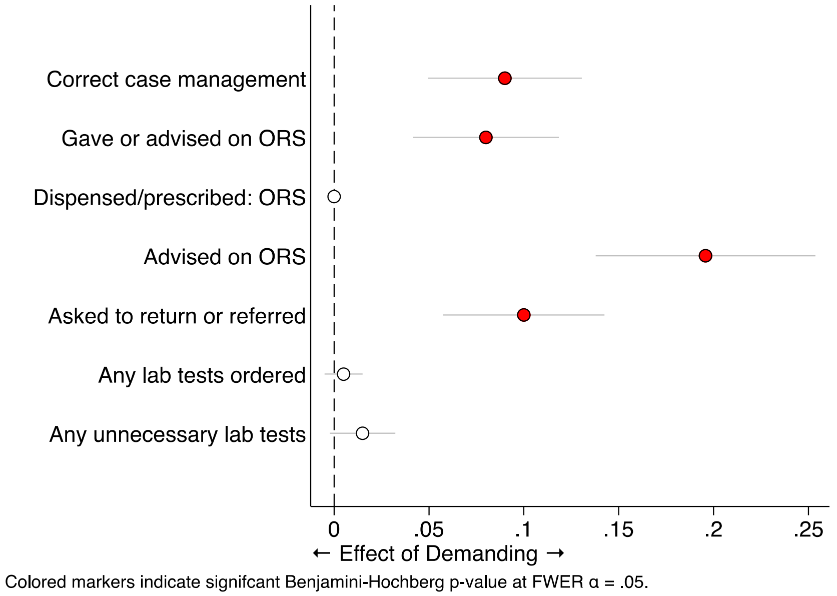

Figure B3. Childhood diarrhea knowledge vs. practice

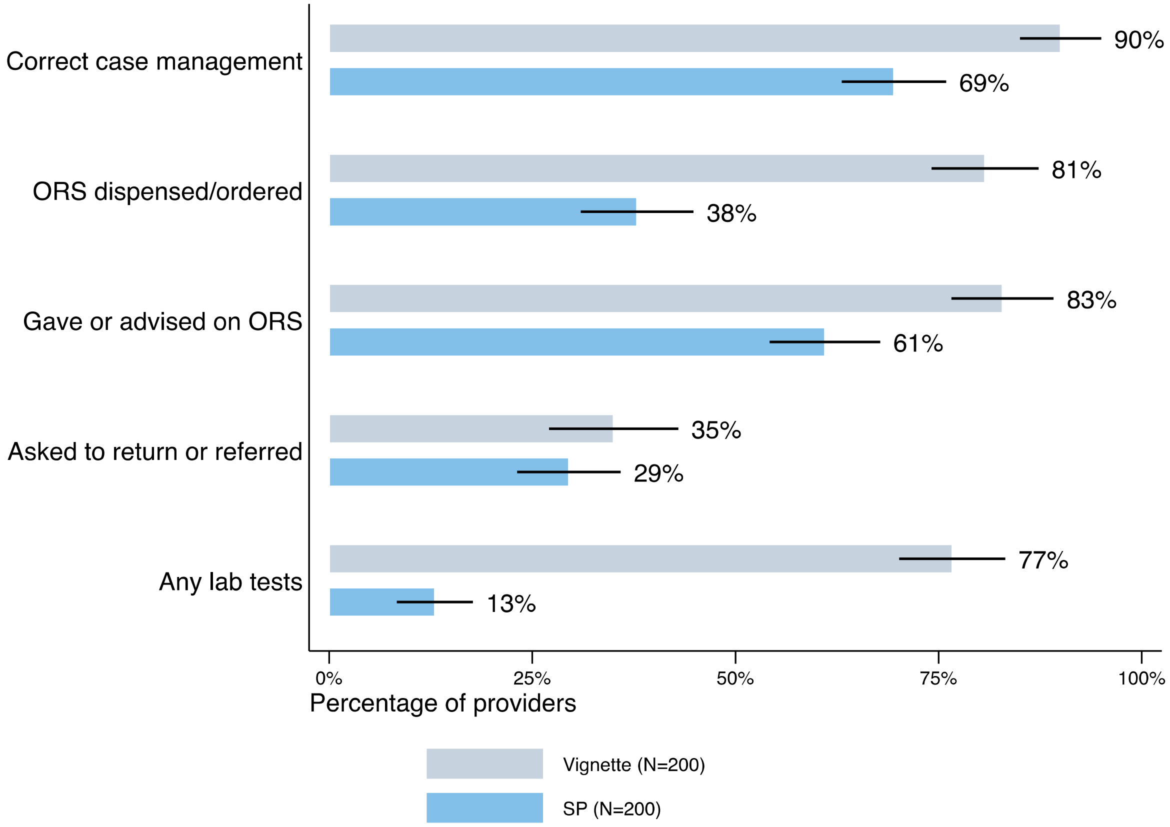

ew only

Appendix Table B1. Effects of demanding albendazole or amoxicillin vs. pre-demanding on quality of care outcomes

	(1) Correct case management	(2) Gave or advised on ORS	(3) Asked to return	(4) Asked to return or referred	(5) Dispensed/ prescribed: ORS	(6) Dispensed/ prescribed: Zinc	(7) Any non-efficacious medicines	(8) Dispensed/ prescribed: Albendazole	(9) Dispensed/ prescribed: Any antiparasitic	(10) Dispensed/ prescribed: Amoxicillin	(11) Dispensed/ prescribed: Any antibiotic
Albendazole post-demanding											
Coefficient	0.098	0.053	0.113	0.110	-0.035	-0.060	-0.015	0.142	0.115	-0.017	-0.012
Standard Error	(0.037)	(0.038)	(0.038)	(0.038)	(0.032)	(0.032)	(0.033)	(0.027)	(0.029)	(0.019)	(0.034)
p-value	[0.009]	[0.166]	[0.003]	[0.004]	[0.279]	[0.063]	[0.638]	[0.000]	[0.000]	[0.383]	[0.718]
Amoxicillin post-demanding											
Coefficient	0.082	0.108	0.086	0.090	0.037	0.063	0.016	-0.148	-0.130	0.018	0.014
Standard Error	(0.039)	(0.041)	(0.040)	(0.040)	(0.034)	(0.034)	(0.034)	(0.027)	(0.032)	(0.020)	(0.038)
p-value	[0.039]	[0.009]	[0.033]	[0.026]	[0.281]	[0.065]	[0.640]	[0.000]	[0.000]	[0.383]	[0.719]
Pre-demanding Group Mean	0.650	0.570	0.245	0.245	0.360	0.390	0.670	0.190	0.258	0.095	0.584
Observations	400	400	400	400	400	400	400	400	380	400	380

Note: The table shows ordinary least squares regressions using standardized patient (SP) data. Robust standard errors are in parentheses, clustered at SP individual and clinic levels. Two-sided p-values in brackets. All models contain SP fixed effects and control for the 0-1 AHME treatment indicator, a binary indicator for whether a clinic was assigned to receive an SP demanding albendazole at the end of the visit (Albendazole post-demanding) or whether a clinic was assigned to receive an SP demanding amoxicillin at the end of the visit (Amoxicillin post-demanding). All outcomes in models (1)-(11) are binary variables where if the action occurred during the visit 1=yes; 0=otherwise for both pre-demanding and post-demanding time points for the visit.

For peer review only - http://bmjopen.bmj.com/site/about/guidelines.xhtml

Appendix Table B2. Effects of post-demanding albendazole (vs. post-demanding amoxicillin) on childhood diarrhea care management outcomes

	(1) Correct case management	(2) Gave or advised on ORS	(3) Asked to return	(4) Asked to return or referred	(5) Dispensed/ prescribed: ORS	(6) Dispensed/ prescribed: Zinc	(7) Any non-efficacious medicines	(8) Dispensed/ prescribed: Albendazole	(9) Dispensed/ prescribed: Any antiparasitic	(10) Dispensed/ prescribed: Amoxicillin	(11) Dispensed/ prescribed: Any antibiotic
Albendazole post-demanding											
Coefficient	0.005	-0.074	0.022	0.014	-0.086	-0.148	-0.038	0.348	0.292	-0.041	-0.031
Standard Error	(0.077)	(0.083)	(0.076)	(0.076)	(0.080)	(0.080)	(0.081)	(0.060)	(0.069)	(0.048)	(0.087)
p-value	[0.944]	[0.373]	[0.772]	[0.854]	[0.285]	[0.066]	[0.643]	[0.000]	[0.000]	[0.388]	[0.723]
Post-demanding Amoxicillin Group Mean	0.724	0.673	0.286	0.296	0.398	0.449	0.704	0.031	0.146	0.112	0.607
Observations	200	200	200	200	200	200	200	200	190	200	190

Note: The table shows ordinary least squares regressions using standardized patient (SP) data for the post-demanding phase of the N=200 SP visits. Robust standard errors are in parentheses, clustered at SP individual and clinic levels. Two-sided p-values in brackets. All models contain SP fixed effects and control for the 0-1 AHME treatment indicator, a binary indicator for whether the visit was Albendazole post-demanding. All outcomes in models (1)-(11) are binary variables where if the action occurred during by the end of the visit 1=yes; 0=otherwise.

For peer review only - http://bmjopen.bmj.com/site/about/guidelines.xhtml

Appendix Table B3. Composition of medicines prescribed across all SPs who demanded albendazole versus amoxicillin.

		Demanding Albendazole (N=102)		Demanding Amoxicillin (N=98)	
**Type**	**Medicine**	*Frequency*	*Percentage*	*Frequency*	*Percentage*
Correct	ORAL REHYDRATION SALTS	32	31%	38	39%
	ZINC	33	32%	42	43%
	ORAL REHYDRATION SALTS AND ZINC SULPHATE	2	2%	2	2%
Antiparasitic	ALBENDAZOLE	35	34%	3	3%
	ARTEMETHER LUMEFANTRINE	5	5%	4	4%
	QUININE	1	1%	0	0%
	DILOXANIDE	0	0%	4	4%
	DIHYDROARTEMISININ AND PIPERAQUINE PHOSPHATE	0	0%	1	1%
	NITAZOXANIDE	0	0%	1	1%
Antibiotic	METRONIDAZOLE	27	26%	27	28%
	SULFAMETHOXAZOLE AND TRIMETHOPRIM	17	17%	21	21%
	METRONIDAZOLE BENZOATE	12	12%	12	12%
	AMOXICILLIN	8	8%	11	11%
	DILOXANIDE FUROATE METRONIDAZOLE DICYCLOMINE HCL	6	6%	2	2%
	AMPICILLIN AND CLOXACILLIN	3	3%	3	3%
	CEFALEXIN	2	2%	2	2%
	ERYTHROMYCIN	2	2%	2	2%
	CEFIXIME	2	2%	0	0%
	CHLORAMPHENICOL PALMITATE	1	1%	2	2%
	AMOXICILLIN AND POTASSIUM CLAVULANATE	1	1%	0	0%
	CIPROFLOXACIN	1	1%	0	0%
	CHLORAMPHENICOL	1	1%	0	0%
	DILOXANIDE FUROATE METRONIDAZOLE	1	1%	0	0%
	ERYTHROMYCIN ETHYL SUCCINATE	1	1%	0	0%

For peer review only - http://bmjopen.bmj.com/site/about/guidelines.xhtml

For peer review only

	CHLORAPHENICOL AND RETINOL	0	0%	1	1%
	ROXITHROMYCIN	0	0%	1	1%
	AMPICILLIN	0	0%	1	1%
	CEFADROXIL	0	0%	1	1%
Other	PARACETAMOL	22	22%	23	23%
	IBUPROFEN	5	5%	3	3%
	LEVAMISOLE	4	4%	0	0%
	IBUPROFEN AND PARACETAMOL	3	3%	1	1%
	HYOSCINE BUTYLBROMIDE	2	2%	0	0%
	PROMETHAZINE HYDROCHLORIDE	1	1%	5	5%
	SACCHAROMYCES BOULARDII	1	1%	1	1%
	MULTIVITAMIN	1	1%	1	1%
	KAOLIN PECTIN	1	1%	0	0%
	AMINOSIDINE	1	1%	0	0%
	CHLORPHENIRAMINE	1	1%	0	0%
	CETIRIZINE	1	1%	0	0%
	DICYCLOVERINE HYDROCHLORIDE SIMETHICONE	1	1%	0	0%
	GUAIFENESIN	1	1%	0	0%
	VITAMIN A	1	1%	0	0%
	LOPERAMID HYDROCHLORIDE	0	0%	2	2%
	DOMPERIDONE	0	0%	1	1%
	PIROXICAM	0	0%	1	1%
	SALBUTAMOL	0	0%	1	1%
Unknown	UNKNOWN	0	0%	1	1%

For peer review only

BMJ Open

Do private providers give patients what they demand, even if it is inappropriate? A randomized study utilizing unannounced standardized patients in Kenya

Journal:	*BMJ Open*
Manuscript ID	bmjopen-2021-058746.R1
Article Type:	Original research
Date Submitted by the Author:	09-Feb-2022
Complete List of Authors:	Kwan, Ada; University of California San Francisco, Department of Medicine; University of California Berkeley School of Public Health, Division of Health Policy and Management<br>Boone, Claire; University of California Berkeley School of Public Health, Division of Health Policy and Management<br>Sulis, Giorgia ; McGill University, Department of Epidemiology, Biostatistics and Occupational Health<br>Gertler, Paul; University of California Berkeley Haas School of Business
**Primary Subject Heading**:	Global health
Secondary Subject Heading:	Health services research, Health policy, Health economics, Paediatrics, Research methods
Keywords:	Health policy < HEALTH SERVICES ADMINISTRATION & MANAGEMENT, Health economics < HEALTH SERVICES ADMINISTRATION & MANAGEMENT, Quality in health care < HEALTH SERVICES ADMINISTRATION & MANAGEMENT, International health services < HEALTH SERVICES ADMINISTRATION & MANAGEMENT, Public health < INFECTIOUS DISEASES, Community child health < PAEDIATRICS

SCHOLARONE™
Manuscripts

I, the Submitting Author has the right to grant and does grant on behalf of all authors of the Work (as defined in the below author licence), an exclusive licence and/or a non-exclusive licence for contributions from authors who are: i) UK Crown employees; ii) where BMJ has agreed a CC-BY licence shall apply, and/or iii) in accordance with the terms applicable for US Federal Government officers or employees acting as part of their official duties; on a worldwide, perpetual, irrevocable, royalty-free basis to BMJ Publishing Group Ltd ("BMJ") its licensees and where the relevant Journal is co-owned by BMJ to the co-owners of the Journal, to publish the Work in this journal and any other BMJ products and to exploit all rights, as set out in our licence.

The Submitting Author accepts and understands that any supply made under these terms is made by BMJ to the Submitting Author unless you are acting as an employee on behalf of your employer or a postgraduate student of an affiliated institution which is paying any applicable article publishing charge ("APC") for Open Access articles. Where the Submitting Author wishes to make the Work available on an Open Access basis (and intends to pay the relevant APC), the terms of reuse of such Open Access shall be governed by a Creative Commons licence – details of these licences and which Creative Commons licence will apply to this Work are set out in our licence referred to above.

Other than as permitted in any relevant BMJ Author's Self Archiving Policies, I confirm this Work has not been accepted for publication elsewhere, is not being considered for publication elsewhere and does not duplicate material already published. I confirm all authors consent to publication of this Work and authorise the granting of this licence.

For peer review only

Do private providers give patients what they demand, even if it is inappropriate? A randomized study utilizing unannounced standardized patients in Kenya

Ada Kwan[1,2], Claire E. Boone[2], Giorgia Sulis[3], and Paul J. Gertler[4]

[1]Division of Pulmonary and Critical Care Medicine, University of California San Francisco School of Medicine, San Francisco, CA, United States
[2]Division of Health Policy and Management, University of California, Berkeley School of Public Health, Berkeley, CA, United States
[3]Department of Epidemiology, Biostatistics, and Occupational Health, McGill University, Montreal, Canada
[4]Haas School of Business, University of California, Berkeley, Berkeley, CA, United States

For peer review only

Abstract

Introduction: Low and varied quality of care has been demonstrated for childhood illnesses in low-and middle-income countries. Some quality improvement strategies focus on increasing patient engagement; however, evidence suggests that patients demanding medicines can favor the selection of resistant microbial strains in the individual and the community if drugs are inappropriately used. This study examines the effects on quality of care when patients demand different types of inappropriate medicines.

Methods: We conducted an experiment where unannounced standardized patients (SPs), locally recruited individuals trained to simulate a standardized case, present at private clinics. Between March 8 and May 28, 2019, 10 SPs portraying caretakers of a watery diarrhea childhood case scenario (in absentia) conducted N=200 visits at 200 private, primary care clinics in Kenya. Half of the clinics were randomly assigned to receive an SP demanding amoxicillin (an antibiotic); the other half, an SP demanding albendazole (an antiparasitic drug often used for deworming), with other presenting characteristics the same. We used logistic and linear regression models to assess the effects of demanding these inappropriate medicines on correct and unnecessary case management outcomes.

Results: Compared to 3% among those who didn't demand albendazole, the dispensing rate increased significantly to 34% for those who did (adjusted odds ratio 0.06, 95% CI: 0.02-0.22, p<0.0001). Providers did not give different levels of amoxicillin between those demanding it and those not demanding it (adjusted odds ratio 1.73, 95% CI: 0.51-5.82). Neither significantly changed any correct management outcomes, such as treatment or referral elsewhere.

Conclusion: Private providers appear to account for both business-driven benefits and individual health impacts when making prescribing decisions. Additional research is needed on provider knowledge and perceptions of profit and individual and community health trade-offs when making prescription decisions after patients demand different types of inappropriate medicines.

Keywords: prescribing practices, diagnostics, standardized patients, quality, health care providers, Kenya, private health sector

Abstract word count: 294

Word count: 4999 (with strengths and limitations section)

Corresponding Author:

Ada Kwan, PhD MHS

Postdoctoral Scholar

University of California, San Francisco

Department of Medicine

1001 Potrero Avenue

San Francisco, CA 94110

ada.kwan@ucsf.edu

For peer review only

Strengths and limitations of this study

- Use of the standardized patient (SP) method, where locally recruited individuals are trained to present the same scenario, offers the ability to compare provider practice across a sample of private health providers who are faced with a patient who demanded inappropriate care.
- This study uses a randomized design to causally determine relative differences in provider behavior when two inappropriate medicines are demanded by unannounced SPs with random assignment, with the presenting scenario the same otherwise.
- A limitation of the SP method is that the findings do not fully reflect real patient behavior.
- This study is not able to assess the level of provider awareness regarding the appropriateness of each medicine or the condition under investigation.

For peer review only

Introduction

Individuals seeking health care services sometimes demand inappropriate medicines, such as antibiotics, based on the widespread misperception that this would lead to faster and better recovery.(1,2) Regardless of adequate training and knowledge of clinical practice guidelines, providers may grant these requests to facilitate patient satisfaction and to avoid negative judgments.(3) For-profit providers may be concerned that these negative judgments, and their overall reputation, can reduce the likelihood of patients returning for subsequent visits, which can affect their bottom line. Prescribing behaviors that arise from these concerns may vary based on the extent to which medicines demanded are harmful or perceived as such and, in the private sector, profitable.(4–8) In this paper, we study the effects of patients demanding two different inappropriate medicines, as examples of trade-offs providers might make between risks, profits, and patient satisfaction.(6,7,9,10)

These dynamics pertain to policy. Understanding the relationship between inappropriate dispensing behaviors and what patients demand from providers is important for designing quality improvement interventions. Public health authorities and many studies cite the overuse and misuse of antimicrobials as the main drivers of drug-resistance.(11) However, there is at best limited literature on the effects of when patients demand medicines on provider prescribing behavior in low- and middle-income countries (LMICs).(6,7) On the patient side, studies in high-income countries suggest that patient and provider knowledge, attitudes, and expectations are important drivers of antibiotic prescriptions.(12–14) One example is the notion of patient activation, or when "patients who have the *motivation, knowledge, skills, and confidence* to make *effective* decisions to manage their health" (emphasis ours).(15) Patient activation has been extensively studied in the United States, and this research emphasizes the potential for interventions that increase informed and "active" patients, particularly because of its association with better health and health care outcomes.(16,17) However, patient activation is different from when patients demand antibiotics that are inappropriate for their conditions. Further, many of these studies report associations and cannot differentiate whether increased engagement results in increased quality of care or the reverse. Thus, constructing effective interventions on patient engagement becomes challenging if actors or mechanisms for intervening to improve care are unclear. This suggests that the patient's role could have a larger influence on better care relative to the provider. That patients can have a larger influence on services begs the question, *What happens when patients demand different types of inappropriate care?*

Our study's objective is to examine the role of patient demand for inappropriate care, on prescribing and dispensing practices for childhood diarrhea in Kenya. The Government maintains explicit guidelines for childhood diarrhea case management (see Appendix A1),(18) and we utilize the standardized patient (SP) method, which provides an unbiased way to compare multiple providers because of a standardized case scenario presentation, to measure care levels. We draw on childhood diarrhea for several reasons. First, several studies have validated the use of the SP method for examining childhood diarrhea, including in Kenya.(19–23) Over other existing quality of care methods, the SP method has many advantages and also controls for patient mix and sorting. For example, provider surveys measure provider knowledge rather than actual practice; exit interviews suffer from recall bias and clients may also not be able to discern specific clinical actions; providers may perform differently under direct observation, known as the Hawthorne effect; and in these settings, the quality of administrative data or records is often

varied, if it exists at all.(8,23) Accruing evidence from SP studies on childhood diarrhea across LMICs demonstrate that quality of care is low and varied for correct management of childhood illnesses.(19,20,24–29)

Second, although the global burden of diarrheal disease is declining over time, it remains a major concern in LMICs where poor sanitation and hygiene along with indiscernibly varied quality health care make this health condition among children common and often life-threatening. With 1.73 billion episodes a year, diarrhea remains one of the leading causes of child morbidity and mortality worldwide.(30,31)

Third, diarrhea is an interesting condition to examine the role of patient demand on appropriate and inappropriate care. Diarrhea is defined as an increase in frequency of bowel movements (usually three or more per day), accompanied by a decrease in stool consistency.(32) Although a wide range of pathogens can cause diarrheal disease, consumption of contaminated food or water and interpersonal contacts in poor hygiene conditions constitute a common denominator. Rotavirus, *Escherichia coli*, *Cryptosporidium spp.* and *Shigella spp.* are the most common causal agents in lowest income settings.(33) The World Health Organization (WHO) Integrated Management for Childhood Illness handbook was published in 2005 to provide a structured and simplified approach to the assessment and therapeutic management of children presenting with various clinical pictures in first-level primary care facilities, particularly in resource-limited areas.(34) With respect to diarrhea, antimicrobial treatment is only recommended under selected circumstances (e.g., evidence of blood in the stool).

This study contributes to the literature in several ways. First, this study adds to the understanding of how pervasive is overprescription. Recent studies on health conditions, including common childhood illnesses, in LMICs show that the overuse of medicines pose dangers of resistance that have individual and public health level consequences.(24,25,35) Second, we provide experimental evidence showing that patient-related determinants influence appropriate and inappropriate treatment.(1,2,36) Two other studies to our knowledge examine the effects of patient demand specifically on the rates of antibiotic dispensing with the SP method and find that (i) SPs who share knowledge that antibiotics are inappropriate in China were less likely to receive antibiotics, and (ii) antibiotic prescription rates reduced when SPs demanded them alongside a statement that they would make the purchase elsewhere.(6,7) In an LMIC setting, which is underrepresented in the literature on this topic, this study additionally extends the current literature on the role of caregivers demanding two types of antimicrobial medicines for a condition that, for most children with this condition, requires only supportive treatment. The majority of diarrhea cases do not need microbial therapy and only require supportive treatment, such as rehydration.(32,34) Third, this study can help inform governments that are committed to universal access to high quality of care worldwide. Understanding how quality of care can be improved is critical, particularly in the private sector in countries where a substantial amount of care is provided by the private sector.(37) This study contributes to the knowledge of quality improvement mechanisms by understanding the provision of support treatment, as well as the provision of unnecessary or potentially harmful treatment at point of care.

For peer review only - http://bmjopen.bmj.com/site/about/guidelines.xhtml

Methods

This study examines standardized patient (SP) and provider vignette data collected in 2019 across 200 private clinics spread across 35 of Kenya's 47 counties. **Figure 1** shows a map of Kenya and a CONSORT diagram for clinic sampling. For this study, we exploited the private clinic sample (N=232) for the impact evaluation of a program called AHME and excluded clinics that were ineligible to receive walk-ins for childhood illnesses (4), were located in an area with security concerns (1), did not consent to the AHME evaluation study (7), or were permanently closed (14). We did not capture data from 6 clinics, which turned away the SP upon arrival. The program is not the focus of this study, but additional details related to the program and clinic sample are provided in Appendix A2 when relevant for this study, including AHME assignment across demanding arms (Appendix Table A1).

Data Sources

Between March 8 and May 28, 2019, 200 unannounced SP visits were completed at 200 private Kenyan clinics. Data was captured at two moments during the interaction: "pre-demanding" includes actions before the SP demanded the assigned medicine, and "post-demanding" includes all actions by the completion of the visit. We analyze N=200 pre-demanding and N=200 post-demanding observations for a childhood diarrhea case scenario. Using Stata 16 (StataCorp, College Station, TX, USA), half the clinics were randomly assigned to receive an SP demanding albendazole, and the other half received an SP demanding amoxicillin. SP requests were done at the end of the visit or earlier only if it was necessary to avoid an unusual visit.

The scenario represents a 28-year-old mother who comes to the clinic with a 1.5-year-old child at home sick with watery diarrhea (see **Table 1**). If probed by the provider, the SP is trained to share that the child is a little hot and has passed approximately 6-7 stools in the last two days. This study follows the design and protocol with sample size calculations based on the childhood diarrhea SP case scenario described in Daniels et al. (2017).(22) In our study, the SP visits were conducted by 10 females locally recruited, trained, and hired as SPs. All SPs were seemingly healthy, so providers would not detect and treat other health ailments that were unrelated to the presenting scenario. All data reflect quality measures for SPs seeking walk-in, outpatient services. Appendix A3 contains additional details on SP case development, recruitment, training, piloting, and sample size calculations (Appendix Table A2).

The SP method minimizes bias in assessing provider practice. To assess care provided to patients in LMIC settings, the literature describes several methodologies, including direct observation, administrative or medical record abstraction, client exit interviews, provider vignettes, and standardized patients (SPs). Each method has its own interpretation and set of advantages and disadvantages which is described at length elsewhere.(8,38,39) To identify the effect of what happens when a patient demands an inappropriate antibiotic or antiparasitic medicine, we randomly assigned whether the SP would demand amoxicillin or albendazole, respectively. Importantly, both medications are considered unnecessary for a child with watery diarrhea. Both medications also have harmful effects for the community if systematically and unnecessarily used. Appendix A3.3 includes details on how these two medicines were selected. The SP method has the advantage that the researchers know the true condition of the 'patient' which is not possible when examining data derived from real patients. SP data particularly allows for providers across different facilities to be compared against the exact same patient scenario and is

For peer review only

thus increasingly considered the gold standard for measuring provider practice across a sample of providers that lack standardized health records.

To ensure accurate and comprehensive recall, within 1 to 3 hours after each SP visit, SPs completed an exit questionnaire administered by a fieldwork supervisor. The exit questionnaire collected information regarding the SP's visit, including time of arrival, time of departure, history questions asked, diagnosis, lab tests ordered, medicines dispensed and prescribed, counseling given, and a subjective assessment of the visit. Further, for each visit, SPs and their supervisors attempted to identify all providers seen by the SPs. The list of providers formed the provider survey sampling frame.

Literature on quality of care in LMICs shows that large differences exist between what health care providers know and do.(40) To measure whether providers know what to do in this setting, we additionally analyze data from the provider survey conducted between November and December 2019 among providers who saw SPs. The provider survey included a vignette module to assess knowledge based on a childhood diarrhea vignette case matching the SP case scenario, in addition to capturing provider characteristics through interview.

Outcomes

Using SP and vignette data, we constructed binary measures for our main outcomes of interest: correct case management and whether any unnecessary medicines were prescribed or dispensed, since one aspect of quality of care is not only dispensing correct medicines but also *not* dispensing inappropriate medicines. Benchmarked against national guidelines, correct case management refers to the minimal and essential actions for childhood watery diarrhea case management (see Appendix Figure A1).(18) Visits were coded as being correctly managed (=1) if the provider did any one of the following: gave oral rehydration salts (ORS), advised on ORS, referred the SP, or asked the SP to return; 0, otherwise (**Table 1**). We classified ORS and zinc to be appropriate, and a provider was coded as ordering any unnecessary medicines if others were prescribed or dispensed. We define 'prescribe/dispense' as a term to capture the provider's intention to give a medicine to the patient, regardless of whether the SP walked away with it: 'prescribe' captured a situation where the provider may have written a prescription, including when the SP may not have actually received it (e.g., a stockout); 'dispense' captured a situation where the provider may have given the medicine, including when the provider may not have written a prescription. We examine whether the provider prescribes/dispenses amoxicillin or albendazole. Additionally, whether any antibiotic and/or any antiparasitic (including antimalarials) were assessed.

Statistical Analysis

We first conducted difference-in-means tests on clinic characteristics uncorrelated as a balance check to confirm the random assignment of demanding experiments to clinics were balanced. Next, we computed adjusted odds ratios (aOR) with 95% confidence intervals (CIs) from a logistic regression model, while controlling for differences that arose from our design, including a binary SP experiment variable (0 if the SP was assigned to demand albendazole; 1 if assigned to demand amoxicillin); the binary AHME treatment indicator representing whether the clinic associated with the SP visit received the AHME intervention (= 1) or was assigned to the control arm (= 0), which was randomized independently from the SP experiment (see Appendix Table

For peer review only - http://bmjopen.bmj.com/site/about/guidelines.xhtml

A1 for AHME assignment); and SP actor fixed effects, as illustrated in previous SP studies with similar designs. The parameter of interest is the SP experiment coefficient which is interpreted as the effect of demanding amoxicillin relative to albendazole on the outcome of interest. We complemented these analyses with ordinary least squares regression to assess differences in outcomes across the demanding experiments. It is important to note that our estimates correspond with the expected average quality of care and demanding differences if the clinics were selected randomly by a patient in the country.

Analyses using SP data were conducted at the SP-provider visit level. When SP data were linked to provider survey data, the unit of observation is a successful (i.e., completed) SP-provider visit with provider survey responses from the provider seen during the SP visit. All data analyses were performed with Stata 16, and de-identified interaction data with variables and code needed to re-create the results reported in this article are available.(41)

Patient and Public Involvement

Patients or the public were not involved in the design, or conduct, or reporting, or dissemination plans of our research.

Ethical Review

This study obtained IRB approval under the AHME impact evaluation study from Innovations for Poverty Action's independent IRB board, Protocol #1085, and from Kenya Medical Research Institute, Non-SSC Protocol #372. The study also received local research permission in Kenya National Commission for Science, Technology and Innovation Permit #NA-COSTI/P/19/5343/28310. We registered our study with the American Economic Association Registry, trial #AEARCTR-0000217, and the Pan African Clinical Trial Registry, trial #PACTR201502000770329. Appendix A4 provides more details on ethical considerations for utilizing the SP method for this study.

Results

A total of 200 unannounced SP-provider visits were successfully conducted by 10 SPs at a total of 200 different private health clinics across 34 of the 47 counties in Kenya (**Figure 1**, additional fieldwork details in Appendix A5). To ensure that the experiment was successfully randomized, we checked differences in means for the clinics assigned an SP demanding amoxicillin or albendazole across various characteristics (**Table 2**). Since the groups randomly assigned to receive SPs demanding different medicines are balanced on data from the year SP visits were conducted (i.e., the absolute difference between the mean value in the two groups is not different from zero), we can rely on our statistical model assumption that the randomization of demanding assignments created exchangeable treatment arms to assess the impacts of demanding different unnecessary drugs. Thus, we can interpret our coefficient of interest as an unbiased estimate of the effect of demanding each medicine on our outcomes.

Table 3 provides summary statistics for the N=200 SP post-demanding observations. Nearly 40% of providers were female and just over half of the visits were conducted with a provider that appeared between 30 and 50 years of age. More SPs saw a medical doctor or clinical officer

For peer review only - http://bmjopen.bmj.com/site/about/guidelines.xhtml

(33%) than other provider types, followed by a nurse or midwife (30%). On average, there were approximately 1.55 individuals waiting in the waiting room when the SP arrived (to capture how busy the clinic was in lieu of utilization data), and each SP visit lasted 6.65 minutes with the provider, who asked on average 4.46 history questions. Among the visits, 15% resulted in a correct diagnosis or suspicion of watery diarrhea, and 75% of the visits were correctly managed with 31% of SPs asked to return and a very small percentage (6%) referred elsewhere. Despite 75% of the SP visits being correctly managed in practice, 90% of the visits had a provider who knew how to correctly manage the case as measured in the administered provider vignette (see Appendix Figure B1 for more comparisons across knowledge and practice). Because outcomes that were captured before demanding ("pre-demanding") cannot be entirely interpreted as a complete interaction, we only report post-demanding measures (see Appendix Figures B2 and B3 for pre-demanding outcomes).

Effects of Demanding on Levels of Correct and Unnecessary Services

Figure 2 reports aORs comparing demanding albendazole versus demanding amoxicillin across various binary quality of care outcomes, adjusting for the AHME treatment assignment and SP individual fixed effects. We did not find that the type of unnecessary medicine demanded had an estimated effect on correct case management or any of its components (advising on ORS, giving or advising on ORS, asking to return, or referring the patient for any reason). However, the aOR of being dispensed or prescribed zinc, which is advised within the minimum package for facility case management as per the Kenya national guidelines because of its benefits for reducing duration and severity of episodes for watery diarrhea,(18) was 1.92 (95% CI: 0.96-3.86; *p*=0.066) for those who demanded amoxicillin, relative to those who demanded albendazole. Though not statistically significant at the 5% level, this difference has a clinical significance since the lower bound of the 95% CI is very close to 1. Despite how zinc is often recommended in addition to ORS to shorten the duration of symptoms, it is not mentioned in the guidelines to be available at private health facilities. Regardless, those who demanded albendazole were 33.0% less likely to receive zinc supplementation (coefficient = -0.148, SE = 0.081, *p*=0.071; Appendix Table B2A, column 8).

With respect to inappropriate medicines, demanding albendazole significantly favors the odds that albendazole is dispensed/prescribed, relative to the visits where the SP demanded amoxicillin (aOR in favor of SPs demanding amoxicillin: 0.06, 95% CI: 0.02-0.22, *p*<0.0001). This translates into a 34.8 percentage point significant increase (SE 0.059, *p*=0.000; Appendix Table B2A, column 13) in whether albendazole was given, compared to 3.1% of SPs who did not demand albendazole receiving it. This effect is similar for whether any antiparasitic is dispensed/prescribed (aOR in favor of SPs demanding amoxicillin: 0.18, 95% CI: 0.07-0.43, *p*=0.0001).

We find higher prescribing/dispensing rates of any antibiotic relative to any antiparasitic (56% vs. 25%, respectively; Table 3) with 21% of visits resulting in both types of drugs being given. For all visits regardless of what the SP demanded, the most frequently given antibiotics were metronidazole (N = 54, 27%), sulfamethoxazole and trimethoprim (N=38, 19%), metronidazole benzoate (N=24, 12%), and amoxicillin (N=19, 10%). We find evidence that demanding amoxicillin has no effect on whether providers dispense/prescribe it (aOR: 1.73, 95% CI: 0.51-5.82, *p*=0.3778) with a similar null finding on whether providers dispense/prescribe any antibiotic (aOR: 0.94, 95% CI: 0.48-1.84, *p*=0.8526) relative to the visits with SPs demanding

For peer review only - http://bmjopen.bmj.com/site/about/guidelines.xhtml

albendazole. Demanding albendazole versus amoxicillin resulted in different types of medicines being dispensed/prescribed at different frequencies across the SP visits (see Appendix Table B3).

Discussion

Main findings

Using the SP method, this study reports the extent to which provider treatment behaviors are influenced by patient demand for treatment, particularly two medicines that are unnecessary, harmful to the community, yet provided as empiric treatment for acute childhood watery diarrhea. We compared the impact of a patient demanding an antibiotic medicine (amoxicillin, which has known public health risks for the individual and community), to an antiparasitic medication (albendazole) which is perceived to be harmless to the individual but also poses a risk to the community.

Our findings do suggest that providers who receive a client demanding amoxicillin are not likely to dispense what is demanded, which is not the case when a patient demands albendazole. Nonetheless, it is worth noting that, irrespective of patient demanding, 56% of the total 200 SP-provider visits carried out in our study were given or prescribed antibiotics, which is consistent with high rates from private facilities in other settings such as India and Tanzania.(24,25,35) However, this proportion is higher than in the public sector in Kenya, as observed in a smaller cross-sectional SP study carried out in purposively sampled health facilities in Nairobi, where 32.5% (95% CI: 20.0-47.5) of 40 SP-provider visits for child diarrhea led to antibiotic prescribing.(22,24) Similar to other observations from African countries including Kenya, top prescribed antibiotics were from the WHO Access group, such as amoxicillin and metronidazole, partly reflecting the lower cost and easier access compared to other antibiotics.

Strengths and weaknesses of the study

Our study has several strengths and weaknesses. First, the SP method is a particular method that requires a one-time visit for services that: do not subject the client to invasive procedures, can only assess tracer health conditions that have been validated for ethical research, and do not require established client services or follow-up visits, such as those related to chronic conditions or other ailments. However, we identified these attributes of the method as favorable conditions to assess the quality of a walk-in outpatient service for child health services.

Second, we cannot compare levels of services without demanding, because by design, the SPs demanded medicines at the end of the visit, though on some occasions the SPs had to demand earlier (e.g., when the provider was discussing treatment or sending the SP to the clinic's pharmacy) to ensure that there was a consistent though standardized narrative for the visit. A future study that seeks to compare demanding outcomes to not demanding is encouraged to implement separate SP visits. Here, it is important to recognize that SPs are not real patients and can behave in a way that confirms the study hypotheses which has been discussed in previous SP studies.(6,7,42) However, if this were the case for our study, the effects would likely be non-differential with respect to the type of medicine being demanded. Instead, an increased rate is only observed after demanding albendazole, and not after demanding amoxicillin.

For peer review only

Third, given that we only examine the interaction between providers and SPs, we do not report on the role of care-seeking behavior and thus interpret findings conditional on patients seeking care. Further, since SPs are not real patients, what was found with SPs may not exactly reflect what happens with real patients, nor are we able to report on how satisfied real patients would have been given these prescription patterns. Similar to discussions in audit studies on discrimination, provider behaviors captured in this study as a response to SP features or trained characteristics may not translate to actual practice behaviors with real patients.(43,44) Our study does not conduct a detection survey to measure the extent of provider suspicion, but other SP studies with detection surveys find very low detection rates (0-5%).(8,24,45) In the study where we based our childhood diarrhea SP case, Daniels et al. (2017) administered a structured questionnaire two weeks after the completion of SP fieldwork in Nairobi, Kenya and found that despite providers having detected SPs in 9 instances, none of these actually matched the study's SP visits. As described earlier, what the SP method allows us to do which other methods cannot is to identify what happens across multiple providers when providers are presented with SPs randomly assigned to demand different inappropriate medicines, with the same presentation otherwise.

Given these limitations, we have scrutinized a few possible channels in our data that are related to either provider behavior or limitations of the method we implemented. Based on the study design, the increase in correct case management for watery diarrhea can only be related to variables where we have captured the outcome at two time points: pre- and post-demanding. Thus, effects of demanding on correct case management is related to advising on ORS or asked to return. It cannot be from dispensing/prescribing ORS or referring to another facility, which are both captured once at the end of the visit. One can imagine that having a pre-demanding and a post-demanding time points alerts us to an issue that the post-demanding environment simply captures more dispensing/prescribing of ORS, and thus higher correct care because it captures all actions after the entire visit has been completed.

Strengths and weaknesses in relation to other studies

This study adds to the literature in several ways. First, we extend the research done on the roles of patients and providers in the patient-provider relationship, in particular, what happens when patients demand inappropriate care and to what extent do demanding patients have an influence on services? Most notably, our findings stand in contrast with Currie et al. (2014)'s SP study in China, which found that providers increased antibiotic use from 55% to 85% when SPs requested antibiotics. Instead we found that demanding an inappropriate antibiotic did not increase its use, but demanding an antiparasitic did in the Kenyan private sector.(7)

Our findings complement what was reported by Lopez et al. (2020), who comparing both provider and patient roles, assessed whether patients' demands influence overprescription and overuse of antimalarials in Mali.(46) With a large sample of real patients randomly assigned different information and malaria treatment subsidies across 60 health facilities in Bamako, the authors found that patients demanding resulted in higher rates of treatment than if providers were in control of dispensing vouchers. They additionally found that for more severe cases, providers were reluctant to provide inappropriate treatment, but that patient driven demand resulted in an excess of treatment for milder cases.

For peer review only - http://bmjopen.bmj.com/site/about/guidelines.xhtml

Our findings also have implications on the literature on overdispensing of antimicrobial therapy and understanding quality of care outcomes that are related to antimicrobial resistance (AMR). Particularly, in Kenya there have been alarms raised about AMR for diarrheal infections and the Government has launched a national AMR policy just before our study was implemented.(47,48) Although our study does not have enough data to address the AMR issue more deeply, we show how demanding an inappropriate medicine can result in higher rates of mismanagement of childhood illnesses than demanding other inappropriate medicines, which has implications for antimicrobial stewardship efforts, training on the consequences of overprescription, and quality improvement interventions. Our study is not able to speak to whether providers were given training on AMR but is able to shed light on how providers seem to be aware that certain medicines are inappropriate for most cases of childhood acute diarrhea. We highlight what happens if a provider gives medicines demanded by a mother or caretaker of a child.

Possible explanations and implications

Providers may be trading off clinical benefits and risks with profits but doing so based on how concrete clinical consequences are with respect to what may be more appropriate for the presenting condition. A future study could examine this more in depth. Other factors likely play a role in determining prescribing practices, including the limited access to diagnostics to rule out conditions that do not require antimicrobial treatment. Further, we caution on extrapolation to other settings where knowledge and training may not be as high, since knowledge on other correct management outcomes appear to be higher in Kenya than in other LMIC settings for both infectious conditions as well as non-communicable diseases.(22,40,49)

In this study, we did not categorize the efficacy or safety of these drugs, since classifying the prescription of the medicines that were demanded in this study as “harmful” may be misleading or lead to misinterpretation. The safety profile of both drugs in terms of side effects is very good, which is reassuring. Both amoxicillin and albendazole are well tolerated even in young children. However, this might provide the false perception of harmlessness which favors the inappropriate use. In this specific context, the threats to public health likely are much greater than those to the individual. The inappropriate use of amoxicillin, though narrow-spectrum and less problematic than other antibiotics, could favor resistance selection amongst commensal and pathogenic bacteria. Similar considerations apply to albendazole, though the consequences of its widespread use are less studied especially in human medicine.

Conclusion

In the setting of private primary care in Kenya, the SP method allowed us to assess providers with the same patient presentation and to causally infer the effects of patient characteristics and actions on quality of care. Most notably, we sought to investigate whether explicitly asking for amoxicillin (an unnecessary antibiotic) or albendazole (an unnecessary antiparasitic used for deworming) had an impact on correct case management and drug prescribing. We find that the provision of inappropriate medicines as one aspect of care quality can be influenced by patients demanding it, but depending on the drug, that may not always be the case. That providers increased the misuse of the antiparasitic deworming medicine but not the antibiotic amoxicillin suggests the need for future research on provider knowledge, awareness, and perceptions of

For peer review only - http://bmjopen.bmj.com/site/about/guidelines.xhtml

profit and individual and community health trade-offs when making prescription decisions after patients demand specific inappropriate medicines.

Competing interests The authors do not have any competing interests to disclose.

Funding This project was funded by a grant from the Bill and Melinda Gates Foundation (Opportunity ID#: OPP1044138). The former UK Department of International Development provided funding for the grant.

Acknowledgments The authors would like to thank Nicole Perales, Rita Cuckovich, Afke Jagar and Innovations for Poverty Action Kenya for their technical and research assistance; to Jishnu Das and Amy Dolinger for their support; Stefano Bertozzi, Jonathan Kolstad, Aprajit Mahajan, and the two reviewers for their comments and feedback; Catherine Wanjiru, John Wanangwe, Jacob Odipo, Daniel Waithaka for their advising; to Andrew Muriithi, Pheliciah Mwachofi, Purity Kimuru, Rodgers Kegode, and Salome Omondi for their superb supervision; and to the SPs for their communication and hard work.

Author Contributions Conceptualization: AK, CB, GS, PG. Funding acquisition: PG, AK. Methodology: AK, PG. Fieldwork supervision: AK. Coding of medicines and tests: AK, GS. Writing ± original draft: AK, CB, GS. Writing ± review & editing: AK, GS, CB, PG.

Data Sharing Statement Individual de-identified interaction data with variables and code needed to re-create the results reported in this article are included and accessible at: https://github.com/kwantify/ahme_demanding/.(41)

For peer review only

References

1. Zanichelli V, Tebano G, Gyssens I, Vlahović-Palčevski V, Monnier A, Benic MS, et al. Patient-related determinants of antibiotic use: a systematic review. Clinical microbiology and infection. 2019;25(1):48–53.

2. Coenen S, Francis N, Kelly M, Hood K, Nuttall J, Little P, et al. Are patient views about antibiotics related to clinician perceptions, management and outcome? A multi-country study in outpatients with acute cough. PloS one. 2013;8(10):e76691.

3. Wagner CE, Prentice JA, Saad-Roy CM, Yang L, Grenfell BT, Levin SA, et al. Economic and behavioral influencers of vaccination and antimicrobial use. Frontiers in Public Health. 2020;8:975.

4. Rodrigues AT, Roque F, Falcão A, Figueiras A, Herdeiro MT. Understanding physician antibiotic prescribing behaviour: a systematic review of qualitative studies. International journal of antimicrobial agents. 2013;41(3):203–12.

5. Chen BK, Gertler PJ, Yang C-Y. Physician ownership of complementary medical services. Journal of Public Economics. 2016;144:27–39.

6. Currie J, Lin W, Zhang W. Patient knowledge and antibiotic abuse: Evidence from an audit study in China. Journal of health economics. 2011;30(5):933–49.

7. Currie J, Lin W, Meng J. Addressing antibiotic abuse in China: An experimental audit study. Journal of development economics. 2014;110:39–51.

8. Kwan A, Daniels B, Bergkvist S, Das V, Pai M, Das J. Use of standardised patients for healthcare quality research in low-and middle-income countries. BMJ global health. 2019;4(5):e001669.

9. Bell M. Antibiotic misuse: a global crisis. JAMA internal medicine. 2014;174(12):1920–1.

10. Wang J, Wang P, Wang X, Zheng Y, Xiao Y. Use and prescription of antibiotics in primary health care settings in China. JAMA internal medicine. 2014;174(12):1914–20.

11. World Health Organization. Antimicrobial resistance [Internet]. 2020 [cited 2020 Oct 13]. Available from: https://www.who.int/news-room/fact-sheets/detail/antimicrobial-resistance

12. Agarwal S, Yewale VN, Dharmapalan D. Antibiotics use and misuse in children: a knowledge, attitude and practice survey of parents in India. Journal of clinical and diagnostic research: JCDR. 2015;9(11):SC21.

13. Labi A-K, Obeng-Nkrumah N, Bjerrum S, Aryee NAA, Ofori-Adjei YA, Yawson AE, et al. Physicians' knowledge, attitudes, and perceptions concerning antibiotic resistance: a survey in a Ghanaian tertiary care hospital. BMC health services research. 2018;18(1):1–12.

For peer review only

14. Adhikari B, Pokharel S, Raut S, Adhikari J, Thapa S, Paudel K, et al. Why do people purchase antibiotics over-the-counter? A qualitative study with patients, clinicians and dispensers in central, eastern and western Nepal. BMJ global health. 2021;6(5):e005829.

15. Greene J, Hibbard JH. Why does patient activation matter? An examination of the relationships between patient activation and health-related outcomes. Journal of general internal medicine. 2012;27(5):520–6.

16. Hibbard JH, Stockard J, Mahoney ER, Tusler M. Development of the Patient Activation Measure (PAM): conceptualizing and measuring activation in patients and consumers. Health services research. 2004;39(4p1):1005–26.

17. Hibbard JH, Greene J. What the evidence shows about patient activation: better health outcomes and care experiences; fewer data on costs. Health affairs. 2013;32(2):207–14.

18. Kenya Ministry of Health. Policy Guidelines for Management of Diarrhoea in Children below Five Years in Kenya. Kenya Ministry of Health; 2014.

19. Mohanan M, Vera-Hernández M, Das V, Giardili S, Goldhaber-Fiebert JD, Rabin TL, et al. The know-do gap in quality of health care for childhood diarrhea and pneumonia in rural India. JAMA pediatrics. 2015;169(4):349–57.

20. Das J, Holla A, Das V, Mohanan M, Tabak D, Chan B. In urban and rural India, a standardized patient study showed low levels of provider training and huge quality gaps. Health affairs. 2012;31(12):2774–84.

21. Sylvia S, Shi Y, Xue H, Tian X, Wang H, Liu Q, et al. Survey using incognito standardized patients shows poor quality care in China's rural clinics. Health policy and planning. 2015;30(3):322–33.

22. Daniels B, Dolinger A, Bedoya G, Rogo K, Goicoechea A, Coarasa J, et al. Use of standardised patients to assess quality of healthcare in Nairobi, Kenya: a pilot, cross-sectional study with international comparisons. BMJ global health. 2017;2(2):e000333.

23. Tielsch JM. The know, do, and quality gaps in international maternal and child health care interventions. JAMA pediatrics. 2015;169(4):313–4.

24. Sulis G, Daniels B, Kwan A, Gandra S, Daftary A, Das J, et al. Antibiotic overuse in the primary health care setting: a secondary data analysis of standardised patient studies from India, China and Kenya. BMJ global health. 2020;5(9):e003393.

25. Sulis G, Adam P, Nafade V, Gore G, Daniels B, Daftary A, et al. Antibiotic prescription practices in primary care in low-and middle-income countries: A systematic review and meta-analysis. PLoS medicine. 2020;17(6):e1003139.

26. Carter E, Bryce J, Perin J, Newby H. Harmful practices in the management of childhood diarrhea in low-and middle-income countries: a systematic review. BMC Public Health. 2015;15(1):1–34.

For peer review only

27. Fink G, D'Acremont V, Leslie HH, Cohen J. Antibiotic exposure among children younger than 5 years in low-income and middle-income countries: a cross-sectional study of nationally representative facility-based and household-based surveys. The Lancet infectious diseases. 2020;20(2):179–87.

28. Doan T, Worden L, Hinterwirth A, Arzika AM, Maliki R, Abdou A, et al. Macrolide and nonmacrolide resistance with mass azithromycin distribution. New England Journal of Medicine. 2020;383(20):1941–50.

29. Rhee C, Aol G, Ouma A, Audi A, Muema S, Auko J, et al. Inappropriate use of antibiotics for childhood diarrhea case management—Kenya, 2009–2016. BMC Public Health. 2019;19(3):1–12.

30. Troeger C, Forouzanfar M, Rao PC, Khalil I, Brown A, Reiner Jr RC, et al. Estimates of global, regional, and national morbidity, mortality, and aetiologies of diarrhoeal diseases: a systematic analysis for the Global Burden of Disease Study 2015. The Lancet Infectious Diseases. 2017;17(9):909–48.

31. Walker CLF, Rudan I, Liu L, Nair H, Theodoratou E, Bhutta ZA, et al. Global burden of childhood pneumonia and diarrhoea. The Lancet. 2013;381(9875):1405–16.

32. World Health Organization. Diarrhoeal disease - Key facts [Internet]. 2017 [cited 2017 May 2]. Available from: https://www.who.int/news-room/fact-sheets/detail/diarrhoeal-disease

33. Mokomane M, Kasvosve I, Melo E de, Pernica JM, Goldfarb DM. The global problem of childhood diarrhoeal diseases: emerging strategies in prevention and management. Therapeutic advances in infectious disease. 2018;5(1):29–43.

34. World Health Organization. Department of Child and Adolescent Health and World Health Organization and UNICEF. Handbook IMCI: integrated management of childhood illness. World Health Organization; 2005.

35. King JJ, Powell-Jackson T, Makungu C, Hargreaves J, Goodman C. How much healthcare is wasted? A cross-sectional study of outpatient overprovision in private-for-profit and faith-based health facilities in Tanzania. Health policy and planning. 2021;36(5):695–706.

36. Flottorp SA, Oxman AD, Krause J, Musila NR, Wensing M, Godycki-Cwirko M, et al. A checklist for identifying determinants of practice: a systematic review and synthesis of frameworks and taxonomies of factors that prevent or enable improvements in healthcare professional practice. Implementation Science. 2013;8(1):1–11.

37. World Health Organization and others. Standards for improving the quality of care for children and young adolescents in health facilities. 2018;

38. Peabody JW, Luck J, Glassman P, Dresselhaus TR, Lee M. Comparison of vignettes, standardized patients, and chart abstraction: a prospective validation study of 3 methods for measuring quality. Jama. 2000;283(13):1715–22.

For peer review only

39. Das J, Hammer J, Leonard K. The quality of medical advice in low-income countries. Journal of Economic Perspectives. 2008;22(2):93–114.

40. Das J, Woskie L, Rajbhandari R, Abbasi K, Jha A. Rethinking assumptions about delivery of healthcare: implications for universal health coverage. BMJ. 2018;361:k1716.

41. Kwan, A. Data from: Do private providers give patients what they demand, even if it is inappropriate? A randomized study utilizing unannounced standardized patients in Kenya. [Internet]. GitHub. 2022. Available from: https://github.com/kwantify/ahme_demanding/

42. Aujla N, Chen Y-F, Samarakoon Y, Wilson A, Grolmusova N, Ayorinde A, et al. Comparing the use of direct observation, standardized patients and exit interviews in low-and middle-income countries: a systematic review of methods of assessing quality of primary care. Health policy and planning. 2021;36(3):341–56.

43. Goldberg PK. Dealer price discrimination in new car purchases: Evidence from the consumer expenditure survey. Journal of Political Economy. 1996;104(3):622–54.

44. Heckman JJ. Detecting discrimination. Journal of economic perspectives. 1998;12(2):101–16.

45. Das J, Kwan A, Daniels B, Satyanarayana S, Subbaraman R, Bergkvist S, et al. Use of standardised patients to assess quality of tuberculosis care: a pilot, cross-sectional study. The Lancet infectious diseases. 2015;15(11):1305–13.

46. Lopez C, Sautmann A, Schaner S. Does Patient Demand Contribute to the Overuse of Prescription Drugs? 2020;

47. Global Antibiotic Resistance Partnership—Kenya Working Group,. Situation Analysis and Recommendations: Antibiotic Use and Resistance in Kenya [Internet]. Washington, DC and New Delhi: Center for Disease Dynamics, Economics & Policy; 2011 Aug [cited 2022 Jan 31]. Available from: https://cddep.org/wp-content/uploads/2017/08/garp-kenya_sa.pdf

48. Republic of Kenya,. National Policy on Prevention and Containment of Antimicrobial Resistance [Internet]. Nairobi, Kenya: Government of Kenya; 2017 Apr [cited 2022 Jan 31]. Available from: https://www.health.go.ke/wp-content/uploads/2017/04/Kenya-AMR-Containment-Policy-_Final_April.pdf

49. Di Giorgio L, Evans DK, Lindelow M, Nguyen SN, Svensson J, Wane W, et al. Analysis of clinical knowledge, absenteeism and availability of resources for maternal and child health: a cross-sectional quality of care study in 10 African countries. BMJ global health. 2020;5(12):e003377.

For peer review only - http://bmjopen.bmj.com/site/about/guidelines.xhtml

Figure 1. Clinic sample and SP randomized study design

[Insert Figure 1]

For peer review only

Table 1. Description of childhood diarrhea standardized patient case scenario and main outcomes

Case	Case description	SP experiments varying patient characteristics	Outcomes
Childhood diarrhea	A 28-year-old mother comes to the clinic with a 1.5-year-old child at home sick with acute watery diarrhea. The child is a little hot and has passed approximately 6-7 stools in the last two days.	Experiment 1: Demanding albendazole<br><br>Experiment 2: Demanding amoxicillin	Correct case management (=1): Any one of the following were done by the provider:<br>gave ORS, advised on ORS,<br>referred elsewhere,<br>asked to return to clinic for any reason<br><br>Any unnecessary medicines (=1): Any medicines given excluding ORS, zinc

For peer review only

Table 2. Balance across characteristics of clinics assigned albendazole vs. amoxicillin demanding experiment.

	Clinics Assigned to Receive an SP Demanding Albendazole, n = 102			Clinics Assigned to Receive an SP Demanding Amoxicillin, n = 98			
	N	*Mean*	*95% CI*	*N*	*Mean*	*95% CI*	*p-value*
Hours open per week	94	101.86	(91.77 - 111.96)	88	94.91	(85.01 - 104.81)	0.337
Average of hours open per day	94	14.85	(13.46 - 16.24)	88	14.12	(12.79 - 15.45)	0.460
Clinic is NHIF empaneled	94	0.30	(0.21 - 0.39)	88	0.32	(0.22 - 0.42)	0.768
Number of clients	94	466.86	(353.84 - 579.89)	88	525.27	(354.13 - 696.42)	0.573
Data missing	102	0.08	(0.03 - 0.13)	98	0.10	(0.04 - 0.16)	0.562
Count of total staff	95	3.97	(3.30 - 4.64)	88	3.99	(3.38 - 4.60)	0.965
Count of clinical staff (doctors and nurses)	95	2.23	(1.81 - 2.65)	88	2.20	(1.86 - 2.55)	0.923
Data missing	102	0.07	(0.02 - 0.12)	98	0.10	(0.04 - 0.16)	0.400
Facility has community health workers	94	0.40	(0.31 - 0.50)	88	0.33	(0.23 - 0.43)	0.299
Total revenues (USD)	92	4,534.70	(2,493.57 - 6,575.83)	85	3,621.63	(2,106.29 - 5,136.97)	0.488
Total profits (USD)	91	1,487.57	(570.25 - 2,404.89)	83	-2,665.31	(-8,670.93 - 3,340.31)	0.163
Total expenditures (USD)	92	3,057.43	(1,548.01 - 4,566.85)	85	6,175.33	(-831.09 - 13,181.75)	0.378
Services provided at clinic							
Facility provides any curative services	94	0.97	(0.93 - 1.00)	88	0.95	(0.91 - 1.00)	0.637
Antenatal care	94	0.69	(0.60 - 0.79)	88	0.68	(0.58 - 0.78)	0.889
Cervical cancer screening	94	0.49	(0.39 - 0.59)	88	0.48	(0.37 - 0.58)	0.871
Delivery	94	0.40	(0.31 - 0.50)	88	0.48	(0.37 - 0.58)	0.324
Dental services	94	0.16	(0.09 - 0.23)	88	0.17	(0.09 - 0.25)	0.844
Family Planning	94	0.98	(0.95 - 1.01)	88	0.99	(0.97 - 1.01)	0.602
Imaging services (X-ray, Ultrasound)	94	0.14	(0.07 - 0.21)	88	0.15	(0.07 - 0.22)	0.857
Immunizations visit	94	0.37	(0.27 - 0.47)	88	0.45	(0.35 - 0.56)	0.263
In-patient services	94	0.26	(0.17 - 0.34)	88	0.26	(0.17 - 0.35)	0.926
Laboratory services	94	0.93	(0.87 - 0.98)	88	0.92	(0.86 - 0.98)	0.898
Malaria testing / treatment	94	0.96	(0.92 - 1.00)	88	0.94	(0.90 - 0.99)	0.659
Optical services	94	0.09	(0.03 - 0.14)	88	0.10	(0.04 - 0.17)	0.693
Pharmacy services	94	0.32	(0.22 - 0.41)	88	0.42	(0.32 - 0.52)	0.158
Post-natal care	94	0.55	(0.45 - 0.65)	88	0.58	(0.48 - 0.68)	0.722
Respiratory tract infections	94	0.98	(0.95 - 1.01)	88	1.00	(1.00 - 1.00)	0.171
Well-baby visit	94	0.62	(0.52 - 0.72)	88	0.62	(0.52 - 0.73)	0.912
Services - data missing	102	0.08	(0.03 - 0.13)	98	0.10	(0.04 - 0.16)	0.562

For peer review only - http://bmjopen.bmj.com/site/about/guidelines.xhtml

Note. Number of observations refers to the number of clinics in the sample visited by SPs. The data source for this table does not have data available for all 200 private clinics in the sample. Data missing varies by type of variable – see “data missing” for percentage of clinics where data is missing for number of clients, count of staff, and services provided at the clinic. NHIF = National Hospital Insurance Fund. AHME program refers to the African for Health Markets for Equity (AHME) program. USD is United States dollar.

For peer review only

Table 3. Summary statistics of SP visits

	(1) Pooled SP Visits, n = 200		(2) SP Visits Demanding Albendazole, n = 102		(3) SP Visits Demanding Amoxicillin, n = 98		(3)-(2) *difference in means t-test p*-value
	N	Mean	N	Mean	N	Mean	
**Provider characteristics**							
Provider is female	196	0.38	99	0.35	97	0.41	0.399
Provider age group	200		102		98		
Under 30	33	0.17	18	0.18	15	0.15	
Between 30 and 50	114	0.57	59	0.58	55	0.56	
Above 50	42	0.21	18	0.18	24	0.24	
Missing data	11	0.06	7	0.07	4	0.04	
Provider qualification	200		102		98		
Medical doctor or Clinical Officer	66	0.33	36	0.35	30	0.31	
Nurse or Midwife	60	0.30	31	0.30	29	0.30	
Other Staff	16	0.08	8	0.08	8	0.08	
Unknown or Missing data	58	0.29	27	0.26	31	0.32	
Knowledge of correct management							
Diarrhea	140	0.90	72	0.92	68	0.88	0.502
**Visit characteristics**							
Number of patients waiting when SP arrived	200	1.55	102	1.25	98	1.87	0.122
Minutes spent with provider	200	6.65	102	6.21	98	7.10	0.089
Number of history questions asked (post)	200	4.46	102	4.41	98	4.50	0.820
Correct diagnosis or suspicion (post)	200	0.15	102	0.11	98	0.19	0.089
Correct case management (post)	200	0.75	102	0.75	98	0.76	0.871
Any lab tests ordered (post)	200	0.13	102	0.16	98	0.10	0.251
Total lab tests ordered (post)	200	0.26	102	0.29	98	0.21	0.408
Any unnecessary lab tests (post)	200	0.10	102	0.08	98	0.12	0.302
Total unnecessary lab tests (post)	200	0.14	102	0.16	98	0.12	0.519
Number of medicines	200	2.38	102	2.40	98	2.35	0.845
Number of non-efficacious medicines	200	1.63	102	1.75	98	1.50	0.260
Dispensed/ prescribed: Albendazole	200	0.19	102	0.34	98	0.03	0.000
Dispensed/ prescribed: Antiparasitics	200	0.25	102	0.35	98	0.13	0.000
Dispensed/ prescribed: Amoxicillin	200	0.10	102	0.08	98	0.11	0.417
Dispensed/ prescribed: Antibiotics	200	0.56	102	0.56	98	0.55	0.912
Dispensed/ prescribed: Antibiotics & Antiparasitics	200	0.21	102	0.28	98	0.12	0.004

For peer review only - http://bmjopen.bmj.com/site/about/guidelines.xhtml

Asked to return (post)	200	0.31	102	0.32	98	0.29	0.564
Referred elsewhere	200	0.06	102	0.07	98	0.05	0.602
Providers did good job explaining	189	0.76	95	0.74	94	0.79	0.419

Note. Table displays summary statistics (N, mean) for all SP visits pooled (column 1), all SP visits assigned to demand albendazole (column 2), and all SP visits assigned to demand amoxicillin (column 3). Statistics with "(post)" are post-demanding measures; all others are one time at the end of the visit. All summary statistics except knowledge of correct management for diarrhea come from SP surveys. Knowledge of correct management is defined in the same way as correct case management and come from a vignette administered in the provider survey. Vignette data are matched to SP data for each SP visit by provider seen by SP or a replacement for the sampled provider. Provider age group is the estimated age group as perceived by the SP.

For peer review only

Figure 2. Differences in quality of care by standardized patients demanding albendazole vs. amoxicillin

[Insert Figure 2]

Note. The chart illustrates estimated differences by the SP demanding experiment across quality-of-care outcomes. Odds ratios are estimated controlling for SP fixed effects. All variables are binary outcomes.

For peer review only

SP Survey: Childhood Diarrhea

Enrollment

232 Private Clinics

Randomized: 232 visits at 232 clinics

Allocation

Demanding albendazole
Assigned: 116 interactions at 116 clinics

Demanding amoxicillin
Assigned: 116 interactions at 116 clinics

Data Collection

Successfully visited: 102 interactions at 102 clinics

Ineligible/ Excluded or Unsuccessfully visited: 14 interactions at 14 clinics

Successfully visited: 98 interactions at 98 clinics

Ineligible/ Excluded or Unsuccessfully visited: 18 interactions at 18 clinics

Analysis

Analyzed:
102 interactions at 102 clinics
(0 excluded from analysis)

Analyzed:
98 interactions at 98 clinics
(0 excluded from analysis)

Ineligible/ Excluded (12 interactions at 12 clinics)

- Ineligible: 4 clinics
  - 2 clinics receive firm employees only
  - 1 fistula clinic
  - 1 eye clinic
- Excluded: 8 clinics
  - 1 located in area with security concerns
  - 7 did not consent to participate in related surveys (outside scope of this study)

Unsuccessfully visited (20 interactions at 20 clinics)

- Permanently closed: 14 clinics
- Other: 6 clinics turned away SP upon arrival

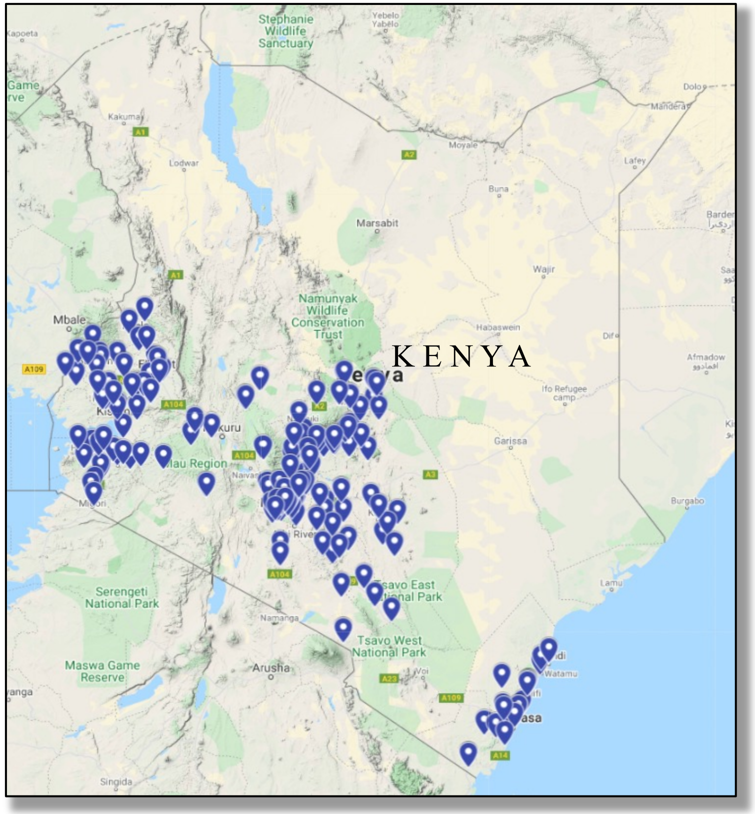

For peer review only - http://bmjopen.bmj.com/site/about/guidelines.xhtml

	Odds Ratio	[95% Confidence]	P-value
Correct case management	0.76	[0.36 , 1.64]	0.4894
Advised on ORS	1.21	[0.61 , 2.37]	0.585
Gave or advised on ORS	1.47	[0.75 , 2.88]	0.2665
Asked to return or referred	0.9	[0.44 , 1.84]	0.7643
Any lab tests ordered	0.41	[0.14 , 1.17]	0.0949
Any unnecessary lab tests	1.22	[0.36 , 4.08]	0.7491
Dispensed/ prescribed: ORS	1.37	[0.69 , 2.74]	0.3655
Dispensed/ prescribed: Zinc	1.92	[0.96 , 3.86]	0.066
Dispensed/ prescribed: Albendazole	0.06	[0.02 , 0.22]	<0.0001
Dispensed/ prescribed: Antiparasitics	0.18	[0.07 , 0.43]	0.0001
Dispensed/ prescribed: Amoxicillin	1.73	[0.51 , 5.82]	0.3778
Dispensed/ prescribed: Antibiotics	0.94	[0.48 , 1.84]	0.8526
Dispensed/ prescribed: Antibiotics & Antiparasitics	0.23	[0.09 , 0.58]	0.0019

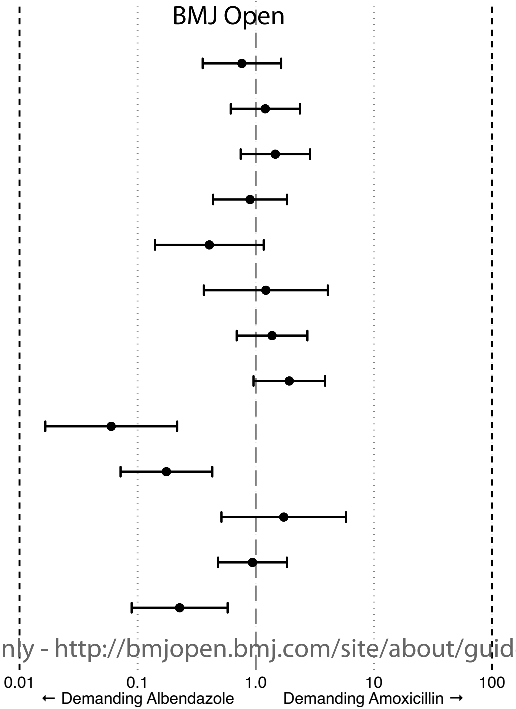

For peer review only - http://bmjopen.bmj.com/site/about/guidelines.xhtml

SUPPLEMENTAL APPENDICES

Supplement to:

Do private providers give patients what they demand, even if it is inappropriate? A randomized study utilizing unannounced standardized patients in Kenya

Ada Kwan[1,2], Claire E. Boone[2], Giorgia Sulis[3], and Paul J. Gertler[4]

[1]Division of Pulmonary and Critical Care Medicine, University of California San Francisco School of Medicine, San Francisco, CA, United States
[2]Division of Health Policy and Management, University of California, Berkeley School of Public Health, Berkeley, CA, United States
[3]Department of Epidemiology, Biostatistics, and Occupational Health, McGill University, Montreal, Canada
[4]Haas School of Business, University of California, Berkeley, Berkeley, CA, United States

For peer review only

For peer review only - http://bmjopen.bmj.com/site/about/guidelines.xhtml

APPENDIX A: SUPPLEMENTAL METHODS ON STUDY CONTEXT, SP METHOD, AND ETHICS

For peer review only

A1 Childhood diarrhea case management

Appendix Figure A1. Kenya national guidelines for health facility case management of childhood diarrhea

Who

- Health care providers in public, private, and faith-based facilities should implement outlined Ministry of Health guidelines in management of diarrhea in children under five years old.
- All healthcare providers including doctors, nurses, clinical officers, nutritionists, pharmacists, and public health officers should be trained on proper case management in children under five years.

Minimum package for facility case management

- All facilities should have a functional ORT corner for immediate start of on-site rehydration of children with diarrhea.
- All children with diarrhea should be given ORS and zinc combination therapy as soon as diarrhea has started. Combination therapy of ORS and zinc tablets (Co-pack) is available at the following places: public health facilities, community health units, pharmacies (public / private), and retail shops.

Product information

- ORS: benefits include reduction of stool output, reduction of vomiting incidence, replacement of sodium bicarbonate and potassium, replacement of admission burden and IV fluid treatment. Low osmolarity ORS; local production and procurement; half liter sachet.
- Zinc: benefits include reduction of duration and severity of episodes, lowering of diarrhea incidence in the following 2-3 months, reduction of pneumonia incidence, improvement of cellular intestinal immune and epithelial functioning, and increased functioning of the immune system. Dispersible tablets (20 mg) with local production and procurement.

Caregiver responsibilities

- On leaving a health facility for home, a caregiver shall be given one co-pack containing 4 sachets of ORS and 10 tablets of zinc and will receive guidance on home management.
- In addition, the caregiver should be advised to return to the health facility within two days if diarrhea continues or immediately if the child: is not able to drink or breastfeed, becomes sicker, is vomiting everything, is drinking poorly, or has blood in stool.

Other drugs should be given only when deemed absolutely necessary during the management of diarrheal diseases. General guidelines are:

- Guidelines also state that all children with diarrhea shall be given an appropriate dose of Vitamin A at the facility if they have not received a dose in the last one month. IV should be used in children with the following conditions: severe dehydration (as per Integrated Management of Childhood Illnesses guidelines); severe, profuse, repeated diarrhea; persistent vomiting; inability to drink; abdominal distention; paralytic ileus; glucose malabsorption; shock. The fluid of choice shall be Ringer's lactate. In cases where it is not available, normal saline can be used. Every effort should be made to replace IV therapy with oral therapy as soon as the child is able to drink. Where the child cannot get IV therapy at once, ORS solution shall be given by nasogastric tube or orally until IV therapy is started.
- Management of dehydration in children with severe malnutrition: the fluid of choice is rehydration solution for malnutrition. IV fluids should not be used unless the child is in shock.
- Antidiarrheal and anti-emetics shall not be used. None have any proven practical value, and some are dangerous.
- Antibiotics shall be used only for suspected or proven dysentery and cholera cases. In diarrhea of any other etiology antibiotics are of no practical value and shall not be given.
- Antiprotozoal drugs shall only be used for the treatment of amoebiasis and giardiasis.
- For cholera, erythromycin (1st line) and chloramphenicol (2nd line). For shigella dysentery, ciprofloxacin. For amoebiasis, metronidazole. For giardiasis, metronidazole

Drugs, equipment, and supplies

- A commodity management system, which enhances visibility of procured drugs, equipment and supplies, should be promoted to strengthen the availability of diarrhea management commodities. The following drugs, equipment and supplies should be available at all times.
- Drugs: zinc and ORS, erythromycin, chloramphenicol, ciprofloxacin, Vitamin A, metronidazole, and intravenous fluids such as Ringer's lactate, normal saline and ReSoMal. Oral rehydration equipment and supplies as per the ORT corner guidelines.

For peer review only - http://bmjopen.bmj.com/site/about/guidelines.xhtml

A2 African Health Markets for Equity (AHME) Program and Evaluation

Details of the AHME intervention are provided where necessary for this study; however, the impact of the AHME intervention is not the purpose of this study. Across Kenya between 2013-2018, the AHME intervention was intended to "use National Health Insurance to link supply (private providers) with demand (clients) in order to shift health markets toward providing quality health care to low-income patients" . In Kenya, 56% of the population earns US$1 or less a day, and a third of the poor who are sick do not seek care, according to National Health Accounts data. To improve this situation, the AHME intervention is a mix of demand- and supply-side interventions implemented by non-governmental organizations in the context of a new scheme within the National Health Insurance Fund (NHIF). The demand- and supply-side interventions include: National Social Health Insurance scheme by Ministry of Health; SafeCare quality improvement by PharmAccess (similar to that described in Dunsch et al. [12]); Social franchising by Marie Stopes Kenya (MSK) and Population Services Kenya (PSK). The AHME impact evaluation's main aim is to assess the impacts and cost-effectiveness of the AHME intervention. Additionally, the evaluation aims to assess AHME's impact on several dimensions of quality including: (1) SafeCare's 680 measures of quality used in health facility assessments and quality improvement plans, (2) perceived quality measured by previous patients through a household survey, (3) perceived quality by existing patients through exit interviews, (4) provider knowledge through provider vignettes, (5) provider practice through SPs, and (6) patient safety outcomes through direct observation techniques and SPs.

A2.1. Experimental Design of the AHME Impact Evaluation

This section describes the AHME impact evaluation experimental design and clinic selection. Figure 1 shows a map of the clinics across Kenya alongside the process of clinic selection, which is described in detail as six steps below.

<u>Step 1. Clinics in Kenya listed.</u> Before the AHME program began in 2012, we mapped all clinics in 35 of Kenya's 47 counties with the goal of randomizing clinics eligible for the AHME program into treatment and control groups. Because there was no pre-existing list of private clinics at the time, clinics were first identified for mapping using four sources of information: (1) official government list of private clinics in the country, (2) clinics belonging to a major professional health associate (e.g., the Kenya Nurse and Midwives Association), (3) clinics that the AHME implementation partners suggested should be visited, and (4) additional clinics identified by evaluation teams in the field during the mapping process, but not included on any of the above lists. Government clinics (public clinics and hospitals), faith-based clinics (identified by clinic name), and clinics that were identified as franchised (by franchise branding on clinic exterior) were removed from the sampling frame generated from the aforementioned information sources.

<u>Step 2. Baseline clinic survey administered.</u> A pre-screening and baseline survey among remaining clinics was administered to remaining clinics. The purpose of this was to exclude clinics that the implementing partners indicated were not eligible for franchise services or the AHME set of interventions. Clinics that met the basic eligibility criteria still varied in their "level of eligibility" based on their existing capacity and suitability for franchising services and AHME interventions. Using additional criteria, created in a collaborative manner with MSK and PSK, clinics were further categorized into groups based on how likely they were to be eligible ("eligibility tiers") using data collected through the baseline survey instrument.

<u>Step 3. Randomization.</u> After clinics were categorized by eligibility tier, the research team conducted a stratified randomization of eligible clinics. For the randomization process, clinics were grouped into their

For peer review only - http://bmjopen.bmj.com/site/about/guidelines.xhtml

eligibility tiers within a county based on partner-provided criteria and data from the baseline survey, randomly ordered within strata (groups within which randomization would occur), and then randomly assigned to treatment (eligible to be offered AHME franchising immediately) or control (not eligible to be offered AHME until the completion of the study).

Steps 4 & 5. Clinics screened based on survey & site visit screening. After randomization procedures were completed, we provided MSK and PSK with partner-specific recruitment lists indicating the order in which clinics on their lists were to be approached for screening ("sensitization") and recruitment. Once randomization procedures had been completed, MSK and PSK began engaging clinics on their lists and proceeded with their respective recruitment procedures in October 2013. The second round of screening and recruitment by the partners served to identify clinics that were eligible for franchising and AHME interventions in the treatment arm. Eligible clinics that were invited to join either franchise ("ever franchised") were considered part of the evaluation sample in the treatment arm. Consistent with the intent-to-treat (ITT) assumptions applied in this study, these ever-franchised clinics were considered part of the final evaluation sample regardless of whether they completed the franchise enrollment process or maintained their franchise enrollment status for the entirety of the study period (for any reason).

Step 6. Final clinic sample for AHME impact evaluation. The final AHME evaluation samples were identified over the course of recruitment and honing activities. In total, 232 clinics were identified for the final evaluation sample (treatment clinics: N = 123; control clinics: N = 109). In September 2016, baseline data collection, including baseline household and client exit interviews, was completed for all AHME clinics.

The SP experiments were randomly assigned independent of the AHME treatment assignment. The table below shows the balance of AHME assignment across the SP demanding experiment assignments for our analytic sample. We include a AHME treatment indicator for analyses based on the clinic assignment to the AHME treatment or control group.

Appendix Table A1. Difference in means of AHME assignment by SP demanding experiment.

	**Clinics Assigned to Receive an SP Demanding Albendazole, n = 102**			**Clinics Assigned to Receive an SP Demanding Amoxicillin, n = 98**			
	N	*Mean*	*95% CI*	*N*	*Mean*	*95% CI*	*p-value*
Randomly assigned to receive AHME program	102	0.60	(0.50 - 0.69)	98	0.46	(0.36 - 0.56)	0.050

For peer review only - http://bmjopen.bmj.com/site/about/guidelines.xhtml

A3 Standardized Patient Recruitment, Training, and Pilot

We implemented two main survey methodologies: SPs and provider vignettes, which minimize bias in assessing provider practice and provider knowledge, respectively. For our SP data, we define visits as visits where the SP visited the clinic during operating hours and interacted with clinic staff, similar to an actual client presenting with similar conditions.

Other methods to assess quality of care have certain limitations that do not make them ideal for answering our research questions. Health and medical record data often do not exist in LMIC settings and when they do, they suffer from poor data quality. Direct observation is biased by the Hawthorne Effect. Patient exit interviews represent different patient sorting and patient mixes across clinics, and not only do clients not always understand medical jargon, but it is difficult to know precisely what medical condition the client has. Vignettes excel at assessing provider knowledge, but as for practice, vignettes are subject to social desirability bias and differ largely from practice measures (Kwan et al. 2019). For example, the "know-do gap" is a well-documented phenomenon in the literature referring to the difference between provider knowledge and provider practice (Das et al. 2015; Mohanan et al. 2015).

A3.1 SP Cases

We implemented our SP study based on protocol from a previous SP study conducted in Nairobi, Kenya (Daniels et al. 2017; Kwan et al. 2019). A technical advisory group consisting of four Kenyan clinicians advised our team on case development, and all hired SPs participated in developing standardized narratives (e.g., name, age, family situation, living situation, etc.) for the SP case during training. The technical advisory group participated in SP training and advised on outcome measures for each case.

The childhood diarrhea case scenario for SPs were adapted from a pilot conducted in 2014 in Nairobi, Kenya. The case scenario developed for this study has two parts: (1) the SP narrative designed with the technical advisory group and the SP recruits during training and (2) the corresponding SP exit survey. The SP narrative describes the social milieu of the presenting caregiver as well as the situation that motivates the caregiver to access health care services for their child who is sick at home with diarrhea on the day of the visit. Figure A3 shows the case scenario narrative. Figure A4 shows the case scenario's attire for presenting at each clinic, alongside the opening statement, and some history questions to which the SPs are trained to provide pre-scripted responses.

For peer review only - http://bmjopen.bmj.com/site/about/guidelines.xhtml

Appendix Figure A3. Childhood diarrhea case scenario narrative

SP NARRATIVE — AHME (adapted from Nairobi)

Standardized Case 1: Watery Diarrhoea

JOSEPHINE

Josephine is 28 years old and a merchandiser in Nairobi. Today she decided to go home earlier than usual because she left her daughter Diana feeling unwell in the house. Diana has been unwell for the past two days and Josephine was really getting worried about her. Since then she had diarrhoea six or seven times and was crying more than usual. As Josephine was making her way home, she received a call from her niece, informing her that Diana's diarrhoea had worsened again. Her husband is away travelling.

Diana is one and a half years old. Lately Josephine has tried to observe cleanliness in the house. But recently there has been some water shortage due to a burst city council pipe. She buys 2-3 20-liter Jelicans of water[1] per day and divides it for all her chores. She stores the drinking water in a five-liter plastic can. She boils it when she has some paraffin to spare after making the family meals. There is no provision for drainage system, and they have one toilet that is shared among many households within the plot. There is also no system for garbage collection, and it is always heaped just behind her house. (Josephine lives in a small village. She gets water from a well/river nearby. She stores her drinking water in a small pot or a jelican. She boils it when she has some paraffin/wood to spare after making the family meals. There is no provision for a drainage system, and they have one toilet that is shared among many households.)

Diana is still breastfeeding, has had all of her immunizations and has been a healthy baby. Lately, she has not been her usual self, and since last night she is having several bouts of watery stools – sometimes they have mucus and stickiness. They did not smell particularly foul though. She seemed a little weak and tired but was still playful. Diana's body was a little hot. She has also been crying more than usual, and it seemed she had some tummy ache. She was vomiting a little. She lost her appetite but was drinking lots of water. Josephine had prepared ORS before she left for work for her to be given during the day. On hearing of the many episodes of diarrhoea that Diana has been having, Josephine was worried. She decided to visit a nearby clinic on her way home.

only

For peer review only - http://bmjopen.bmj.com/site/about/guidelines.xhtml

Appendix Figure A4. Case scenario attire, opening statement, and sample history question responses

SP NARRATIVE **AHME (adapted from Nairobi)**

Josephine's dress:

1. Generally, Josephine is a very simple woman.
2. Wears smart and casual clothes, which are not expensive.
3. She doesn't wear excessive make-up, and most times she does not wear any at all.
4. She puts on simple doll shoes or rubber shoes and small earrings or studs.
5. She carries a very simple handbag sometimes a shopping bag and a kikoi/kanga.
6. In the coast, Josephine wears a long dress/skirt, Dera or a Buibui. In some parts of Homabay and towards Kisii, she does not wear trousers at all. She only wears long skirts.

Opening statement:

<u>My child has been having diarrhea.</u>
Kiswahli: Mtoto wangu ana hara/endesha.
Taveta: Dakitari mwana wangu efwaka
Rabai: Dakitari mwanangu yunahara
Luhya: Omwana wanje anyalala
Kamba: Ndakitali mwana wakwa nukwitua
Meru: Kana gakwa igakwatwa/ mwana okwa nakwarwa
Kalenjin: Taktari mondoe moet lakwenyun
Maasai: Nkitari, keloito (e)nkeraiai (e)nkoshoke
Luo: Daktari, nyathina diewo
Kikuyu: Mwana wakwa niaraharwo
Taita: Dakitari mwana wapowawefwaya
Kisii: Omwana one agosaa
Embu: Ndakitare mwana wakwa nearavarwa

History questions asked by the provider and their answers:

1. Q: How old is the child?
   Mwanao ana umri gani?
   A: 1 1/2 years old.
   Mwaka mmoja unusi.

2. Q: How many times has she passed stools?
   Amehara mara ngapi?
   A: Many times.
   Mara nyingi.

3. Q: How many times in the last 24 hours?
   A: Maybe 6 or 7 times in the last two days.

4. Q: For how many days has she had this?
   Kwa muda wa siku ngapi amekuwa hivi?
   A: Two days ago but it wosened today.
   Siku mbili iliyopita lakini ilimzidia mchana.

For peer review only - http://bmjopen.bmj.com/site/about/guidelines.xhtml

A3.2 SP Recruitment and Training

Figure A5 shows the SP training agenda. SPs were extensively trained in risk mitigation sessions to avoid injections, taking medicine on the spot, unsafe and unsterile needles.

Appendix Figure A5. SP Training Agenda

SP TRAINING AGENDA

	Week 1			
	15-Jan-19 TUESDAY	16-Jan-19 WEDNESDAY	17-Jan-19 THURSDAY	18-Jan-19 FRIDAY
08.30 - 9:15	Registration and Introduction	Introduction to Group Work	Recap and presentation from each group	Recap and presentation from each group
09:15-10:30	Introduction to IPAK/ Admin	Review of SP cases in groups	Group work: Script and Narrative Development	Group reenactment of scripts using risk mitigation strategies
10:30 - 10:45	TEA BREAK	TEA BREAK	TEA BREAK	TEA BREAK
10:45 - 13:00	Introduction to IPAK/Admin<br>Introduction to SP cases	Review of SP cases in groups	Group reenactment of scripts and SP cases	Introduction to Exit Questionnaire
13:00 -14.00	LUNCH	LUNCH	LUNCH	LUNCH
14:00-15:00	Introduction to SP study	Group work: Script and Narrative Development	Risk Mitigation Strategies	Exit Questionnaire
15:00 - 16:00	Group SP into cases	Group work: Script and Narrative Development	Risk Mitigation Strategies	Exit Questionnaire
16:30 - 17:00	TEA BREAK	TEA BREAK	TEA BREAK	TEA BREAK

	Week 2				
	21-Jan-19 MONDAY	22-Jan-19 TUESDAY	23-Jan-19 WEDNESDAY	24-Jan-19 THURSDAY	25-Jan-19 FRIDAY
08.30 - 9:15	Exit Questionnaire	Mock interviews	Mock interviews for SP cases with practice recall questions	Clinicians Assessment of the SP/ mock interviews	Recap and presentation from each group
09:15-10:30					Mock interviews
10:30 - 10:45	TEA BREAK	TEA BREAK	TEA BREAK	TEA BREAK	TEA BREAK
10:45 - 13:00	Exit Questionnaire	Mock interviews	Mock interviews for SP cases with practice recall questions	Clinicians Assessment of the SP/ mock interviews	Mock interviews
13:00 -14.00	LUNCH	LUNCH	LUNCH	LUNCH	LUNCH
14:00-15:00	Introduction to Mock Interviews	Risk Mitigation Strategies	Mock interviews for SP cases with improvisation questions practice	Clinicians Assessment of the SP/ mock interviews	Mock interviews to practice recall questions
15:00 - 16:00	Mock interviews	Mock interviews	Mock interviews for SP cases with improvisation questions practice	Clinicians Assessment of the SP/ mock interviews	Mock interviews to practice recall questions
16:30 - 17:00	TEA BREAK	TEA BREAK	TEA BREAK	TEA BREAK	TEA BREAK

	Week 3				
	28-Jan-19 MONDAY	29-Jan-19 TUESDAY	30-Jan-19 WEDNESDAY	31-Jan-19 THURSDAY	01-Feb-19 FRIDAY
08.30 - 9:15	Risk Mitigation Strategies	Dry runs for the team	Dry runs for the team	Dry runs for the team	Final debriefing of the team
09:15-10:30					
10:30 - 10:45	TEA BREAK	TEA BREAK	TEA BREAK	TEA BREAK	TEA BREAK
10:45 - 13:00	Mock interviews to practice recall questions	Dry runs for the team	Dry runs for the team	Dry runs for the team	Screening of SPs by clinicians
13:00 -14.00	LUNCH	LUNCH	LUNCH	LUNCH	LUNCH
14:00-15:00	Mock interviews to practice recall and improvisation questions	Debriefing of the teams after dry runs	Debriefing of the teams after dry runs	Debriefing of the teams after dry runs	AX24 Last round of mock interviews practice recall and improvisation questions
15:00 - 16:00	Mock interviews to practice recall and improvisation questions	Debriefing of the teams after dry runs	Debriefing of the teams after dry runs	Debriefing of the teams after dry runs	Last round of mock interviews practice recall and improvisation questions
16:30 - 17:00	TEA BREAK	TEA BREAK	TEA BREAK	TEA BREAK	TEA BREAK

A3.3 SP Pilot and Demanding Experiment

Between February 5-15, 2019, the SPs piloted in Nairobi, and some teams also traveled out to three different areas in Kenya to ensure that we understood whether the experiments for the case needed to be adapted for different regions (since the clinic sample was spread across the country).

Given the experience during the pilot, we designed experiments for demanding unnecessary medicines. Figure A6 shows the case scenario narratives with scripts for the two experiments: demanding amoxicillin and demanding albendazole.

After the pilot and between fieldwork, the supervisors conducted refresher trainings in the classroom on the cases and did quality checks on the programmed SP exit questionnaire. Throughout fieldwork, the supervisors also conducted sessions where the case and experiments were reviewed again as a team to ensure there was no evolution of presentation in any given SP.

For peer review only - http://bmjopen.bmj.com/site/about/guidelines.xhtml

Figure A6. Case Scenario Scripts for Demanding Unnecessary Medicine Experiments

Experiments for Standardized Case 1: Watery Diarrhoea

Only conduct the following experiments if assigned by your supervisor.

Experiment 1. Expressing Serious Concern and Demanding Unnecessary Drugs

Josephine goes to the facility, presents her case and on occasions when the provider refuses to give her anything/treat the baby, she tells him/her that she is really worried about her baby's condition and asks to be given (a) *dawa ya minyoo* (ABZ deworming medicines) or (b) Amoxyl (amoxicillin) for babies.

(a) **Demanding dawa ya minyoo (concern, abz):**
When assigned to this experiment, the SP presents worried and remembers another time when her baby was not feeling well and *dawa ya minyoo* calmed the baby. She wants the provider's advice and help in this concerning situation (otherwise she would just go to the chemist), but she is also convinced by her comfort that *dawa ya minyoo* will help.

At three possible moments when appropriate, the SP assigned this experiment can express the desire for *dawa ya minyoo*: (i) when the provider is writing a prescription or about to dispense drugs, (ii) when the doctor asks what the patient wants, or (iii) at the end of the interaction and if the provider hasn't given *dawa ya minyoo* (ABZ) yet, the SP stands up and seem as if she wants to leave then turn back and says to the doctor in a pleading tone:

"But doctor I'm really worried… Can you give me *dawa ya minyoo* for my baby? The last time she was sick, it helped."

(b) **Demanding Amoxyl (concern, amoxyl):**
When assigned to this experiment, the SP presents worried and remembers another time when her baby was not feeling well and *Amoxyl* calmed the baby. She wants the provider's advice and help in this concerning situation (otherwise she would just go to the chemist), but she is also convinced by her comfort that *Amoxyl* will help.

At three possible moments when appropriate, the SP assigned this experiment can express the desire for *Amoxyl*: (i) when the provider is writing a prescription or about to dispense drugs, (ii) when the doctor asks what the patient wants, or (iii) at the end of the interaction and if the provider hasn't given *dawa ya minyoo* (ABZ) yet, the SP stands up and seem as if she wants to leave then turn back and says to the doctor in a pleading tone:

"But doctor I'm really worried… Can you give me amoxyl for my baby? The last time she was sick, it helped."

We developed and finalized the SP script and demanding experiment together with a group of five field supervisors from Kenya, 40 individuals from Kenya who were recruited and hired to be standardized patients for this study (and approximately 60 more who were recruited and underwent partial training but not hired), and a technical advisory group of 4 health care providers who at the time of the study advised on national guidelines and actively trained cadres of health care providers. All of these individuals played a role in days of discussions and exercises during training on what medicines were trusted in the community and whether people in the community are open to using them. SPs and supervisors were involved in piloting the demanding of inappropriate medicines in the field. The team together acknowledged that amoxicillin and albendazole were common medicines, and their selection for study was not done arbitrarily. Further, we conducted the SP pilot with SPs demanding these two medicines

For peer review only - http://bmjopen.bmj.com/site/about/guidelines.xhtml

before the actual study. The selection of these two medicines in the script above were the result of the training and piloting process.

From the experience before fieldwork for this study, the SP recruits, supervisors, and our technical advisory group did not find that it was uncommon for patients in Kenya to ask for specific medicines they are familiar with. In particular, amoxicillin and albendazole are commonly prescribed drugs in the study setting, and thus presumed patients demanding either of those would not be seen as suspicious. It should be noted that the SP scripts were developed while taking into account local habits and behaviors in order to minimize the risk of SPs being identified as simulated, standardized patients.

When we began piloting the demanding experiment before fieldwork, we did not have the first two time points ((i) when the provider writes a prescription or is about to dispense drugs, (ii) when the provider asks what the patient wants). We only had the third (at the end of the interaction). However, the pilot anecdotally demonstrated to us that some providers did (i) and (ii) in the same moment, and for the SPs, it was unusual and out of their character to not respond if they came in "wanting the medicine they demanded".

It is quite possible that demanding a medicine when the provider is writing a prescription or about to dispense drugs could have an underlining incentive-induced difference. In this study, we assume that the different time points for demanding are balanced across each demanding arm.

A3.4 SP Fieldwork – Childhood Diarrhea Experiment Sample Size Calculations

There is some anecdotal evidence that suggests patients can be empowered with correct information to demand better services. At the same time, patients can demand unnecessary or potentially harmful care, such as broad-spectrum antibiotics. Our research question in this study examines how quality of care outcomes change if the patient demands inappropriate services (medicines) for the childhood diarrhea case scenario. This study was added to the endline data collection activities of the AHME program impact evaluation, which aimed to capture differences in quality of care due to AHME with SPs. To calculate MDE under different sample size and AHME program treatment effect scenarios, we utilized quality of care measures from a published SP study and included them as a benchmark for baseline measures and then chose sample sizes that made a best estimate of how much we would expect those outcomes of interest to move.

To calculate sample sizes, we conducted power calculations with minimum detectable effect (MDE) reported differences for a 1:1 randomly allocated SP demanding experiment to clinics independently randomized to receive the AHME program, see Appendix Table A2 below. MDE calculations assume 80% power, 5% alpha, varied differences between non-stratified and stratified control group taking on values {-0.10, -0.05, 0, 0.05, 0.10}, and are based on Daniels et al. (2017) who estimated the following correct management outcomes for private health facilities in Nairobi: 82% (SE: 7%) manage an asthma SP case with an inhaler or bronchodilator and 78% (SE: 8%) manage a childhood diarrhea SP case with oral rehydration salts. The quality of care differences we would be able to detect for the childhood diarrhea experiment would be 9-16%, respectively. For understanding differences in demanding unnecessary care vs. not across AHME treatment and control arms, we expected to answer this question with the original clinic sample with one visit per clinic.

Appendix Table A2. Power and Minimum Detectable Effect (MDE) Calculations

SP case	Power	Observations per Clinic	Total Clinics	Non-stratified	Varied Difference from Control	Control Proportion	MDE	Treatment Proportion

For peer review only - http://bmjopen.bmj.com/site/about/guidelines.xhtml

Diarrhea	0.8	0.2	47	0.78	-0.1	0.68	0.30	0.98
Diarrhea	0.8	0.5	117	0.78	-0.1	0.68	0.21	0.89
Diarrhea	0.8	1	234	0.78	-0.1	0.68	0.16	0.84
Diarrhea	0.8	1.5	351	0.78	-0.1	0.68	0.13	0.81
Diarrhea	0.8	2	468	0.78	-0.1	0.68	0.11	0.79
Diarrhea	0.8	0.2	47	0.78	-0.05	0.73	0.27	1.00
Diarrhea	0.8	0.5	117	0.78	-0.05	0.73	0.19	0.92
Diarrhea	0.8	1	234	0.78	-0.05	0.73	0.14	0.87
Diarrhea	0.8	1.5	351	0.78	-0.05	0.73	0.12	0.85
Diarrhea	0.8	2	468	0.78	-0.05	0.73	0.11	0.84
Diarrhea	0.8	0.2	561	0.78	0	0.78	0.10	0.88
Diarrhea	0.8	0.5	117	0.78	0	0.78	0.17	0.95
Diarrhea	0.8	1	234	0.78	0	0.78	0.13	0.91
Diarrhea	0.8	1.5	351	0.78	0	0.78	0.11	0.89
Diarrhea	0.8	2	468	0.78	0	0.78	0.10	0.88
Diarrhea	0.8	0.2	561	0.78	0.05	0.83	0.09	0.92
Diarrhea	0.8	0.5	117	0.78	0.05	0.83	0.15	0.98
Diarrhea	0.8	1	234	0.78	0.05	0.83	0.11	0.94
Diarrhea	0.8	1.5	351	0.78	0.05	0.83	0.10	0.93
Diarrhea	0.8	2	468	0.78	0.05	0.83	0.09	0.92
Diarrhea	0.8	0.2	561	0.78	0.1	0.88	0.08	0.96
Diarrhea	0.8	0.5	561	0.78	0.1	0.88	0.08	0.96
Diarrhea	0.8	1	234	0.78	0.1	0.88	0.09	0.97
Diarrhea	0.8	1.5	351	0.78	0.1	0.88	0.08	0.96
Diarrhea	0.8	2	468	0.78	0.1	0.88	0.07	0.95

For peer review only

A4 Ethical Clearance

Ethical considerations for utilizing the SP method for this study

The AHME quantitative evaluation was granted clearance by the ethics committees at Kenya Medical Research Institute (No. KEMRI/RES/7/3/1; NON-SSC PROTOCOL NO. 372) and the Human Subjects Committee for Innovations for Poverty Action IRB-USA (IPA IRB Protocol #1085). The ethical clearance included all primary data collection activities for process quality analyses. This appendix describes the protocol for SP data collection.

All the SPs in this study were hired as field staff and participated in a three-week training, and a two-week pilot, and are required to participate in refresher trainings throughout fieldwork in order to mitigate any potentially harmful events, such as unsafe injections, invasive tests, and consumption of any medicines during encounters in the health sector.

Similar to other SP studies with similar designs and embedded in an intervention,[6] we sought a waiver of provider informed consent to conduct the SP study. The request for a waiver was based on a recent study commissioned by the United States Department of Health and Human Services to assess the ethics of simulated patient studies.[7] Supported by a pilot study conducted in Nairobi that validated the SP method in the Kenyan context,[8] both ethics committees approved the waiver request within the AHME evaluation study because (1) combining informed consent with the congregation of providers during trainings and the implementation of interventions during the study period posed threats to the scientific validity of the study objectives, as well as to the risk of SP detection, and (2) there is no more than minimal risk of participation to the SPs or providers, as reported in the Nairobi SP pilot and validation study (Daniels et al. 2017).

Ethics committee approvals with the waiver of informed consent were provided conditional on our agreement to return to all clinics visited by SPs to disclose the SP study to them and to provide them with an opportunity to ask questions and discuss any concerns. During January 1–23, 2020, we informed all clinics that received SPs and that were not closed permanently at that time.

All full questionnaires, case scripts, and the granted request for a waiver of informed provider consent are available upon request.

For peer review only

A5 Fieldwork Protocol and Details

Clinics were assessed for SP visit eligibility and mapping was also conducted to determine which clinics had been closed. The following protocol was implemented ahead of fieldwork initiation:

- Ineligible – SPs should not visit any clinics that are labelled as such in the schedule. For example,
  - 2 clinics – do not accept walk-ins from individuals who are not employees of a firm only
  - 1 clinic – fistula clinic
  - 1 clinic – excluded due to security issues
  - There could be more.
- Check closed status – Supervisors should double check whether these are closed at the time of fieldwork before sending any SPs
  - 8 clinics based on mapping activity
  - There could be more.
- Non-consenting clinics – Supervisors should check mapping and other surveys implemented for the AHME impact evaluation (exit interviews; clinic surveys) to see whether consent has been provided for at least one of these AHME surveys at the time of SP fieldwork. If consent has not been provided at any of these, do not conduct any SP interactions.

To reduce SP detection at the clinic while maintaining fieldwork protocol, the following were implemented:

1. Familiars at sampled clinics. Before conducting any interactions in each region, QAs should introduce the full clinic list to the SPs. The clinics should be described one-by-one. SPs should review all the clinics in the sample and identify to the QA any clinics where they know a friend or family member who is affiliated to that clinic. The QA.
2. SP case narratives. When a team arrives at a new fieldwork area, the QA should debrief the full team on: (i) the area, (ii) the clinics in the area, (iii) contextual adaptations, including dress and language, to each of the cases based on the new fieldwork.
3. Isolated/rural clinics. Isolated/rural clinics should be identified by each QA from their mapping experience. For each SP that goes to that clinic, a story should be constructed for three things: (i) from where the SP character is traveling, (ii) to where the SP character is traveling, (iii) local names of people and places the SP character visited or will visit.
4. SP visit timing. The childhood diarrhea case should be sent in the afternoon.
5. SP sequencing. SP cases or SPs who have a lower risk of detection should be sent before SP cases or SPs who have a higher risk of detection.
6. SP spacing. The spacing between SP visits should be controlled by the QA. First, more than one SP should not be sent at the same time unless the QA knows that there are 5 or more patients waiting to be seen at a given time on a given day. Second, QAs should wait 2-3 days between SP interactions at clinics that see <5 patients per day or do not have strangers coming for services.

For peer review only - http://bmjopen.bmj.com/site/about/guidelines.xhtml

APPENDIX B: SUPPLEMENTAL RESULTS

Appendix Figure B1. Childhood diarrhea knowledge vs. practice

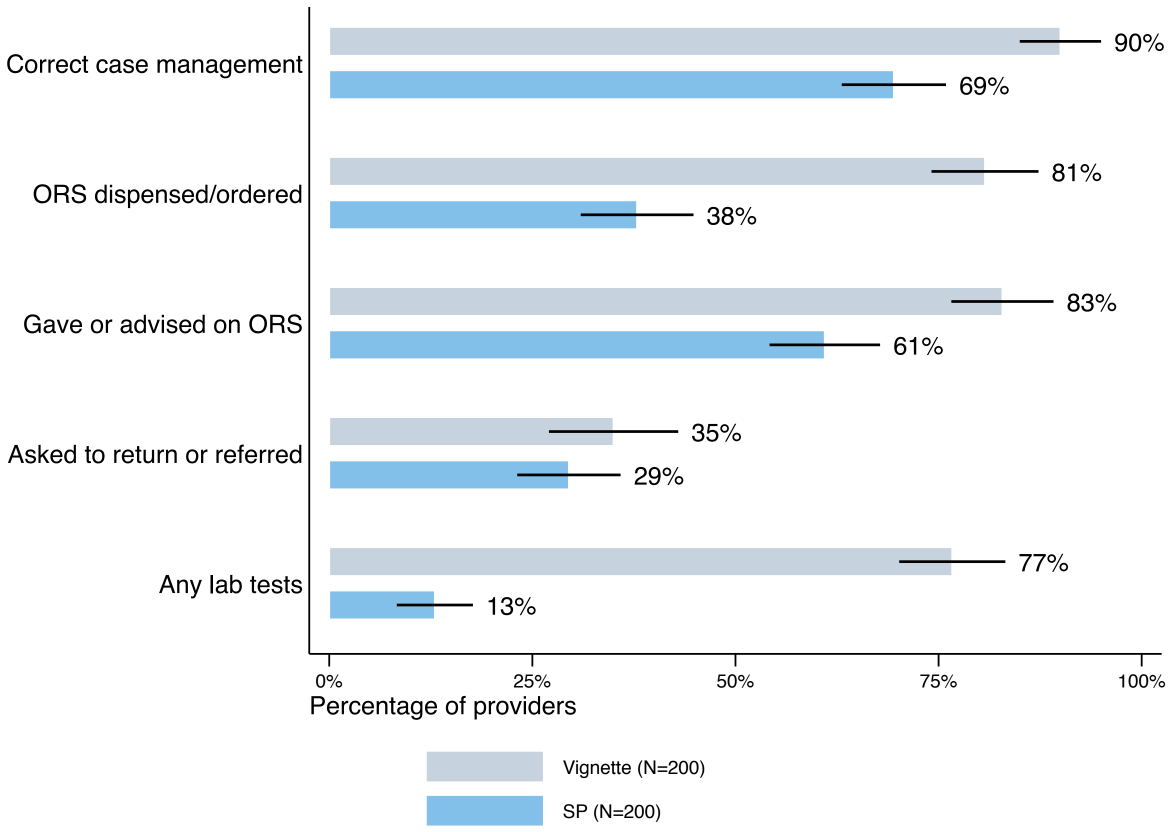

only

For peer review only - http://bmjopen.bmj.com/site/about/guidelines.xhtml

Appendix Figure B2. Effects Pre-demanding Amoxicillin vs. Pre-Demanding Albendazole (n = 200)

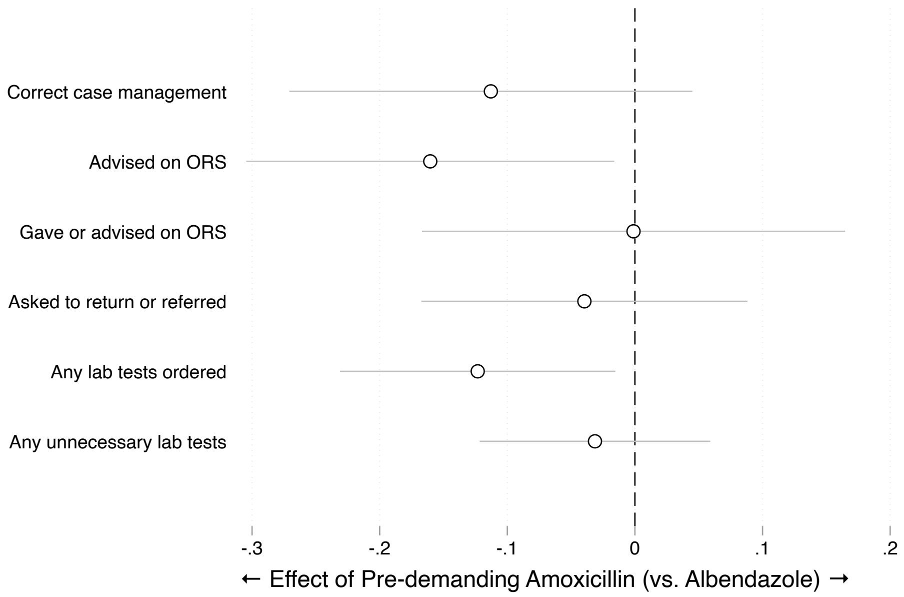

Colored markers indicate signifcant Benjamini-Hochberg p-value at FWER α = .05.

Appendix Figure B3. Effects of Demanding (Pooled Albendazole and Amoxicillin, n = 400)

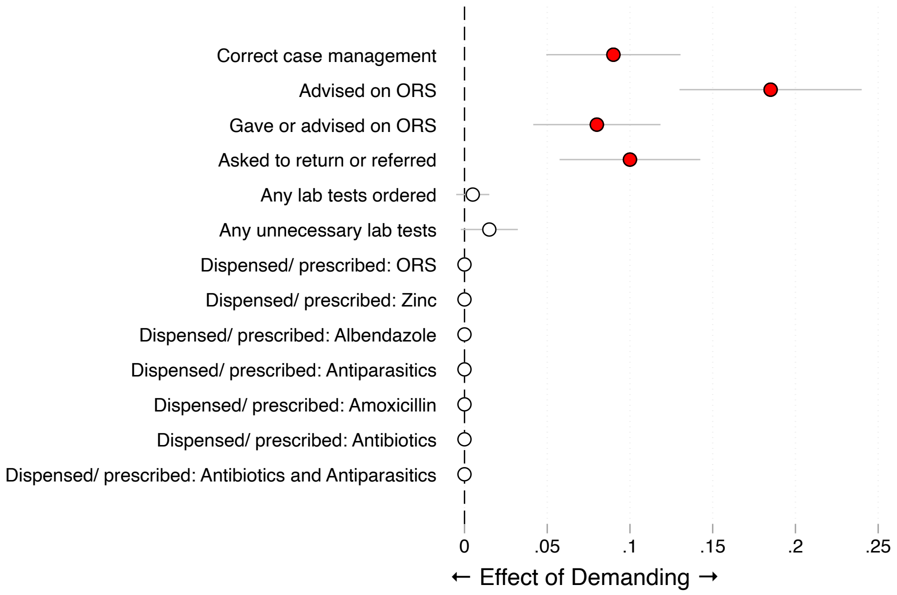

Colored markers indicate signifcant p-value at α = .05.

Appendix Table B1. Effects of demanding albendazole or amoxicillin vs. pre-demanding on quality of care outcomes

(A) Post-demanding (n=200) and pre-demanding (n=200) without AHME and demanding interactions

	(1) Correct case management	(2) Asked to return or referred	(3) Asked to return	(4) Referred elsewhere	(5) Gave or advised on ORS	(6) Advised on ORS	(7) Dispensed/ prescribed: ORS	(8) Dispensed/ prescribed: Zinc	(9) Number of medicines
Albendazole post-demanding									
Coefficient	0.117	0.110	0.123	0.014	0.053	0.198	-0.035	-0.060	0.010
Standard Error	(0.036)	(0.038)	(0.038)	(0.015)	(0.038)	(0.042)	(0.032)	(0.032)	(0.134)
p-value	[0.001]	[0.004]	[0.001]	[0.352]	[0.166]	[0.000]	[0.279]	[0.063]	[0.939]
Amoxicillin post-demanding									
Coefficient	0.062	0.090	0.076	-0.015	0.108	0.171	0.037	0.063	-0.011
Standard Error	(0.038)	(0.040)	(0.040)	(0.016)	(0.041)	(0.047)	(0.034)	(0.034)	(0.140)
p-value	[0.101]	[0.026]	[0.060]	[0.351]	[0.009]	[0.000]	[0.281]	[0.065]	[0.939]
AHME treatment									
Coefficient	-0.009	0.024	0.093	-0.001	-0.069	-0.167	0.085	0.007	0.027
Standard Error	(0.064)	(0.056)	(0.057)	(0.033)	(0.069)	(0.061)	(0.069)	(0.071)	(0.278)
p-value	[0.886]	[0.669]	[0.105]	[0.985]	[0.313]	[0.006]	[0.221]	[0.923]	[0.923]
Observations	400	400	400	400	400	400	400	400	400
Pre-demanding Group Mean	0.705	0.245	0.255	0.060	0.570	0.448	0.360	0.390	2.375

	(10) Number of efficacious medicines	(11) Number of non-efficacious medicines	(12) Any non-efficacious medicines	(13) Dispensed/ prescribed: Albendazole	(14) Dispensed/ prescribed: Amoxicillin	(15) Dispensed/ prescribed: Antibiotics	(16) Dispensed/ prescribed: Antiparasitics	(17) Dispensed/ prescribed: Antibiotics & Antiparasitics
Albendazole post-demanding								
Coefficient	-0.096	0.106	-0.015	0.142	-0.017	0.008	0.122	0.094
Standard Error	(0.060)	(0.105)	(0.033)	(0.027)	(0.019)	(0.034)	(0.029)	(0.027)
p-value	[0.112]	[0.315]	[0.638]	[0.000]	[0.383]	[0.810]	[0.000]	[0.001]
Amoxicillin post-demanding								
Coefficient	0.100	-0.110	0.016	-0.148	0.018	-0.009	-0.127	-0.098
Standard Error	(0.063)	(0.109)	(0.034)	(0.027)	(0.020)	(0.036)	(0.029)	(0.028)
p-value	[0.114]	[0.313]	[0.640]	[0.000]	[0.383]	[0.810]	[0.000]	[0.000]
AHME treatment								
Coefficient	0.092	-0.065	-0.104	-0.003	0.004	-0.030	-0.056	-0.058
Standard Error	(0.127)	(0.217)	(0.068)	(0.054)	(0.040)	(0.072)	(0.061)	(0.057)
p-value	[0.471]	[0.765]	[0.127]	[0.959]	[0.913]	[0.680]	[0.353]	[0.314]
Observations	400	400	400	400	400	400	400	400
Pre-demanding Group Mean	0.750	1.625	0.670	0.190	0.095	0.555	0.245	0.205

Note: The table shows ordinary least squares regressions using standardized patient (SP) data. Robust standard errors are in parentheses, clustered at the clinic level (2 observations corresponding to 1 SP visit per clinic). Two-sided p-values in brackets. All models contain SP fixed effects and control for the 0-1 AHME treatment indicator, a binary indicator for whether a clinic was assigned to receive an SP demanding albendazole at the end of the visit (Albendazole post-demanding) or whether a clinic was assigned to receive an SP demanding amoxicillin at the end of the visit (Amoxicillin post-demanding). All outcomes in models (1)-(17) are binary variables where if the action occurred during the visit 1=yes; 0=otherwise for both pre-demanding and post-demanding time points for the visit. Correct case management is a binary outcome for whether any one of the following actions were performed according to guidelines: asked to return, referred elsewhere, gave ORS, or advised on ORS. ORS is oral rehydration salts. Antiparasitics include antimalarials. "Dispensed/prescribed: Antibiotics & Antiparasitics" refers to whether the provider gave any antibiotic and any antiparasitic.

(B) Post-demanding (n=200) and pre-demanding (n=200) with AHME and demanding interactions

	(1) Correct case management	(2) Asked to return or referred	(3) Asked to return	(4) Referred elsewhere	(5) Gave or advised on ORS	(6) Advised on ORS	(7) Dispensed/ prescribed: ORS	(8) Dispensed/ prescribed: Zinc	(9) Number of medicines
Albendazole post-demanding									
Coefficient	0.132	0.098	0.112	0.014	0.092	0.218	-0.059	-0.110	-0.254
Standard Error	(0.059)	(0.059)	(0.057)	(0.027)	(0.063)	(0.063)	(0.056)	(0.059)	(0.214)
p-value	[0.027]	[0.100]	[0.053]	[0.592]	[0.144]	[0.001]	[0.299]	[0.063]	[0.237]
Albendazole * AHME treatment									
Coefficient	-0.027	0.023	0.020	-0.001	-0.067	-0.033	0.040	0.084	0.444
Standard Error	(0.073)	(0.077)	(0.077)	(0.032)	(0.077)	(0.086)	(0.070)	(0.071)	(0.275)
p-value	[0.718]	[0.768]	[0.794]	[0.981]	[0.384]	[0.700]	[0.569]	[0.242]	[0.109]
Amoxicillin post-demanding									
Coefficient	0.067	0.057	0.046	-0.011	0.099	0.171	0.045	0.085	0.196
Standard Error	(0.052)	(0.049)	(0.048)	(0.021)	(0.055)	(0.065)	(0.043)	(0.045)	(0.165)
p-value	[0.195]	[0.249]	[0.346]	[0.591]	[0.073]	[0.009]	[0.298]	[0.062]	[0.236]
Amoxicillin * AHME treatment									
Coefficient	-0.011	0.069	0.065	-0.007	0.023	0.001	-0.020	-0.050	-0.454
Standard Error	(0.075)	(0.079)	(0.081)	(0.032)	(0.081)	(0.091)	(0.070)	(0.070)	(0.286)
p-value	[0.884]	[0.380]	[0.424]	[0.817]	[0.776]	[0.993]	[0.777]	[0.481]	[0.115]
AHME treatment									
Coefficient	0.000	0.001	0.071	0.001	-0.059	-0.159	0.080	-0.001	0.035
Standard Error	(0.070)	(0.056)	(0.056)	(0.033)	(0.073)	(0.065)	(0.069)	(0.071)	(0.277)
p-value	[0.999]	[0.986]	[0.206]	[0.965]	[0.421]	[0.014]	[0.246]	[0.991]	(0.277)
Observations	400	400	400	400	400	400	400	400	400
Pre-demanding Group Mean	0.705	0.245	0.255	0.060	0.570	0.448	0.360	0.390	2.375

	(10) Number of efficacious medicines	(11) Number of non-efficacious medicines	(12) Any non-efficacious medicines	(13) Dispensed/ prescribed: Albendazole	(14) Dispensed/ prescribed: Amoxicillin	(15) Dispensed/ prescribed: Antibiotics	(16) Dispensed/ prescribed: Antiparasitics	(17) Dispensed/ prescribed: Antibiotics & Antiparasitics
Albendazole post-demanding								
Coefficient	-0.169	-0.085	-0.094	0.176	-0.030	-0.057	0.143	0.118
Standard Error	(0.105)	(0.165)	(0.053)	(0.049)	(0.030)	(0.058)	(0.053)	(0.050)
p-value	[0.111]	[0.607]	[0.080]	[0.000]	[0.321]	[0.329]	[0.008]	[0.019]
Albendazole * AHME treatment								
Coefficient	0.124	0.320	0.132	-0.059	0.022	0.110	-0.037	-0.042
Standard Error	(0.129)	(0.216)	(0.067)	(0.058)	(0.039)	(0.072)	(0.063)	(0.060)
p-value	[0.339]	[0.140]	[0.050]	[0.311]	[0.579]	[0.129]	[0.559]	[0.479]
Amoxicillin post-demanding								
Coefficient	0.130	0.066	0.072	-0.136	0.023	0.044	-0.111	-0.091
Standard Error	(0.081)	(0.128)	(0.041)	(0.040)	(0.023)	(0.045)	(0.042)	(0.039)
p-value	[0.109]	[0.607]	[0.081]	[0.001]	[0.323]	[0.329]	[0.009]	[0.021]
Amoxicillin * AHME treatment								
Coefficient	-0.070	-0.384	-0.124	-0.023	-0.012	-0.115	-0.033	-0.012
Standard Error	(0.129)	(0.225)	(0.069)	(0.056)	(0.041)	(0.072)	(0.061)	(0.057)
p-value	[0.589]	[0.090]	[0.072]	[0.684]	[0.770]	[0.112]	[0.585]	[0.841]
AHME treatment								
Coefficient	0.080	-0.044	-0.104	0.017	0.002	-0.027	-0.039	-0.044
Standard Error	(0.127)	(0.217)	(0.067)	(0.057)	(0.040)	(0.072)	(0.063)	(0.059)
p-value	[0.531]	[0.839]	[0.124]	[0.764]	[0.955]	[0.709]	[0.538]	[0.451]
Observations	400	400	400	400	400	400	400	400
Pre-demanding Group Mean	0.750	1.625	0.670	0.190	0.095	0.555	0.245	0.205

Note: The table shows ordinary least squares regressions using standardized patient (SP) data. Robust standard errors are in parentheses, clustered at the clinic level (2 observations corresponding to 1 SP visit per clinic). Two-sided p-values in brackets. All models contain SP fixed effects and control for the 0-1 AHME treatment indicator, a binary indicator for whether a clinic was assigned to receive an SP demanding albendazole at the end of the visit (Albendazole post-demanding) or whether a clinic was assigned to receive an SP demanding amoxicillin at the end of the visit (Amoxicillin post-demanding). Models also include interactions between the AHME treatment and each of the demanding experiments. All outcomes in models (1)-(17) are binary variables where if the action occurred during the visit 1=yes; 0=otherwise for both pre-demanding and post-demanding time points for the visit. Correct case management is a binary outcome for whether any one of the following actions were performed according to guidelines: asked to return, referred elsewhere, gave ORS, or advised on ORS. ORS is oral rehydration salts. Antiparasitics include antimalarials. "Dispensed/prescribed: Antibiotics & Antiparasitics" refers to whether the provider gave any antibiotic and any antiparasitic.

For peer review only - http://bmjopen.bmj.com/site/about/guidelines.xhtml

Appendix Table B2. Effects of post-demanding albendazole (vs. post-demanding amoxicillin) on childhood diarrhea care management outcomes

(A) Post-demanding (n=200) without AHME and demanding interactions

	(1) Correct case management	(2) Asked to return or referred	(3) Asked to return	(4) Referred elsewhere	(5) Gave or advised on ORS	(6) Advised on ORS	(7) Dispensed/ prescribed: ORS	(8) Dispensed/ prescribed: Zinc	(9) Number of medicines
Albendazole post-demanding									
Coefficient	0.052	0.014	0.046	0.034	-0.074	-0.015	-0.086	-0.148	0.025
Standard Error	(0.073)	(0.076)	(0.077)	(0.040)	(0.081)	(0.080)	(0.081)	(0.081)	(0.331)
p-value	[0.473]	[0.853]	[0.553]	[0.391]	[0.360]	[0.854]	[0.287]	[0.071]	[0.940]
AHME treatment									
Coefficient	-0.022	0.049	0.115	-0.003	-0.083	-0.168	0.092	0.018	0.025
Standard Error	(0.064)	(0.067)	(0.068)	(0.035)	(0.071)	(0.071)	(0.071)	(0.072)	(0.292)
p-value	[0.727]	[0.466]	[0.091]	[0.926]	[0.248]	[0.019]	[0.200]	[0.800]	[0.932]
Observations	200	200	200	200	200	200	200	200	200
Demanding Amoxicillin Group Mean	0.755	0.296	0.286	0.051	0.673	0.582	0.398	0.449	2.347

	(10) Number of efficacious medicines	(11) Number of non-efficacious medicines	(12) Any non-efficacious medicines	(13) Dispensed/ prescribed: Albendazole	(14) Dispensed/ prescribed: Amoxicillin	(15) Dispensed/ prescribed: Antibiotics	(16) Dispensed/ prescribed: Antiparasitics	(17) Dispensed/ prescribed: Antibiotics & Antiparasitics
Albendazole post-demanding								
Coefficient	-0.234	0.259	-0.038	0.348	-0.041	0.020	0.299	0.230
Standard Error	(0.147)	(0.257)	(0.078)	(0.059)	(0.049)	(0.084)	(0.068)	(0.066)
p-value	[0.113]	[0.314]	[0.631]	[0.000]	[0.397]	[0.810]	[0.000]	[0.001]
AHME treatment								
Coefficient	0.110	-0.085	-0.101	-0.030	0.008	-0.031	-0.080	-0.075
Standard Error	(0.130)	(0.226)	(0.069)	(0.052)	(0.043)	(0.074)	(0.060)	(0.058)
p-value	[0.398]	[0.708]	[0.147]	[0.570]	[0.859]	[0.671]	[0.188]	[0.197]
Observations	200	200	200	200	200	200	200	200
Demanding Amoxicillin Group Mean	0.847	1.500	0.704	0.031	0.112	0.551	0.133	0.122

Note: The table shows ordinary least squares regressions using standardized patient (SP) data for the post-demanding phase of the N=200 SP visits (1 observation corresponds to 1 SP visit per clinic). Standard errors are in parentheses. Two-sided p-values in brackets. All models contain SP fixed effects and control for the 0-1 AHME treatment indicator, a binary indicator for whether the visit was Albendazole post-demanding (if 0, the visit was Amoxicillin post-demanding). All outcomes in models (1)-(17) are binary variables where if the action occurred during by the end of the visit 1=yes; 0=otherwise. Correct case management is a binary outcome for whether any one of the following actions were performed according to guidelines: asked to return, referred elsewhere, gave ORS, or advised on ORS. ORS is oral rehydration salts. Antiparasitics include antimalarials. "Dispensed/prescribed: Antibiotics & Antiparasitics" refers to whether the provider gave any antibiotic and any antiparasitic.

For peer review only - http://bmjopen.bmj.com/site/about/guidelines.xhtml

(B) Post-demanding (n=200) with AHME and demanding interactions

	(1) Correct case management	(2) Asked to return or referred	(3) Asked to return	(4) Referred elsewhere	(5) Gave or advised on ORS	(6) Advised on ORS	(7) Dispensed/ prescribed: ORS	(8) Dispensed/ prescribed: Zinc	(9) Number of medicines
Albendazole post-demanding									
Coefficient	0.064	0.033	0.064	0.031	-0.022	0.006	-0.117	-0.217	-0.450
Standard Error	(0.099)	(0.102)	(0.104)	(0.054)	(0.109)	(0.109)	(0.109)	(0.110)	(0.445)
p-value	[0.519]	[0.745]	[0.541]	[0.572]	[0.840]	[0.954]	[0.284]	[0.049]	[0.313]
Albendazole * AHME treatment									
Coefficient	-0.022	-0.037	-0.034	0.007	-0.100	-0.040	0.060	0.134	0.913
Standard Error	(0.127)	(0.132)	(0.134)	(0.070)	(0.141)	(0.140)	(0.141)	(0.142)	(0.574)
p-value	[0.865]	[0.778]	[0.797]	[0.918]	[0.478]	[0.774]	[0.672]	[0.347]	[0.113]
AHME treatment									
Coefficient	-0.012	0.067	0.132	-0.007	-0.034	-0.148	0.062	-0.047	-0.420
Standard Error	(0.089)	(0.093)	(0.094)	(0.049)	(0.099)	(0.099)	(0.099)	(0.100)	(0.404)
p-value	[0.894]	[0.472]	[0.164]	[0.890]	[0.733]	[0.135]	[0.528]	[0.639]	[0.300]
Observations	200	200	200	200	200	200	200	200	200
Demanding Amoxicillin Group Mean	0.755	0.296	0.286	0.051	0.673	0.582	0.398	0.449	2.347

	(10) Number of efficacious medicines	(11) Number of non-efficacious medicines	(12) Any non-efficacious medicines	(13) Dispensed/ prescribed: Albendazole	(14) Dispensed/ prescribed: Amoxicillin	(15) Dispensed/ prescribed: Antibiotics	(16) Dispensed/ prescribed: Antiparasitics	(17) Dispensed/ prescribed: Antibiotics & Antiparasitics
Albendazole post-demanding								
Coefficient	-0.335	-0.115	-0.173	0.365	-0.059	-0.099	0.299	0.244
Standard Error	(0.198)	(0.345)	(0.105)	(0.080)	(0.066)	(0.113)	(0.092)	(0.089)
p-value	[0.094]	[0.738]	[0.101]	[0.000]	[0.373]	[0.380]	[0.001]	[0.007]
Albendazole * AHME treatment								
Coefficient	0.193	0.720	0.260	-0.031	0.034	0.229	0.001	-0.028
Standard Error	(0.256)	(0.444)	(0.135)	(0.104)	(0.085)	(0.145)	(0.119)	(0.115)
p-value	[0.451]	[0.107]	[0.056]	[0.763]	[0.693]	[0.116]	[0.994]	[0.808]
AHME treatment								
Coefficient	0.016	-0.436	-0.227	-0.015	-0.009	-0.143	-0.080	-0.062
Standard Error	(0.180)	(0.313)	(0.095)	(0.073)	(0.060)	(0.102)	(0.084)	(0.081)
p-value	[0.931]	[0.165]	[0.018]	[0.842]	[0.884]	[0.163]	[0.341]	[0.446]
Observations	200	200	200	200	200	200	200	200
Demanding Amoxicillin Group Mean	0.847	1.500	0.704	0.031	0.112	0.551	0.133	0.122

Note: The table shows ordinary least squares regressions using standardized patient (SP) data for the post-demanding phase of the N=200 SP visits (1 observation corresponds to 1 SP visit per clinic). Standard errors are in parentheses. Two-sided p-values in brackets. All models contain SP fixed effects and control for the 0-1 AHME treatment indicator, a binary indicator for whether the visit was Albendazole post-demanding (if 0, the visit was Amoxicillin post-demanding). Models also include interactions between the AHME treatment and the Albendazole post-demanding experiment. All outcomes in models (1)-(17) are binary variables where if the action occurred during by the end of the visit 1=yes; 0=otherwise. Correct case management is a binary outcome for whether any one of the following actions were performed according to guidelines: asked to return, referred elsewhere, gave ORS, or advised on ORS. ORS is oral rehydration salts. Antiparasitics include antimalarials. "Dispensed/prescribed: Antibiotics & Antiparasitics" refers to whether the provider gave any antibiotic and any antiparasitic.

For peer review only - http://bmjopen.bmj.com/site/about/guidelines.xhtml

Appendix Table B3. Composition of medicines prescribed across all SPs who demanded albendazole versus amoxicillin.

Type	Medicine	Demanding Albendazole (N=102)		Demanding Amoxicillin (N=98)	
		Frequency	*Percentage*	*Frequency*	*Percentage*
Correct	ORAL REHYDRATION SALTS	32	31%	38	39%
	ZINC	33	32%	42	43%
	ORAL REHYDRATION SALTS AND ZINC SULPHATE	2	2%	2	2%
Antiparasitic	ALBENDAZOLE	35	34%	3	3%
	ARTEMETHER LUMEFANTRINE	5	5%	4	4%
	QUININE	1	1%	0	0%
	DILOXANIDE	0	0%	4	4%
	DIHYDROARTEMISININ AND PIPERAQUINE PHOSPHATE	0	0%	1	1%
	NITAZOXANIDE	0	0%	1	1%
Antibiotic	METRONIDAZOLE	27	26%	27	28%
	SULFAMETHOXAZOLE AND TRIMETHOPRIM	17	17%	21	21%
	METRONIDAZOLE BENZOATE	12	12%	12	12%
	AMOXICILLIN	8	8%	11	11%
	DILOXANIDE FUROATE METRONIDAZOLE DICYCLOMINE HCL	6	6%	2	2%
	AMPICILLIN AND CLOXACILLIN	3	3%	3	3%
	CEFALEXIN	2	2%	2	2%
	ERYTHROMYCIN	2	2%	2	2%
	CEFIXIME	2	2%	0	0%
	CHLORAMPHENICOL PALMITATE	1	1%	2	2%
	AMOXICILLIN AND POTASSIUM CLAVULANATE	1	1%	0	0%
	CIPROFLOXACIN	1	1%	0	0%
	CHLORAMPHENICOL	1	1%	0	0%
	DILOXANIDE FUROATE METRONIDAZOLE	1	1%	0	0%
	ERYTHROMYCIN ETHYL SUCCINATE	1	1%	0	0%
	CHLORAPHENICOL AND RETINOL	0	0%	1	1%
	ROXITHROMYCIN	0	0%	1	1%
	AMPICILLIN	0	0%	1	1%
	CEFADROXIL	0	0%	1	1%

For peer review only - http://bmjopen.bmj.com/site/about/guidelines.xhtml

Other	PARACETAMOL	22	22%	23	23%
	IBUPROFEN	5	5%	3	3%
	LEVAMISOLE	4	4%	0	0%
	IBUPROFEN AND PARACETAMOL	3	3%	1	1%
	HYOSCINE BUTYLBROMIDE	2	2%	0	0%
	PROMETHAZINE HYDROCHLORIDE	1	1%	5	5%
	SACCHAROMYCES BOULARDII	1	1%	1	1%
	MULTIVITAMIN	1	1%	1	1%
	KAOLIN PECTIN	1	1%	0	0%
	AMINOSIDINE	1	1%	0	0%
	CHLORPHENIRAMINE	1	1%	0	0%
	CETIRIZINE	1	1%	0	0%
	DICYCLOVERINE HYDROCHLORIDE SIMETHICONE	1	1%	0	0%
	GUAIFENESIN	1	1%	0	0%
	VITAMIN A	1	1%	0	0%
	LOPERAMID HYDROCHLORIDE	0	0%	2	2%
	DOMPERIDONE	0	0%	1	1%
	PIROXICAM	0	0%	1	1%
	SALBUTAMOL	0	0%	1	1%
Unknown	UNKNOWN	0	0%	1	1%

For peer review only

For peer review only - http://bmjopen.bmj.com/site/about/guidelines.xhtml

CONSORT 2010 checklist of information to include when reporting a randomised trial*

Section/Topic	Item No	Checklist item	Reported on page No
**Title and abstract**			
	1a	Identification as a randomised trial in the title	1
	1b	Structured summary of trial design, methods, results, and conclusions (for specific guidance see CONSORT for abstracts)	1
**Introduction**			
Background and objectives	2a	Scientific background and explanation of rationale	6,7
	2b	Specific objectives or hypotheses	4
**Methods**			
Trial design	3a	Description of trial design (such as parallel, factorial) including allocation ratio	6
	3b	Important changes to methods after trial commencement (such as eligibility criteria), with reasons	Appendix S13-14
Participants	4a	Eligibility criteria for participants	6
	4b	Settings and locations where the data were collected	6, Figure 1
Interventions	5	The interventions for each group with sufficient details to allow replication, including how and when they were actually administered	6
Outcomes	6a	Completely defined pre-specified primary and secondary outcome measures, including how and when they were assessed	7
	6b	Any changes to trial outcomes after the trial commenced, with reasons	N/A
Sample size	7a	How sample size was determined	Appendix S11-S12
	7b	When applicable, explanation of any interim analyses and stopping guidelines	N/A
Randomisation:			
Sequence generation	8a	Method used to generate the random allocation sequence	6
	8b	Type of randomisation; details of any restriction (such as blocking and block size)	6
Allocation concealment mechanism	9	Mechanism used to implement the random allocation sequence (such as sequentially numbered containers), describing any steps taken to conceal the sequence until interventions were assigned	6
Implementation	10	Who generated the random allocation sequence, who enrolled participants, and who assigned participants to	Appendix

For peer review only - http://bmjopen.bmj.com/site/about/guidelines.xhtml

		interventions	
Blinding	11a	If done, who was blinded after assignment to interventions (for example, participants, care providers, those assessing outcomes) and how	Appendix S13
	11b	If relevant, description of the similarity of interventions	N/A
Statistical methods	12a	Statistical methods used to compare groups for primary and secondary outcomes	pp. 7-8
	12b	Methods for additional analyses, such as subgroup analyses and adjusted analyses	pp. 7-8
**Results**			
Participant flow (a diagram is strongly recommended)	13a	For each group, the numbers of participants who were randomly assigned, received intended treatment, and were analysed for the primary outcome	Figure 1
	13b	For each group, losses and exclusions after randomisation, together with reasons	Figure 1
Recruitment	14a	Dates defining the periods of recruitment and follow-up	pp. 6-7, Appendix S19
	14b	Why the trial ended or was stopped	N/A
Baseline data	15	A table showing baseline demographic and clinical characteristics for each group	Table 2
Numbers analysed	16	For each group, number of participants (denominator) included in each analysis and whether the analysis was by original assigned groups	All tables
Outcomes and estimation	17a	For each primary and secondary outcome, results for each group, and the estimated effect size and its precision (such as 95% confidence interval)	Figure 2
	17b	For binary outcomes, presentation of both absolute and relative effect sizes is recommended	Figure 2, Table 3, Appendix B Tables and Figures
Ancillary analyses	18	Results of any other analyses performed, including subgroup analyses and adjusted analyses, distinguishing pre-specified from exploratory	Appendix B Tables and Figures
Harms	19	All important harms or unintended effects in each group (for specific guidance see CONSORT for harms)	Appendix S13-14
**Discussion**			
Limitations	20	Trial limitations, addressing sources of potential bias, imprecision, and, if relevant, multiplicity of analyses	pp. 10-12
Generalisability	21	Generalisability (external validity, applicability) of the trial findings	p. 12
Interpretation	22	Interpretation consistent with results, balancing benefits and harms, and considering other relevant evidence	pp. 10-12

For peer review only - http://bmjopen.bmj.com/site/about/guidelines.xhtml

**Other information**			
Registration	23	Registration number and name of trial registry	p. 8
Protocol	24	Where the full trial protocol can be accessed, if available	p. 8
Funding	25	Sources of funding and other support (such as supply of drugs), role of funders	p. 13

*We strongly recommend reading this statement in conjunction with the CONSORT 2010 Explanation and Elaboration for important clarifications on all the items. If relevant, we also recommend reading CONSORT extensions for cluster randomised trials, non-inferiority and equivalence trials, non-pharmacological treatments, herbal interventions, and pragmatic trials. Additional extensions are forthcoming: for those and for up to date references relevant to this checklist, see www.consort-statement.org.

For peer review only
